# Zika Virus Infection as a Cause of Congenital Brain Abnormalities and Guillain–Barré Syndrome: Systematic Review

**DOI:** 10.1371/journal.pmed.1002203

**Published:** 2017-01-03

**Authors:** Fabienne Krauer, Maurane Riesen, Ludovic Reveiz, Olufemi T. Oladapo, Ruth Martínez-Vega, Teegwendé V. Porgo, Anina Haefliger, Nathalie J. Broutet, Nicola Low, WHO Zika Causality Working Group

**Affiliations:** 1 Institute of Social and Preventive Medicine, University of Bern, Switzerland; 2 Pan American Health Organization, Washington DC, United States of America; 3 UNDP/UNFPA/UNICEF/WHO/World Bank Special Programme of Research, Development and Research Training in Human Reproduction (HRP), Department of Reproductive Health and Research, World Health Organization, Geneva, Switzerland; 4 Escuela de Microbiologia, Universidad Industrial de Santander, Santander, Colombia; 5 Department of Social and Preventative Medicine, Laval University, Québec, Canada; Mahidol-Oxford Tropical Medicine Research Unit, THAILAND

## Abstract

**Background:**

The World Health Organization (WHO) stated in March 2016 that there was scientific consensus that the mosquito-borne Zika virus was a cause of the neurological disorder Guillain–Barré syndrome (GBS) and of microcephaly and other congenital brain abnormalities based on rapid evidence assessments. Decisions about causality require systematic assessment to guide public health actions. The objectives of this study were to update and reassess the evidence for causality through a rapid and systematic review about links between Zika virus infection and (a) congenital brain abnormalities, including microcephaly, in the foetuses and offspring of pregnant women and (b) GBS in any population, and to describe the process and outcomes of an expert assessment of the evidence about causality.

**Methods and Findings:**

The study had three linked components. First, in February 2016, we developed a causality framework that defined questions about the relationship between Zika virus infection and each of the two clinical outcomes in ten dimensions: temporality, biological plausibility, strength of association, alternative explanations, cessation, dose–response relationship, animal experiments, analogy, specificity, and consistency. Second, we did a systematic review (protocol number CRD42016036693). We searched multiple online sources up to May 30, 2016 to find studies that directly addressed either outcome and any causality dimension, used methods to expedite study selection, data extraction, and quality assessment, and summarised evidence descriptively. Third, WHO convened a multidisciplinary panel of experts who assessed the review findings and reached consensus statements to update the WHO position on causality. We found 1,091 unique items up to May 30, 2016. For congenital brain abnormalities, including microcephaly, we included 72 items; for eight of ten causality dimensions (all except dose–response relationship and specificity), we found that more than half the relevant studies supported a causal association with Zika virus infection. For GBS, we included 36 items, of which more than half the relevant studies supported a causal association in seven of ten dimensions (all except dose–response relationship, specificity, and animal experimental evidence). Articles identified nonsystematically from May 30 to July 29, 2016 strengthened the review findings. The expert panel concluded that (a) the most likely explanation of available evidence from outbreaks of Zika virus infection and clusters of microcephaly is that Zika virus infection during pregnancy is a cause of congenital brain abnormalities including microcephaly, and (b) the most likely explanation of available evidence from outbreaks of Zika virus infection and GBS is that Zika virus infection is a trigger of GBS. The expert panel recognised that Zika virus alone may not be sufficient to cause either congenital brain abnormalities or GBS but agreed that the evidence was sufficient to recommend increased public health measures. Weaknesses are the limited assessment of the role of dengue virus and other possible cofactors, the small number of comparative epidemiological studies, and the difficulty in keeping the review up to date with the pace of publication of new research.

**Conclusions:**

Rapid and systematic reviews with frequent updating and open dissemination are now needed both for appraisal of the evidence about Zika virus infection and for the next public health threats that will emerge. This systematic review found sufficient evidence to say that Zika virus is a cause of congenital abnormalities and is a trigger of GBS.

## Abstract 2

In a systematic review, Nicola Low and colleagues use a causality framework to examine the evidence for zika virus infection as a cause of congenital brain abnormalities and Guillain–Barré syndrome.

## Author Summary

**Why Was This Study Done?:**

- In 2015, the mosquito-borne Zika virus caused epidemics of a mild viral illness for the first time in Brazil and then other countries in Latin America and the Caribbean.
- In mid to late 2015, clinicians in northeastern Brazil reported unexpected increases in the numbers of babies born with abnormally small heads (microcephaly) and of adults with Guillain–Barré syndrome (GBS), a paralytic condition triggered by certain infections.
- In February 2016, the World Health Organization (WHO) declared a Public Health Emergency of International Concern and called for research about the causal relationship between Zika virus and congenital brain abnormalities, including microcephaly, and GBS.

**What Did the Researchers Do and Find?:**

- We developed a causality framework for Zika virus and (a) congenital brain abnormalities, and (b) GBS. For each outcome, we developed specific questions in ten different dimensions of causality: temporality; biological plausibility; strength of association; exclusion of alternative explanations; cessation; dose–response relationship; animal experimental evidence; analogy; specificity; and consistency of findings.
- We did a systematic review of published and unpublished evidence up to May 30, 2016. We summarised the evidence descriptively. A panel of experts assessed the findings and reached a consensus about causality.
- For congenital brain abnormalities, we assessed 72 studies that addressed questions in one or more causality dimensions. Reports of pregnancies affected by Zika virus have come from countries with circulating Zika virus in the Americas, the Pacific region, and West Africa. Clinical reports have documented Zika virus infection in pregnant women followed by foetal abnormalities, particularly with infection in the first trimester. These women did not have any other congenital infection or dengue virus infection. The risk of congenital brain abnormalities could be around 50 times higher in mothers who had Zika virus infection in pregnancy compared with those who did not. In laboratory studies, Zika virus has been shown to cross the placenta and replicate in human brain cells.
- For GBS, we assessed 36 studies that addressed questions about one or more causality dimensions. In several countries in the Americas and the Pacific region, a temporal association has been found, with symptoms of Zika virus infection preceding the onset of GBS. In these countries, surveillance reports of cases of GBS followed the pattern of reports of Zika-like illness. During a Zika virus outbreak in French Polynesia in 2013–14, scientists estimated that around one in 4000 people with Zika virus infection developed GBS. The odds of having had a recent Zika virus infection were more than 30 times higher in patients with GBS than those without in a hospital-based study in French Polynesia. Several other infections that can trigger GBS were excluded.

**What Do These Findings Mean?:**

- This systematic review found sufficient evidence to conclude that Zika virus is a cause of congenital abnormalities and is a trigger of GBS.
- Systematic reviews of evidence about emerging public health threats need to be updated frequently.

## Introduction

An “explosive pandemic of Zika virus infection” [1] in 2015 caught the world by surprise. The Pan American Health Organization (PAHO) and World Health Organization (WHO) published an alert about a possible association with increases in reports of congenital abnormalities and Guillain–Barré syndrome (GBS) on December 1, 2015 [2]. On February 1, 2016, WHO declared a Public Health Emergency of International Concern [3]. Microcephaly at birth is a clinical finding that can include a range of brain malformations resulting from a failure of neurogenesis [4]. Infections acquired in pregnancy, including cytomegalovirus and rubella, are established causes [4]. GBS is an immune-mediated ascending flaccid paralysis, which typically occurs within a month of an infection, such as *Campylobacter jejuni* or cytomegalovirus [5]. As of October 20, 2016, 67 countries have reported autochthonous transmission of the mosquito-borne flavivirus Zika since 2015, and 27 of these countries have reported cases of congenital brain abnormalities, GBS, or both [6]. The emergency committee recommended increased research [3] to provide more rigorous scientific evidence of a causal relationship as a basis for the global health response.

Unexplained clusters of rare but serious conditions require urgent assessment of causality, balancing speed with systematic appraisal. Bradford Hill is widely credited for his proposed framework for thinking about causality in epidemiology, which listed nine “viewpoints” from which to study associations between exposure and disease (S1 Text, p2) [7]. Since then, the list has been modified (S1 Text, p2; S1 Table) [8]. Bradford Hill emphasised that his viewpoints were not rules but, taken together, the body of evidence should be used to decide whether there is any other more likely explanation than cause and effect.

The level of certainty required before judging that Zika virus is a cause of microcephaly and GBS is contentious [9]. Most assessments have been based on rapid but nonsystematic appraisals [10–12]. Based on rapid reviews, WHO has stated that there is “scientific consensus that Zika virus is a cause of microcephaly and GBS” since March 31, 2016 [13]. On April 13, a narrative review stated that there was “a causal relationship between prenatal Zika virus infection and microcephaly” [11]. Evidence about the causal relationship between Zika virus and GBS has not yet been assessed in detail. We previously described a causality framework for Zika virus and plans for a systematic review (S1 Text, p3; S2 Table), with a preliminary overview of 21 studies, published up to March 4, 2016 [14]. The objectives of this study are to reassess the evidence for causality and update the WHO position about links between Zika virus and (a) congenital brain abnormalities, including microcephaly, in the foetuses and offspring of pregnant women and (b) GBS in any population, and to describe the process and outcomes of an expert assessment of the evidence.

## Methods

We describe three linked components: the causality framework, the systematic reviews, and the expert panel assessment of the review findings. The WHO Zika Causality Working Group convened the expert panel of 18 members with specialist knowledge in the fields of epidemiology and public health, virology, infectious diseases, obstetrics, neonatology, and neurology (membership of the expert panel is provided in the Acknowledgments).

### Zika Causality Framework

In February 2016, we developed a causality framework for Zika virus by defining specific questions for each of ten dimensions, modified from Bradford Hill’s list (S2 Table): temporality; biological plausibility; strength of association; exclusion of alternative explanations; cessation; dose–response relationship; animal experimental evidence; analogy; specificity; and consistency of findings. This review covered 35 questions about congenital brain abnormalities, including microcephaly, and 26 questions about GBS. We also listed seven groups of cofactors that might increase the risk of an outcome in the presence of Zika virus [15].

### Systematic Review

Our protocol was registered on March 21, 2016 in the database PROSPERO (CRD42016036693) [16]. We report the methods in full according to the Preferred Reporting Items for Systematic Reviews and Meta-Analyses (PRISMA) [17] in S1 Text (p3-4).

To report our findings, we use the term item for an individual record, e.g., a case report. Occasionally, the same individuals or data were reported in more than one publication (item). To avoid double counting, we organised these items into groups. We chose a primary publication (the item with the most complete information) to represent the group, to which other items were linked (S4 Table, S6 Table).

#### Eligibility

We included studies of any design and in any language that directly addressed any research question in the causality framework (S1 Text, p3).

#### Information sources and search strategy

We searched multiple electronic databases and websites (protocol [16] and S1 Text, p3) and included published peer-reviewed studies, ongoing studies, and non-peer reviewed sources. For the dimension addressing analogous causes of the outcomes and for cofactors, we did not conduct systematic searches.

We conducted our first search from the earliest date to April 11, 2016 and updated the search on May 30 and July 29. We selected items and extracted data systematically on included items up to May 30 and reported on nonsystematically identified studies up to July 29, 2016.

#### Study selection and data extraction

We screened titles, abstracts, and full texts by liberal accelerated screening (S1 Text, p3) [18]. For data extraction, one reviewer extracted data and a second reviewer checked the extracted data. We used case definitions and laboratory diagnostic test interpretations as reported by study authors.

#### Synthesis of findings and assessment of methodological quality

We tabulated study-level data and clinical information from case reports, case series, cross-sectional studies, case-control studies, and cohort studies. We assessed methodological quality for these designs using checklists [19]. Each reviewer recorded an overall judgement to indicate whether study findings did or did not provide support for each causality dimension. We assigned a judgement of sufficient evidence about a causality dimension if the consensus assessments were supportive for at least half of the specific questions. We appraised the body of evidence according to the Grading of Research Assessment Development and Evaluation (GRADE) tool, as suggested for urgent health questions [20], but did not apply upgrading or downgrading because these concepts could not be applied consistently across the range of study designs.

### Expert Panel

**Fig 1:**
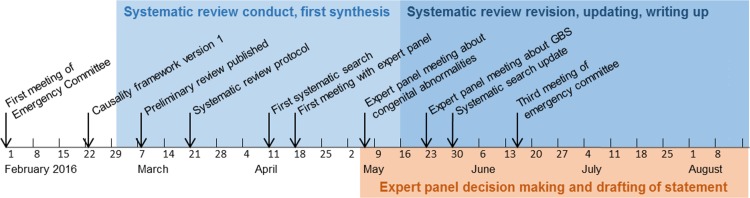
Timeline of Zika causality review, February 1 to August 2016. A Public Health Emergency of International Concern was announced on February 1, 2016 in response to clusters of microcephaly, GBS, and other neurological disorders.

In a series of web and telephone conferences between April 18 and May 23, 2016, we presented our approach to the assessment of causality, the causality framework, and our synthesis of evidence to the expert panel. We discussed these topics with the experts during the conferences and through email discussions. We then drafted summary conclusions about the most likely explanation for the reported clusters of cases of microcephaly and GBS. The expert panel members reached consensus statements to update the WHO position (Fig 1).

## Results

We found 1,091 unique items published from 1952 to May 30, 2016 (S1 Fig, S3 Table). Most excluded items were reviews or editorials and commentaries (44%, *n* = 479). We included 106 items from 87 groups (Table 1), of which 83% were published in 2016. For both outcomes, the majority of items were clinical, individual-level case reports, case series, or population-level surveillance data.

**Table 1:** Summary of included items according to outcome, study design, and causality dimension.

	Congenital abnormalities		GBS	
	N	%	N	%
**Type of study**				
Case report	9	12.5	9	25
Case series	22	30.6	5	13.9
Case-control study	0	0	1	2.8
Cohort study	1	1.4	0	0
Cross-sectional study	2^a^	2.8	0	0
Ecological study/outbreak report	5	6.9	19	52.8
Modelling study	2	2.8	0	0
Animal experiment	18	25	0	0
In vitro experiment	10	13.9	0	0
Sequence analysis and phylogenetics	3	4.2	2	5.6
**Total items**	**72**	**100**	**36**	**100**
**Causality dimension^b^**				
Temporality	21	36.2	26	83.9
Biological plausibility	25	43.1	4	12.9
Strength of association	3	5.2	2	6.5
Alternative explanation	18	31	6	19.4
Cessation	2	3.4	6	19.4
Dose–response relationship	0	0	0	0
Experiment	20	34.5	0	0
Analogy	NA	NA	NA	NA
Specificity	0	0	0	0
Consistency	NA	NA	NA	NA
**Total groups^c^**	**58**		**31**	

^a^ One cross-sectional study studied human participants and one studied monkeys.

^b^ A group of items could contribute to more than one causality dimension, so totals do not sum to 100%.

^c^ Two items contribute to both topics.

Abbreviations: NA, not applicable; evidence about analogous conditions was not searched systematically; the dimension of consistency used information in items included for all other causality dimensions.

### Congenital Brain Abnormalities

A total of 72 items belonging to 58 groups addressed questions related to congenital brain abnormalities up to May 30, 2016 [13, 21–93]. Table 2 summarises the characteristics of 278 mother–infant pairs described in included studies.

**Table 2:** Geographic, clinical, and microbiological characteristics of mother–infant pairs.

Characteristic	References	No. with characteristic^a^	No. evaluated in the article^a^	%^b^
**Total with congenital abnormalities or adverse pregnancy outcomes**		278	278	100
**Country of infection**				
Brazil	[32, 35, 36]	242	278	87.1
Cabo Verde	[13, 66]	2	278	0.7
Colombia	[45]	2	278	0.7
French Polynesia	[51]	19	278	6.8
Martinique	[67]	1	278	0.4
Panama	[55–57]	4	278	1.4
Travellers returning from the Americas	[34, 40, 52, 85]	8	278	2.9
**Pregnancy outcome**				
Miscarriage	[36, 40, 42, 79]	7	278	2.5
Intrauterine death or stillbirth	[38, 42]	3	278	1.1
Termination of pregnancy	[34, 40, 42, 51, 52]	15	278	5.4
Neonatal death	[36, 51, 55–57, 61, 79]	9	278	3.2
Alive, still in utero	[13, 32, 35, 37, 39, 40, 42, 51, 55–57, 66–68, 80, 85, 88–90]	8	278	2.9
Live birth	[13, 32, 35, 37, 39, 40, 42, 51, 55–57, 66–68, 80, 85, 88–90]	236	278	84.9
**Time point of presumed exposure (symptoms)**				
**1st trimester**	[32, 34–37, 39, 40, 42, 51, 52, 79, 85, 88–90]	81	117	69.2
**2nd trimester**	[32, 35, 37, 42, 80, 88]	28	117	23.9
**3rd trimester**	[35, 42, 79, 88]	8	117	6.8
**Exposure assessment in the mother**				
Zika virus (ZIKV)-related clinical symptoms	[32, 34–40, 42, 45, 51, 52, 55–57, 68, 79, 80, 85, 88–90]	180	265	67.9
ZIKV positive in any test (serology/PCR/IHC)	[13, 34, 37, 38, 40, 42, 45, 51, 52, 55–57, 66, 67, 80, 85, 90]	36	41	87.8
ZIKV positive in any test before the outcome	[13, 34, 37, 38, 40, 42, 45, 51, 52, 55–57, 66, 67, 80, 85, 90]	19	36	52.8
ZIKV IgM positive (serum)	[13, 34, 37, 52, 66, 85, 90]	3	7	42.9
ZIKV IgG positive (serum)	[13, 34, 52, 66]	3	3	100.0
ZIKV PRNT positive (serum)	[34, 52, 85, 90]	4	4	100.0
**ZIKV RT-PCR positive (serum)**	[37, 45, 52, 80, 90]	3	7	42.9
ZIKV RT-PCR positive (urine)	[37, 52, 55–57, 90]	1	5	20.0
ZIKV RT-PCR positive (amniotic fluid)	[37, 38, 51, 52, 67]	9	12	75.0
DENV IgG positive	[34, 37, 39, 42, 45, 51, 52, 80, 90]	17	28	60.7
**Exposure assessment in the foetus/newborn**				
ZIKV positive in any test (serology/PCR/IHC)	[13, 34, 36, 38, 40, 52, 61, 66, 68, 79, 80, 85, 88, 90]	74	75	97.4
ZIKV IgM positive (serum)	[68, 80, 85, 90]	30	34	88.2
ZIKV IgG positive (serum)	[13, 34, 66, 80, 90]	4	4	100.0
ZIKV PRNT positive (serum)	[85, 90]	2	2	100.0
**ZIKV RT-PCR positive (serum)**	[61, 67, 68, 90]	2	34	5.9
ZIKV RT-PCR positive (brain tissue)	[34, 36, 38, 52, 79]	6	6	100.0
ZIKV RT-PCR positive (other tissue)	[34, 36, 38, 40, 52, 55–57, 61, 80]	6	11	54.5
ZIKV RT-PCR positive (placenta/product of conception)	[36, 40, 52, 79, 85]	7	8	87.5
ZIKV RT-PCR positive (CSF)	[38, 85, 88]	26	26	100.0
ZIKV IHC positive (brain)	[34, 36, 52, 79]	4	5	80.0
ZIKV IHC positive (other tissue)	[34, 36, 40, 79]	2	7	28.6
ZIKV IHC positive (placenta/product of conception)	[40, 79, 85]	3	4	75.0
DENV IgG positive	[68, 90]	1	34	2.9
**Outcome assessment**				
Clinical microcephaly	[13, 32, 34–40, 42, 51, 55–57, 61, 66–68, 79, 80, 85, 88–90]	244	267	91.4
Imaging confirmed brain abnormalities	[32, 34, 37–40, 42, 45, 51, 52, 55–57, 68, 80, 85, 88–90]	205	213	96.2
Intrauterine growth restriction	[34, 38, 39, 42, 51, 85]	10	35	28.6
Ocular disorders	[35, 37, 40, 42, 51, 68, 80, 85, 88, 89]	49	116	42.2
Auditory disorders	[51, 68]	3	24	12.5
Abnormal amniotic fluid	[42, 51, 61, 80]	6	33	18.2

^a^ The denominator for each characteristic is the number of cases for which data were available.

^b^ Column percentages shown for country of infection, pregnancy outcome, and time point of exposure; row percentages for all other variables.

Abbreviations: CSF, cerebrospinal fluid; DENV dengue virus; IHC, immunohistochemistry; Ig, immunoglobulin; PRNT, plaque reduction neutralisation test; RT-PCR, reverse transcriptase PCR; ZIKV, Zika virus.

Table 3 summarises the assessment for each causality dimension, S4 Table provides an extended description of study findings, and S5 Table summarises the quality of the body of evidence.

**Table 3:** Summary of reviewers’ assessments of evidence about Zika virus infection and congenital abnormalities, by causality dimension.

Causality dimension^a^	Number of items and groups^b^	Evidence summary^c^
**Temporality**	35 items in 21 groups	Reviewer assessments found sufficient evidence for all three questions of an appropriate temporal relationship between Zika virus (ZIKV) infection and the occurrence of congenital abnormalities, including microcephaly. The period of exposure to ZIKV was most likely to be in the first or early second trimester of pregnancy.
**Biological plausibility**	28 items in 25 groups	Reviewer assessments found sufficient evidence for six of seven questions that address biologically plausible mechanisms by which ZIKV could cause congenital abnormalities.
**Strength of association**	7 items in 3 groups	Reviewer assessments found sufficient evidence of a strong association between ZIKV infection and congenital abnormalities for two of two questions. At the population level, there is strong evidence of an association. At the individual level, the effect size was extremely high, although imprecise, in one study and is likely to be high in the other study when follow-up is complete. A newly published case-control study from Brazil shows an effect size similar to that of the retrospective study from French Polynesia.
**Exclusion of alternative explanations**	28 items in 18 groups	Reviewer assessments found sufficient evidence at the individual level that alternative explanations have been excluded for three of seven questions; no other single explanation could have accounted for clusters of congenital abnormalities. The evidence about other exposures could not be assessed because of an absence of relevant studies.
**Cessation**	6 items in 2 groups	Reviewer assessments found sufficient evidence for one of three questions. In two states of Brazil and in French Polynesia, cases of congenital abnormalities decreased after ZIKV transmission ceased. Evidence for the other questions could not be assessed because no relevant studies were identified.
**Dose–response relationship**	0 items	This dimension could not be assessed because of an absence of relevant studies.
**Animal experiments**	20 items in 20 groups	Reviewers assessments found evidence from animal experimental studies for all four questions that supports a causal link between ZIKV and congenital abnormalities. Inoculation with ZIKV of pregnant rhesus macaques and mice can result in foetal abnormalities, viraemia, and brain abnormalities. Experiments to induce viral replication after inoculation of ZIKV intracerebrally and at other sites in a variety of animal models have produced mixed results.
**Analogy**	Not reported	Selected studies reviewed. There are analogies with the well-described group of TORCH infections. Microcephaly has been described following the flavivirus West Nile virus (WNV) infection in pregnancy but not DENV. Evidence was not reviewed systematically.
**Specificity**	0 items	We did not find any studies that identified congenital abnormalities that were found following Zika virus infection in pregnancy but not in other congenital infections. The studies included described a wide range of abnormalities on clinical and neuroimaging examinations. Many of the abnormalities described are also found in other congenital infections, but with a different pattern.
**Consistency**	Not reported	For three of four questions, the evidence assessed was consistent. By geographical region, maternal exposure to ZIKV has been associated with the occurrence of congenital abnormalities in three regions. By study design, the association between ZIKV infection and congenital abnormalities has been found in studies at both individual and population levels and with both retrospective and prospective designs. By population group, ZIKV infection has been linked to congenital abnormalities in both women resident in affected countries and in women from nonaffected countries whose only possible exposure to ZIKV was having travelled in early pregnancy to an affected country. The evidence according to ZIKV lineage is inconsistent because an association between ZIKV and congenital abnormalities has only been reported from countries with ZIKV of the Asian lineage since 2013.

^a^ Questions for each causality dimension are in S2 Table.

^b^ Number of items not reported for Analogy because evidence was not searched for systematically and for Consistency because the evidence about this dimension draws on items that contribute to all other dimensions.

^c^ The complete evidence table is in S4 Table.

Abbreviations: DENV, dengue virus; TORCH, Toxoplasmosis, Rubella, Cytomegalovirus, Herpes simplex virus; WNV, West Nile virus; ZIKV, Zika virus.

#### Temporality

Thirty-five items [30–37, 39–43, 45–47, 49, 51, 52, 57, 60, 65, 67, 68, 73–76, 78, 80, 85, 86, 88–90] in 21 groups addressed temporality (S4 Table). Overall, 67.9% (180/265) of women reported symptoms of Zika virus infection during pregnancy (Table 2). Confirmed infection preceded a diagnosis of microcephaly in a small proportion of cases because many reports were published before laboratory confirmation testing was available. Of the 36 mothers with laboratory-confirmed Zika virus infection, 19 (52.8%) were diagnosed before the detection of foetal malformations or miscarriage [40, 42, 45, 52, 67]. Two detailed case reports show timelines of recent infection followed by neuroimaging evidence of brain abnormalities and subsequent birth with microcephaly [42] or foetal infection [52]. The most likely time point of exposure was the first or the early second trimester, based on individual case reports and three modelling studies [47, 49, 60]. At the population level, epidemic curves of reported Zika virus illness increased in parallel with reported cases of microcephaly, with a lapse of 30 to 34 wk in two states of Brazil (Pernambuco and Bahia) (S2 Text) [57, 60].

#### Biological plausibility

Twenty-eight items [29, 30, 34, 36–38, 40, 41, 44, 51, 52, 54–58, 61, 63, 64, 67, 69, 72, 75, 77, 79, 81, 85, 91–93] in 25 groups addressed biological plausibility (S4 Table). These studies suggest a teratogenic effect of Zika virus on the developing brain. Detailed investigations about a woman with Zika virus infection whose pregnancy was terminated found that isolated viral particles from the foetal brain, but not other tissues, were capable of replication in cell culture [52]. Zika virus RNA was also found in foetal brain tissue in three other studies [34, 36, 38]. Zika virus from both the African and the Brazilian (Asian) lineages replicates in different types of neural progenitor cells (NPCs) [44, 69, 91]. The phosphatidylserine-sensing receptor tyrosine kinase AXL is a potential entry point into human cells and is expressed in developing human cerebral cortex tissue [29, 64]. In vitro studies using NPCs and cerebral organoids show that Zika virus replicates in neural tissue and can disturb the cell cycle and lead to apoptosis [44, 69, 77, 81, 91].

#### Strength of association

We reviewed seven items [42, 46, 47, 49, 60, 78, 86] in three groups up to May 30, 2016 (S4 Table). Two studies suggest that the association between Zika virus infection in pregnancy and congenital brain abnormalities is likely to be very strong [42, 47]. In Rio de Janeiro, investigators compared 72 women with positive reverse transcriptase PCR (RT-PCR) results for Zika virus with 16 women with other causes of rash [42]. Follow-up was more intensive in women with Zika virus infection than those without. Of 42 Zika-infected women with one or more ultrasound scans, 12 (29%) had abnormal scans. All 16 women without Zika virus infection were reported to have had one normal routine scan, but no follow-up data were reported. The preliminary description of the data suggests a marked increase in the risk of congenital abnormalities. In French Polynesia, investigators reconstructed a hypothetical cohort of pregnant women from different sources of data, including eight retrospectively identified cases of microcephaly [47]. They estimated that the risk of microcephaly would be 53.4 times (95% confidence interval 6.5–1,061.2) higher in women with Zika virus infection than in uninfected women if exposure had occurred in the first trimester. Methods and assumptions were clearly described, but the estimate was imprecise and was obtained from indirect data sources. One case-control study in Pernambuco, Brazil, was ongoing at the time of the first searches. The Microcephaly Epidemiology Research Group enrolled 32 cases and 62 controls and found a crude odds ratio of 55.0, 95% CI 8.66–∞) between neonatal microcephaly and laboratory-confirmed Zika virus infection in pregnancy [94].

At population level, state-level data in Brazil showed a positive correlation between case reports of Zika-like illness and cases of microcephaly [49]. These data also show a higher prevalence of microcephaly in 15 states that had reported cases (2.8 per 10,000 live births) than in four states with no reported cases (0.6 per 10,000 live births) [46] (prevalence ratio 4.7, 95% CI 1.9–13.3).

#### Exclusion of alternative explanations

Twenty-eight items [30–39, 41–43, 45, 51, 52, 65, 68, 73–76, 79, 80, 85, 88–90] in 18 groups addressed alternative explanations (S4 Table). No alternative single infectious cause could have resulted in large clusters of cases of microcephaly in different places. Acute dengue virus infection was excluded in 12 studies. Four studies excluded maternal exposure to alcohol or medication, or genetic causes of congenital abnormalities [34, 35, 37, 51]. No study excluded exposure to environmental toxins or heavy metals.

#### Cessation

Six items [46, 49, 57, 60, 78, 86] in two groups addressed this dimension (S4 Table). Surveillance reports of Zika-like illness in northeastern Brazil in 2015 declined [57, 60] either due to seasonality of the vector or population immunity. Reports of microcephaly declined with a similar pattern in Bahia state [60]. In Pernambuco state, a similar pattern was observed but a dengue epidemic occurred simultaneously, so the decline in microcephaly cases might not be attributable to the Zika virus outbreak alone (S2 Text) [57]. We did not find any data on trends in microcephaly cases in countries other than Brazil.

#### Dose–response relationship

We did not find any relevant studies.

#### Experiments in animals

We reviewed 20 items [21–28, 48, 50, 53, 59, 62, 69–71, 82–84, 87] (S4 Table). Studies in the 1950s–1970s show that experimental inoculation of Zika virus resulted in illness, cerebral lesions, and viral replication in the brain in some but not all species tested [21–25, 27, 28]. Some effects might have been enhanced by the numerous serial passaging and subsequent viral adaptation of the original Ugandan Zika strain MR766 and the choice of genetically susceptible animal models. More recent animal studies have shown evidence of neurotropism in immunocompromised mice and in foetal or infant (suckling) immunocompetent mice [48, 71, 82] but not in adult immunocompetent mice [50, 53]. Real-time reports are documenting studies of Macaque monkeys experimentally infected with a Brazilian strain and a French Polynesian strain of Zika virus (both Asian lineage) during pregnancy [70]. High and persisting viraemia was observed in one animal. Foetal autopsy revealed viral RNA in some tissues, but the brain tissue was negative for Zika virus and showed no histopathological lesions or clinical microcephaly.

#### Analogy

Clinical observations linking clusters of babies born with microcephaly and an earlier outbreak of Zika virus infection in Brazil are analogous to the discovery in 1941 of congenital rubella syndrome [95]. Cytomegalovirus and toxoplasmosis in pregnancy can both cause microcephaly, intracranial calcification, and ocular and auditory defects [96] (cited in [43]). Two cases of microcephaly were reported amongst 72 women infected with the neurotropic flavivirus West Nile virus infection in pregnancy [97]. A review of 30 studies of dengue virus infection in pregnancy found evidence of vertical transmission but did not mention microcephaly or other congenital brain abnormalities as possible complications [98].

#### Specificity of association

We did not find any studies that described neuroimaging or clinical features found only in association with Zika virus infection.

#### Consistency

Findings that support Zika virus infection as a cause of congenital brain abnormalities have come from different kinds of epidemiological studies and laboratory studies in both humans and animals (S4 Table). Case reports of pregnancies affected by Zika virus have come from the Americas, the Pacific region (Table 2), and West Africa [13, 66]. The prevalence of microcephaly has not been higher than expected in all countries with Zika virus transmission, however. Congenital brain abnormalities or the presence of Zika virus in products of conception has also been found in pregnant travellers returning from Zika-affected countries [34, 40, 52], showing consistency across populations. There have been no reports of congenital brain abnormalities from countries affected by the African lineage [99]. One in vitro study found that Brazilian (Asian lineage) and African Zika strains both replicated in murine and human cell cultures and organoids [69, 77].

#### Summary of quality of evidence

The body of evidence includes a wide range of study designs and populations in both humans and animals (S5 Table). Much of the evidence in humans comes from uncontrolled or ecological study designs that have inherent biases for ascertaining causal associations. Of two studies that quantified the strength of association, effect sizes were very large but also imprecise [47, 94]. One of three comparative studies was at low risk of bias [94]. Evidence from animal studies is, by its nature, indirect. We could not formally assess publication bias; our search strategy was wide, but we found very few studies with findings that were not consistent with causality.

### Guillain–Barré Syndrome

We found 36 items belonging to 31 groups that addressed questions related to GBS [54–57, 67, 78, 100–122]. We summarise the findings according to clinical characteristics of 118 individuals diagnosed with GBS in Table 4.

**Table 4:** Geographic, clinical, and microbiological characteristics of people with GBS.

** **	**References**	**No. with characteristic**	**No. evaluated**	**%**
**Total N of cases with GBS**		118	118	100
**Country of infection**				
Brazil^a^	[105, 117, 118]	44	118	37.3
El Salvador^a^	[108]	22	118	18.6
French Polynesia	[112]	42	118	35.6
Haiti	[119]	1	118	0.8
Martinique	[113]	2	118	1.7
Panama	[106, 108]	2	118	1.7
Puerto Rico	[110]	1	118	0.8
Travellers returning from the Americas	[108, 120]	3	118	2.5
Venezuela	[111]	1	118	0.8
**Exposure assessment**				
Zika virus (ZIKV) symptomatic cases	[105, 106, 108, 110–112, 117, 118, 120]	84	113	74.3
ZIKV positive in any test (serology/RT-PCR)	[106, 108, 110–113, 117–120]	54	54	100.0
ZIKV IgM positive (serum)	[110, 112, 119]	41	44	93.2
ZIKV IgG positive (serum)	[112]	29	42	69.0
ZIKV PRNT positive (serum)	[112, 119]	43	43	100.0
**ZIKV RT-PCR positive (serum)**	[106, 108, 110, 112, 113, 118, 120]	4	50	8.0
ZIKV RT-PCR positive (urine)	[106, 108, 110, 113, 120]	6	7	85.7
**ZIKV RT-PCR positive (saliva)**		0	0	-
ZIKV RT-PCR positive (CSF)	[106, 108, 113, 118]	2	4	50.0
ZIKV culture positive (serum)		0	0	-
ZIKV culture positive (CSF)		0	0	
DENV IgG positive	[110, 112, 113, 120]	43	45	95.6
**Interval between ZIKV and GBS symptoms, days**		Median 10, range 3–12 [106, 108, 110, 111, 117, 120]French Polynesia: Median 6 (IQR 4–10) [112]El Salvador: 7–15 [108]		

^a^ Only one patient with GBS in Brazil and none in El Salvador had laboratory confirmation of Zika virus infection.

Abbreviations: CSF, cerebrospinal fluid; DENV dengue virus; IQR, interquartile range; Ig, immunoglobulin; PRNT, plaque reduction neutralisation test; RT-PCR, reverse transcriptase PCR; ZIKV, Zika virus.

Table 5 summarises the reviewers’ assessments by causality dimension, S6 Table provides an extended description of study findings, and S7 Table summarises the quality of the body of evidence.

**Table 5:** Summary of reviewers’ assessments of evidence about Zika virus infection and GBS, by causality dimension.

Causality dimension^a^	Number of items and groups^b^	Evidence summary^c^
**1. Temporality**	31 studies in 26 groups	Reviewer assessments found sufficient evidence for all three questions of an appropriate temporal relationship between ZIKV infection and GBS. The time interval between ZIKV symptoms and onset of neurological symptoms was compatible with that of other accepted triggers of GBS.
**2. Biological plausibility**	6 items in 4 groups	Reviewer assessments found sufficient evidence for two of three questions about biologically plausible mechanisms by which ZIKV could trigger the immune-mediated pathology of GBS. There is evidence that supports a role for molecular mimicry, a proposed mechanism of autoimmunity, which has been reported in *C*. *jejuni*-associated GBS. Direct neurotropic effects of ZIKV might also occur.
**3. Strength of association**	7 items in 2 groups	The reviewers assessed evidence from the ZIKV outbreak in French Polynesia as showing a strong association between ZIKV and GBS at both the individual and population levels. Surveillance reports from Brazil also support an association at the population level. Preliminary results from a case-control study in Brazil suggest a similar, strong effect.
**4. Exclusion of alternative explanations**	10 items in 7 groups	Reviewer assessments found sufficient evidence at the individual level that other infectious triggers of GBS have been excluded; no other single infection could have accounted for clusters of GBS. The evidence about other exposures could not be assessed because of an absence of relevant studies.
**5. Cessation**	8 items in 6 groups	Reviewer assessments found sufficient evidence for one of three questions. In one state in Brazil, four other countries in the Americas, and in French Polynesia, reports of GBS decreased after ZIKV transmission ceased. Evidence for the other questions could not be assessed because no relevant studies were identified.
**6. Dose–response relationship**	0 items	No relevant studies identified.
**7. Animal experiments**	0 items	No relevant studies of animal models of immune-mediated neuropathology identified. Evidence about neurotropism of ZIKV summarised in S4 Table.
**8. Analogy**	Not reported	Evidence was not reviewed systematically; selected studies reviewed for two of three questions. Analogous mosquito-borne neurotropic flavivirus infections have been reported in association with GBS (WNV; DENV; JEV). WNV and JEV have also been reported to be associated with direct neurotropic effects and poliomyelitis-like acute flaccid paralysis. The time lag between ZIKV symptoms and GBS symptoms is analogous to intervals reported for other infectious triggers of GBS. There is some evidence that, as for *C*. *jejuni*-associated GBS, molecular mimicry could be involved.
**9. Specificity**	0 items	No relevant studies identified.
**10. Consistency**	Not reported	For three of four questions, there was sufficient evidence of consistency. By geographical region, ZIKV transmission has been associated with the occurrence of GBS in two of three regions where ZIKV has circulated since 2007. By study design, the association between ZIKV infection and GBS has been found in studies at both individual and population levels. By population group, ZIKV infection has been linked to GBS in both residents of an affected country and travellers from nonaffected countries whose only possible exposure to ZIKV was having travelled to an affected country. The evidence according to ZIKV lineage is unclear because an association between ZIKV and GBS has only been reported from countries with ZIKV of the Asian lineage since 2013.

^a^ Questions for each causality dimension are in S2 Table.

^b^ Number of items not reported for dimension 8 (Analogy), because evidence was not searched for systematically, and for Consistency, because the evidence about this dimension draws on items that contribute to all other dimensions.

^c^ The complete evidence table is in S6 Table

Abbreviations: DENV, dengue virus; GBS, Guillain–Barré syndrome; JEV, Japanese encephalitis virus; WNV, West Nile virus; ZIKV, Zika virus.

#### Temporality

We included 31 items [55–57, 67, 78, 100–112, 115–117, 120–122] in 26 groups (S6 Table). At the individual level, symptoms of Zika virus infection were reported before the onset of GBS symptoms in cases in French Polynesia, Brazil, El Salvador, Panama, Puerto Rico, and Venezuela, and in returning travellers from Haiti, Suriname, and Central America. All patients with GBS had laboratory-confirmed Zika virus infection except for 42 of 44 in Brazil and all those in El Salvador. The intervals between Zika virus infection and neurological symptoms delays of 3 to 12 d [108, 111, 112] are consistent with a postinfectious autoimmune mechanism [5]. In one ecological study in Bahia, Brazil, a lag of 5 to 9 wk between the epidemic peaks of cases with acute exanthematous illness and GBS was attributed to data collection issues [78].

At the population level, 11 countries in Latin America (Brazil, Colombia, El Salvador, French Guiana, Honduras, Venezuela, Suriname) and the Caribbean (Dominican Republic, Jamaica, Martinique) and French Polynesia have reported an increase in GBS cases during outbreaks of Zika virus infection [56, 57, 67, 104, 107, 109]. Surveillance reports show sporadic GBS cases in association with Zika-like illness in four countries but without an increase above background level (Guadeloupe, Haiti, Panama, Puerto Rico). One study reported on surveillance data about acute flaccid paralysis in children in 20 South Pacific islands. The number of expected cases of acute flaccid paralysis was <1 per year in these small countries, and an increase during periods of Zika virus transmission was only observed in the Solomon Islands [115].

#### Biological plausibility

We reviewed six items [54, 102, 111, 112, 114, 116] in four groups (S6 Table). Anti-ganglioside antibodies, whose presence supports the clinical diagnosis of GBS, were found in the serum of a third of patients in a case-control study in French Polynesia [112] and in one patient from Venezuela [111]. The case-control study and two in silico studies also provide some evidence for molecular mimicry of Zika virus epitopes and host antigens [112]. Studies of predicted epitopes and human antigens suggested peptide sharing between Zika virus and human proteins [54, 114]. Several experimental studies with human neural stem cells and mouse models have shown some evidence for neurotropism of Zika virus (S4 Table, biological plausibility).

#### Strength of association

We reviewed seven items [101–104, 112, 116, 122] in two groups identified up to May 30, 2016. One published case-control study enrolled 42 cases of GBS during the Zika outbreak in French Polynesia and compared them with 98 patients hospitalised with nonfebrile illness (S6 Table) [112]. Several alternative causes of GBS were excluded. Evidence of Zika virus infection was much more common in GBS cases than controls (odds ratios 59.7, 95% CI 10.4–+∞ defined as IgM or IgG positivity and 34.1, 95% CI 5.8–+∞ defined as presence of neutralising antibodies). Cases and controls were matched, but there was no additional adjustment for confounding. In Brazil, surveillance data showed a 19% increase in reports of GBS cases in 2015 compared with 2014 [101]. Information received after May 30 found a second case-control investigation conducted in Brazil that enrolled controls from the community and is ongoing; preliminary results suggest a similar, strong effect (Sejvar J., personal communication).

#### Alternative explanations

We included ten items [102, 110, 112, 113, 116–121] in seven groups (S6 Table). In several studies, other infections that can trigger GBS were excluded, such as *C*. *jejuni*, *Mycoplasma pneumoniae*, HIV, Epstein–Barr virus, and herpes simplex virus. No single infectious trigger would have resulted in GBS outbreaks in multiple locations.

#### Cessation

Eight items [56, 57, 78, 103, 104, 109, 122] in six groups addressed cessation (S6 Table). In surveillance reports from six countries (Brazil, Colombia, El Salvador, French Polynesia, Honduras, and Suriname), the incidence of GBS declined as reports of Zika virus infection fell.

#### Dose–response relationship, experiments in animals, and specificity

We did not find any relevant studies.

#### Analogy

Clusters of cases of GBS have been reported in association with outbreaks of *C*. *jejuni* gastroenteritis [123]. The incidence of GBS estimated from studies in French Polynesia of 0.24 per 1,000 Zika virus infections [112] is at the lower end of estimates for *C*. *jejuni* (0.3 [124] and 1.17 [125] per 1,000). The reported latency between gastrointestinal symptoms and onset of paralysis of approximately 9 d (range 1–23 d) [124, 126, 127] is similar to Zika virus-associated cases. Other mosquito-borne neurotropic flaviviruses have been reported as possible triggers of GBS in case reports and case series: dengue virus [128], West Nile virus [129], Japanese B encephalitis virus [130, 131], or yellow fever 17D vaccination [132]. An acute poliomyelitis-like flaccid paralysis, resulting from direct neural infection, presumably of anterior horn cells, has also been reported as a clinical consequence of these viruses [129, 133, 134]. Putative biological mechanisms include up-regulation of major histocompatibility class I and II molecules of peripheral nerve cells and subsequent immune-mediated cell destruction [135], auto-antibodies directed against heat shock proteins [136], galactocerebrosides [137], or myelin basic protein (MBP), and proliferation of MBP-specific T-cells [138].

#### Consistency

The link between Zika virus and GBS has been made in studies of different designs at individual and population levels (S6 Table). Clusters of GBS have been seen in multiple countries during epidemics of Zika virus but have not been reported in all those in which Zika virus outbreaks have occurred. Outbreaks of GBS in which gene sequencing has been done were associated with Zika virus of the Asian lineage.

#### Summary of quality of evidence

The body of evidence includes a wide range of study designs and populations in humans (S7 Table). A majority of the evidence reviewed was from uncontrolled or ecological study designs that have inherent biases for ascertaining causal associations. The only study that examined the strength of association found a very large but imprecise estimate of the effect size but did not have serious risks of bias [112]. There was no evidence of indirectness. We could not formally assess publication bias, but we had a broad search strategy, and we did find evidence that outbreaks of GBS have not been seen in all countries with Zika virus transmission.

#### Cofactors that might act in the presence of Zika virus

We prespecified seven categories of cofactors (S2 Table). The most widely discussed was past dengue virus infection [112]. A mechanism known as antibody-dependent enhancement might be involved, when IgG antibodies against viral envelope proteins resulting from a prior infection bind to virus particles of a subsequent infection, leading to enhanced replication and potentially more severe illness [139]. Evidence from in vitro experiments suggests cross-reactivity between dengue and Zika virus antibody responses and antibody-dependent enhancement of Zika virus by dengue antibodies [139, 140]. In several of the studies that we reviewed, evidence of past dengue virus infection was reported (S1 Text, p4-5). We did not systematically review evidence for other cofactors but report additional narrative findings in S1 Text (p4-5).

### WHO Expert Panel Conclusions

Based on the evidence identified up to July 29, 2016, the expert panel concluded that

- the most likely explanation of available evidence from outbreaks of Zika virus infection and clusters of microcephaly is that Zika virus infection during pregnancy is a cause of congenital brain abnormalities including microcephaly and
- the most likely explanation of available evidence from outbreaks of Zika virus infection and GBS is that Zika virus infection is a trigger of GBS [141].

The expert panel recognises that Zika virus alone may not be sufficient to cause either congenital brain abnormalities or GBS. The panel does not know whether these effects depend on as yet uncharacterised cofactors being present, nor does it know whether dengue virus plays a part, as this is carried by the same species of mosquito and has circulated in many countries during the same period.

## Discussion

Up to May 30, 2016, we found evidence that supported a causal association between Zika virus infection and congenital brain abnormalities, including microcephaly, with at least one study addressing one or more specific questions for eight of ten causality dimensions and between Zika virus infection and GBS, with at least one study about one or more specific questions in seven of ten dimensions. There are methodological weaknesses, inconsistencies, and gaps in the body of evidence for both sets of conditions. Studies found after the cut-off for our first searches did not change our conclusions but strengthened the evidence about biological plausibility, strength of association, and exclusion of alternative explanations.

### Interpretation of the Review Findings

The expert panel’s conclusions support causal links between Zika virus and congenital brain abnormalities and GBS and address Bradford Hill’s question, “…is there any other answer equally, or more, likely than cause and effect?” [7]. The conclusions consider both the epidemiological context of unexpected clusters of different types of neurological conditions in countries that have experienced their first outbreaks of Zika virus infection and the strengths and weaknesses of a systematically reviewed body of evidence about ten dimensions of causality (S4 Table, S5 Table, S6 Table and S7 Table). Empirical observations cannot “prove” causality, however [7, 142], and discussions have been intense [9]. A cause can be identified without understanding all the necessary component causes or the complete causal mechanisms involved [142, 143]. In the case of GBS, the infections that precede it are often referred to as “triggers” of the immune-mediated causal pathways involved in pathogenesis.

The body of evidence about Zika virus and congenital abnormalities (72 items included) has grown more quickly than that for GBS (36 items). Research efforts might have concentrated on congenital abnormalities because clusters of affected infants were so unusual, especially in Brazil, where rubella has been eradicated. In contrast, GBS is an established postinfectious neurological disorder, and some commentators have already assumed Zika virus as another cause [12]. Whilst only one case-control study from French Polynesia has been published so far [112], clusters of GBS during outbreaks of Zika virus infection have been reported from several other countries [144], and case-control studies are ongoing in Brazil, Colombia, Mexico, and Argentina.

Comparative studies from French Polynesia suggest that the risk of both microcephaly or of GBS is at least 30 times higher in people who had Zika virus infection compared to those who did not [47, 94, 112], although confidence intervals are wide. The true effect size might be weaker because the earliest studies investigating causality are often overestimates [145]. Even if the methods of forthcoming studies in Brazil [42] and elsewhere reduce confounding and bias, the increase in the risk of disease amongst those with Zika virus infection is likely to remain substantially raised. Inconsistencies in the evidence still need investigation, however. Disease clusters were not seen in Africa [146], but congenital abnormalities and GBS are rare complications that might not be detected in countries with small populations or poor surveillance systems. In the case of microcephaly, terminations of potentially affected pregnancies might have resulted in underascertainment [147].

Current evidence does not show which specific environmental and host factors interact with Zika virus. A cofactor that increases the risk of neurological damage could help to explain why surveillance reports show clusters of microcephaly or GBS in some geographical areas but not others. Dengue virus has been suggested as a possible cofactor or another component cause [143]. One major limitation to interpretation of data about causality and cofactors is the lack of accurate and accessible diagnostic tools, owing to the short duration of viraemia, cross-reactivity with other flaviviruses, and lack of standardisation [148].

### Strengths and Limitations

The strengths of our study are that we appraised evidence of causality systematically but rapidly and transparently within a structured framework. We searched both published and unpublished sources. The systematic review process could not eliminate publication bias but reduced the risk that only positive reports in favour of causation would be evaluated. There were limitations to the process, too. Our search strategy did not cover the literature about analogous conditions or cofactors systematically. We did not select studies or extract data in duplicate, but additional reviewers checked the extracted data independently. The included studies used a variety of case definitions for microcephaly and GBS, and we could not standardise these, so misclassification is possible, but this limitation did not change the overall conclusions. Our rapid assessment of quality was not quantitative; we did not find a tool that covered all review questions and study designs appropriately and were not able to standardise the GRADE tool across study designs in the time available [20].

### Implications for Policy and Research

The conclusions of the expert panel facilitate the promotion of stronger public health measures and research to tackle Zika virus and its effects. The evidence gaps that we identified provide researchers with research questions, and WHO has published a Zika virus research agenda [149]. Better diagnostic tests will allow more accurate assessment of Zika virus in tissues and of population-level immunity. Research about Zika virus and acute flaccid paralysis is needed to define the clinical and electrophysiological pattern, mechanisms of causality, and to distinguish between the roles of autoimmunity and direct effects on anterior horn cells or neurons. Basic research will also further the development of vaccines, treatments, and vector control methods. For the populations currently at risk, cohort studies are needed to determine both absolute and relative risks of pregnancies affected by asymptomatic and symptomatic Zika virus infection and the role of cofactors and effect modifiers, and to define the full range of physical and developmental abnormalities that comprise the congenital Zika virus syndrome.

### From Rapid Systematic Review to Living Systematic Review

Our systematic review deals with multiple neurological disorders and more detailed questions about causality than other reviews. We reached the same conclusion as Rasmussen et al. [11], but the larger number of studies allowed a more comprehensive and balanced summary of evidence and of evidence gaps. In addition, our review addresses the association between Zika virus and GBS. We also plan to examine other acute neurological disorders (S1 Text).

Our review will quickly become outdated because the pace of new publications is outstripping the time taken for the review process. The concept of a “living systematic review” has been proposed as a way to combine rigour with timeliness for intervention research [150] through the development of methods to incorporate new evidence as soon as it is available and make evidence summaries available immediately. We are working on methods to produce a living systematic review of the Zika causality framework that will incorporate new studies, provide frequent open access updates, and allow cumulative meta-analyses of both aggregate and individual patient data from rigorous prospective studies as these become available. The declaration by journal editors to improve access to data during public health emergencies [151, 152] could be combined with the living systematic review approach to improve timeliness and open access to research about causality [153].

In summary, rapid and systematic reviews with frequent updating and open dissemination are now needed, both for appraisal of the evidence about Zika virus infection and for the next public health threats that will emerge. This rapid systematic review found sufficient evidence to conclude that Zika virus is a cause of congenital abnormalities and is a trigger of GBS.

## Supporting Information

S1 PRISMA Checklist (PDF)

S1 Text Background to assessment of causality in epidemiology, Zika causality framework questions, supplementary methods, and results. (PDF)

S2 Text Epidemic curves of reported cases of dengue, chikungunya, Zika virus, and microcephaly in Pernambuco state, Brazil, by epidemiological week, 2015 to 2016. From PAHO Epidemiological Update 28 April 2016 (Fig 7, p7).
(PDF)

S1 Table Bradford Hill’s “viewpoints” of causation, modifications by Gordis, and adaptation to the dimensions of the causality framework, in order. (PDF)

S2 Table Zika causality framework for congenital abnormalities and GBS, according to causality dimension. (PDF)

S3 Table Characteristics of included and excluded items, total 1,091 unique items. (PDF)

S4 Table Causality framework evidence for congenital brain abnormalities. (PDF)

S5 Table Quality assessment of the body of evidence about congenital brain abnormalities. (PDF)

S6 Table Causality framework evidence for GBS. (PDF)

S7 Table Quality assessment of the body of evidence about GBS. (PDF)

S1 Fig Flowchart of included and excluded items. (TIF)

## Abbreviations

GBS: Guillain–Barré syndrome
GRADE: Grading of Research Assessment Development and Evaluation
MBP: myelin basic protein
NPCs: neural progenitor cells
PAHO: Pan American Health Organization
PRISMA: Preferred Reporting Items for Systematic Reviews and Meta-Analyses
RT-PCR: reverse transcriptase PCR
WHO: World Health Organization
