# Supplementary material for: Zika Virus Infection as a Cause of Congenital Brain Abnormalities and Guillain–Barré Syndrome: Systematic Review
Source: PLoS Med. 2017 Jan 3;14(1):e1002203. doi: 10.1371/journal.pmed.1002203 (PMC5207634; doi:10.1371/journal.pmed.1002203)
Supplement: S1 PRISMA Checklist — (PDF) [file pmed.1002203.s001.pdf]

PRISMA 2009 Checklist

Section/topic	#	Checklist item	Reported on page #
**TITLE**			
Title	1	Identify the report as a systematic review, meta-analysis, or both.	p1
**ABSTRACT**			
Structured summary	2	Provide a structured summary including, as applicable: background; objectives; data sources; study eligibility criteria, participants, and interventions; study appraisal and synthesis methods; results; limitations; conclusions and implications of key findings; systematic review registration number.	Abstract
**INTRODUCTION**			
Rationale	3	Describe the rationale for the review in the context of what is already known.	Introduction
Objectives	4	Provide an explicit statement of questions being addressed with reference to participants, interventions, comparisons, outcomes, and study design (PICOS).	Introduction, para 3 Adapted for multiple observational review questions
**METHODS**			
Protocol and registration	5	Indicate if a review protocol exists, if and where it can be accessed (e.g., Web address), and, if available, provide registration information including registration number.	Methods, para 1
Eligibility criteria	6	Specify study characteristics (e.g., PICOS, length of follow-up) and report characteristics (e.g., years considered, language, publication status) used as criteria for eligibility, giving rationale.	Methods, Systematic review, Eligibility and S1 Text p3, para 3
Information sources	7	Describe all information sources (e.g., databases with dates of coverage, contact with study authors to identify additional studies) in the search and date last searched.	Methods, Systematic review, Information sources and search strategy and S1 Text p3, para 5
Search	8	Present full electronic search strategy for at least one database, including any limits used, such that it could be repeated.	Methods, Systematic

			review, Information sources and search strategy and S1 Text p3, para 4
Study selection	9	State the process for selecting studies (i.e., screening, eligibility, included in systematic review, and, if applicable, included in the meta-analysis).	Methods, Systematic review, Study selection and data extraction and S1 Text p3, para 7
Data collection process	10	Describe method of data extraction from reports (e.g., piloted forms, independently, in duplicate) and any processes for obtaining and confirming data from investigators.	Methods, Systematic review, Study selection and data extraction and S1 Text p3, para 7 and p4, para 1
Data items	11	List and define all variables for which data were sought (e.g., PICOS, funding sources) and any assumptions and simplifications made.	See data files
Risk of bias in individual studies	12	Describe methods used for assessing risk of bias of individual studies (including specification of whether this was done at the study or outcome level), and how this information is to be used in any data synthesis.	Methods, Systematic review, Synthesis of findings and S1 Text p4, para 2
Summary measures	13	State the principal summary measures (e.g., risk ratio, difference in means).	Descriptive analysis only
Synthesis of results	14	Describe the methods of handling data and combining results of studies, if done, including measures of consistency (e.g., I^2) for each meta-analysis.	Methods, Systematic review, Synthesis of

PRISMA 2009 Checklist

			findings and S1 Text p4, para 2

Section/topic	#	Checklist item	Reported on page #
Risk of bias across studies	15	Specify any assessment of risk of bias that may affect the cumulative evidence (e.g., publication bias, selective reporting within studies).	Methods, Systematic review, Synthesis of findings and S1 Text p4, para 2
Additional analyses	16	Describe methods of additional analyses (e.g., sensitivity or subgroup analyses, meta-regression), if done, indicating which were pre-specified.	None
**RESULTS**			
Study selection	17	Give numbers of studies screened, assessed for eligibility, and included in the review, with reasons for exclusions at each stage, ideally with a flow diagram.	Results, para 1 and S1 Fig
Study characteristics	18	For each study, present characteristics for which data were extracted (e.g., study size, PICOS, follow-up period) and provide the citations.	Table 1, S3 Table
Risk of bias within studies	19	Present data on risk of bias of each study and, if available, any outcome level assessment (see item 12).	Results, para 15 and 27
Results of individual studies	20	For all outcomes considered (benefits or harms), present, for each study: (a) simple summary data for each intervention group (b) effect estimates and confidence intervals, ideally with a forest plot.	Table 2, S4 Table, Table 4, S6 Table
Synthesis of results	21	Present results of each meta-analysis done, including confidence intervals and measures of consistency.	(descriptive summaries, Table 3, Table 5)
Risk of bias across studies	22	Present results of any assessment of risk of bias across studies (see Item 15).	Results, para 15 and S5 Table; para 27 and S7 Table

Additional analysis	23	Give results of additional analyses, if done (e.g., sensitivity or subgroup analyses, meta-regression [see Item 16]).	None
**DISCUSSION**			
Summary of evidence	24	Summarize the main findings including the strength of evidence for each main outcome; consider their relevance to key groups (e.g., healthcare providers, users, and policy makers).	Results, WHO expert panel conclusions and Discussion, para 1
Limitations	25	Discuss limitations at study and outcome level (e.g., risk of bias), and at review-level (e.g., incomplete retrieval of identified research, reporting bias).	Discussion, para 6
Conclusions	26	Provide a general interpretation of the results in the context of other evidence, and implications for future research.	Discussion, para 2-3, para 7, para 10
**FUNDING**			
Funding	27	Describe sources of funding for the systematic review and other support (e.g., supply of data); role of funders for the systematic review.	Funding disclosure

From: Moher D, Liberati A, Tetzlaff J, Altman DG, The PRISMA Group (2009). Preferred Reporting Items for Systematic Reviews and Meta-Analyses: The PRISMA Statement. PLoS Med 6(6): e1000097. doi:10.1371/journal.pmed1000097

For more information, visit: **www.prisma-statement.org**.
