# Supplementary material for: Zika Virus Infection as a Cause of Congenital Brain Abnormalities and Guillain–Barré Syndrome: Systematic Review
Source: PLoS Med. 2017 Jan 3;14(1):e1002203. doi: 10.1371/journal.pmed.1002203 (PMC5207634; doi:10.1371/journal.pmed.1002203)
Supplement: S1 Text — (PDF) [file pmed.1002203.s002.pdf]

Supplementary text

Zika virus infection as a cause of congenital brain abnormalities and Guillain-Barré syndrome: systematic review

Fabienne Krauer,[1] Maurane Riesen,[1] Ludovic Reveiz,[2] Olufemi T Oladapo,[3] Ruth Martínez-Vega,[4] Teegwendé V. Porgo,[3,5] Anina Haefliger,[1] Nathalie J Broutet, [3] Nicola Low,[1*] for the WHO Zika Causality Working Group[6]

1. Institute of Social and Preventive Medicine, University of Bern, Switzerland
2. Pan American Health Organization, Washington DC, United States of America
3. UNDP/UNFPA/UNICEF/WHO/World Bank Special Programme of Research, Development and Research Training in Human Reproduction (HRP), Department of Reproductive Health and Research World Health Organization, Geneva, Switzerland
4. Escuela de Microbiologia, Universidad Industrial de Santander, Santander, Colombia
5. Department of Social and Preventative Medicine, Laval University, Québec, Canada
6. Membership of the WHO Zika Causality Working Group is provided in the Acknowledgements

Contents

1. Introduction 2
2. Background to methods for the assessment of causal associations in epidemiology 2
3. Development of the causality framework 3
4. Systematic review methods 3
   Eligibility 3
   Information sources and search strategy 3
   Study selection and data extraction 3
   Synthesis of findings and assessment of methodological quality 4
5. Supplementary results 4
   Co-factors 4
6. Acknowledgements 5
7. References 6

S1 Text

1. Introduction

This document describes our conceptual approach to the study of causality and the development of our causality framework for the examination of the observed links between Zika virus infection and neurological conditions, including microcephaly and Guillain-Barré syndrome (sections 2 and 3). We also report essential items about the methods and results of the systematic review that supplement the main text and complete the Preferred Reporting Items for Systematic Reviews and Meta-Analyses guidelines for systematic review protocols (PRISMA-P) [1] and reports (PRISMA) [2] (sections 4 and 5).

The words "cause" and "causal", when applied to the nature of the links between Zika virus infection, have been hotly debated [3]. The debate probably reflects the difficulties in reaching a decision as to when there is enough evidence to make a statement that A is a cause of B because of the severity of the conditions themselves and the implications for control measures and public health. In the course of our project we found that the terminology and sometimes the concepts themselves are not understood in depth and are open to misinterpretation. The determination of causation as an explanation for observed associations is one of the core tasks of public health. Jan Vandenbroucke, a member of the expert panel, offered extensive insights based on his experience of investigating causal relationships [4-6] and his knowledge of others' contributions [7-10]. This expertise helped us to clarify the application of the concepts to our research question.

2. Background to methods for the assessment of causal associations in epidemiology

We used textbooks of epidemiology to list the key aspects of causal associations that we wanted to cover. These aspects are commonly referred to as the "Bradford Hill criteria" and used widely to show how causal inferences are derived from observed links between an exposure and a disease. The term is based on the work of Sir Austin Bradford Hill and his investigations of the association between cigarette smoking and lung cancer [11, 12]. Rothman criticises the use of the word criteria because it encourages a rigid approach, such that people apply the criteria as a set of rules [13]. Bradford Hill used the term "viewpoints" and stated that they were not "hard-and-fast rules of evidence that must be obeyed before we accept cause and effect." [11]. We agree and decided to use the word "dimension" in our causality framework to capture the notion of a multifaceted approach.

We used Leon Gordis' textbook [14] as the starting point for both the content and the order of our list of dimensions. Gordis first presents nine items as "guidelines" modified from the 1960s lists of both the US Surgeon General and Bradford Hill (S1 Table, Gordis I). Bradford Hill starts his list with the assessment of the strength of association [11]. Gordis modified the list and described a modification of the guidelines that gave more weight to some (major criteria) than to others (other considerations) [14]. This modification was part of a US Public Health Service exercise in the 1980s to examine the scientific basis for prenatal care interventions. This modification recognised that causation was a central concept for the assessment of the effects of measures intended to improve health. The US Public Health Service process also incorporated a categorisation of the quality of the evidence. Assessing the quality of evidence is also an integral part of the systematic review process.

S1 Text

3. Development of the causality framework

Our causality framework is based on 10 dimensions of causality (S2 Table). We defined questions for each dimension and each set of clinical outcomes for the first time on 22.02.2016. The framework also specified the types of study design expected to address each question and, for epidemiological study designs, distinguished between evidence that would be available at the level of the individual and at the population level. Colleagues from the WHO Zika Causality Working Group helped us to refine the questions and to incorporate an explicit consideration that there might be co-factors that act in some way with Zika virus to result in or increase the probability of adverse neurological conditions.

4. Systematic review methods

We describe the systematic review methods here in full, including steps that we took to expedite the review process [15].

Eligibility

We included studies of any design and in any language that directly addressed any research question in the causality framework (S1 Text). We excluded reviews, commentaries, news items and journal correspondence that did not include original data but we checked their reference lists to identify other potentially relevant studies.

Information sources and search strategy

The search strategy was designed to find data about Zika virus and its consequences from ongoing studies and non-peer reviewed sources as well as published peer-reviewed studies to benefit from commitments to data-sharing in public health emergencies [16]. The Pubmed search string was: zika [Title/Abstract] OR ZIKV [Title/Abstract] OR zika virus [MeSH Terms] OR zika virus infection [MeSH Terms].

We searched: PubMed, Embase and LILACS electronic databases; PAHO Zika research portal, WHO and the European Centre for Disease Prevention and Control (ECDC) websites; journal websites; preprint servers and a real time updated portal of experimental animal studies [17] (see protocol [18] and S1 Text). We also asked experts of the WHO groups. For the dimension addressing analogous causes of the outcomes and for co-factors, we did not conduct systematic reviews. Instead, we used items identified in the searches, their reference lists and additional non-systematic searches. We used Endnote X7 (Thomson Reuters, Philadelphia) for reference management.

We conducted our first search from the earliest date to April 11, 2016 and updated the search on May 30 and July 29. We selected items and extracted data systematically on included items up to May 30 and report on non-systematically identified studies up to July 29, 2016.

Study selection and data extraction

We used pre-piloted structured forms in the online database Research Electronic Data Capture (REDCap, Vanderbilt University, Nashville). To speed up study selection we screened titles, abstract and full texts by liberal accelerated screening [15]; two reviewers each screened half of the titles and abstracts once. All items that either reviewer thought relevant, or needed more information to assess, were retained for full text screening. Two reviewers had to agree to exclude an item. We applied the same strategy to full text manuscripts.

For data extraction in all studies we recorded publication year, location of authors, funding sources, study design and the causality question addressed. In clinical studies, we extracted data about the

temporal sequence of events, presumed time point of exposure, laboratory tests to assess exposure and outcome, and clinical case definitions and outcomes. A measure of effect (risk ratio, odds ratio, prevalence ratio) was extracted where available or we calculated the effect measure if the authors reported the appropriate data. One reviewer extracted data and a second reviewer checked the extracted data. Discrepancies were resolved by discussion or by a third reviewer. We did not specify a single primary outcome because the number of causality dimensions and questions was too broad [15]. The data to be extracted differed according to the study design and the question(s) addressed. We used case definitions and laboratory diagnostic test interpretations as reported by study authors. Basic research studies were too diverse to allow consistent numerical data extraction so we summarised findings descriptively.

Synthesis of findings and assessment of methodological quality

We tabulated study level data and available data about clinical presentations from case reports, case series, cross-sectional studies, case-control studies and cohort studies. We assessed methodological quality for these designs using shortened checklists from the National Institute of Health and Clinical Excellence [19] and using reviewers' summaries of strengths and weaknesses of other study designs. Each reviewer recorded an overall judgement of each study to indicate whether the findings did or did not provide support for the causality dimension being assessed. Two reviewers reached consensus by discussion or adjudication by a third reviewer. We assigned a judgement of sufficient evidence about a causality dimension if the reviewers' assessments were supportive for at least half of the specific questions. We appraised the body of evidence according to the domains of the Grading of Research Assessment Development and Evaluation (GRADE) tool as suggested for urgent health questions [20], but did not apply upgrading or downgrading because these concepts could not be applied consistently across the range of study designs.

5. Supplementary results

Co-factors

A range of possible contributing factors have been mentioned such as other infections, host or pathogen characteristics or environmental agents. There are genetic differences between the African and Asian lineages of Zika virus, but further research is needed to show whether these differences result in differential virulence.

The role of dengue virus (DENV) antibodies has been discussed most as a co-factor. A potential interaction between Zika virus and DENV antibodies has been raised by several experts. It is suspected that the presence of DENV IgG antibodies could enhance the entry of Zika virus into cells and possibly increase viral replication; this mechanism is called antibody-dependent enhancement (ADE). It is believed that IgG antibodies against viral envelope proteins resulting from a prior infection bind to virus particles of a subsequent infection. The virus-antibody complex reacts then with the Fc receptor on the surface of innate immune cells. The internalisation of this complex via the Fc receptor may lead to increased viral replication by suppression of antiviral response [21, 22]. Other individual-level studies provide some clues. In a cohort study on microcephaly conducted in Brazil, the majority of the women showed DENV IgG at the time point of enrollment, and also a mother returning from Brazil to Slovenia was IgG positive for DENV, yellow fever (YF, through vaccination), West Nile Virus (WNV) and tick-borne encephalitis virus (TBEV). Other mothers did not show seroconversion for dengue. An ecological study found a positive association between the force of infection for dengue and the prevalence of microcephaly in Brazil [23]. In French Polynesia, 41 of 42 GBS patients had DENV IgG antibodies as well as two GBS patients from Martinique. Two

returning travellers with laboratory confirmed Zika infection and dengue IgG antibodies experienced sequelae such as severe thrombocytopenia [24] or a hearing disorder [25]. A recent publication showed binding of human antibodies against DENV to both the African and a French Polynesian strain of Zika virus as well as enhanced infection of a human myeloid cell line with ZIKV upon pre-incubation with DENV antibodies [22]. These findings suggest that prior dengue infection may play a role in the development of sequelae from Zika virus infection, but no appropriate study has been identified yet to answer this question definitively.

The experimental inoculation of a volunteer with Zika virus in the 1950s showed that YF antibodies from prior vaccination may rise after exposure to Zika [26], suggesting some cross-reactivity between these two flaviviruses. A series of animal experiments showed that rhesus monkeys immunised with Zika virus were not protected against a challenge with YF, but Zika virus immunisation in Vervet monkeys reduced YF viraemia [27]. Serological cross-reactivity between flaviviruses has been shown in several laboratory studies; Zika virus antibodies neutralised WNV and DENV, WNV antibodies neutralised Zika virus [28], YF monoclonal antibodies reacted with Zika virus [29] and Zika virus growth in cell culture was increased when antibodies against YF were added [30]. The association between Zika and YF antibodies in a serosurvey in Nigeria has led to the hypothesis that immunity to Zika reduced the severity of YF [31]. One descriptive ecological study examined the spatial distribution of microcephaly and YF vaccination coverage in Brazil and found several clusters of microcephaly in areas with particularly low YF vaccination coverage [32]. However, at the level of the individual, congenital abnormalities were detected in two pregnant women who returned with Zika virus infection from the Americas despite YF immunisation [33, 34]. In summary, the evidence on whether YF immunisation may have a protective or deleterious effect on Zika virus infection is sparse and this question needs more investigation.

One report hypothesised that an insecticide used to treat drinking water (pyriproxyfen) could cause microcephaly due to possible biochemical interactions with growth regulators and observed that microcephaly cases in Brazil were reported after the introduction of the insecticide [35]. This article did not provide any specific data about exposure in affected women and was therefore excluded from the review.

6. Acknowledgements

WHO Zika causality working group members: Nathalie Broutet, Maria Almiron, Tarun Dua, Christopher Dye, Pierre Formenty, Florence Fouque, Metin Gulmezoglu, Edna Kara, Anais Legand, Bernadette Murgue, Susan Norris, Olufemi T Oladapo, William Perea Caro, Pilar Ramon Pardo, Ludovic Reveiz Herault and João Paulo Souza.

7. References

1. Moher D, Shamseer L, Clarke M, Ghersi D, Liberati A, Petticrew M, et al. Preferred reporting items for systematic review and meta-analysis protocols (PRISMA-P) 2015 statement. Syst Rev. 2015;4(1):1. doi: 10.1186/2046-4053-4-1. PubMed PMID: 25554246; PubMed Central PMCID: PMC4320440.

2. Moher D, Liberati A, Tetzlaff J, Altman DG, Group P. Preferred reporting items for systematic reviews and meta-analyses: the PRISMA statement. PLoS Med. 2009;6(7):e1000097. doi: 10.1371/journal.pmed.1000097. PubMed PMID: 19621072; PubMed Central PMCID: PMC2707599.

3. Doshi P. Convicting Zika. BMJ. 2016;353:i1847. Epub 2016/04/09. doi: 10.1136/bmj.i1847. PubMed PMID: 27056643.

4. Vandenbroucke JP, Koster T, Briet E, Reitsma PH, Bertina RM, Rosendaal FR. Increased risk of venous thrombosis in oral-contraceptive users who are carriers of factor V Leiden mutation. Lancet. 1994;344(8935):1453-7. PubMed PMID: 7968118.

5. Vandenbroucke JP. Observational research, randomised trials, and two views of medical science. PLoSMed. 2008;5(3):e67. doi: 10.1371/journal.pmed.0050067. PubMed PMID: 18336067; PubMed Central PMCID: PMC2265762.

6. Vandenbroucke JP. In defense of case reports and case series. Ann Intern Med. 2001;134(4):330-4. PubMed PMID: 11182844.

7. Carey JC, Martinez L, Balken E, Leen-Mitchell M, Robertson J. Determination of human teratogenicity by the astute clinician method: review of illustrative agents and a proposal of guidelines. Birth Defects Res A Clin Mol Teratol. 2009;85(1):63-8. doi: 10.1002/bdra.20533. PubMed PMID: 19107954.

8. Maclure M. Demonstration of deductive meta-analysis: ethanol intake and risk of myocardial infarction. Epidemiol Rev. 1993;15(2):328-51. PubMed PMID: 8174661.

9. Shepard TH. "Proof" of human teratogenicity. Teratology. 1994;50(2):97-8. doi: 10.1002/tera.1420500202. PubMed PMID: 7801306.

10. Jick H. The discovery of drug-induced illness. N Engl J Med. 1977;296(9):481-5. doi: 10.1056/NEJM197703032960904. PubMed PMID: 834226.

11. Hill AB. The Environment and Disease: Association or Causation? Proc R Soc Med. 1965;58:295-300. PubMed PMID: 14283879; PubMed Central PMCID: PMC1898525.

12. US Department of Health E, and Welfare. Public Health Service,. Smoking and Health. Report of the Advisory Committee to the Surgeon General of the Public Health Service. Public Health Service Publication No. 1103. Washington DC: 1964. Available from: https://profiles.nlm.nih.gov/NN/B/B/M/Q/_/nnbbmq.pdf. [Last accessed 16.06.2016].

13. Rothman KJ, Greenland S. Causation and causal inference in epidemiology. Am J Public Health. 2005;95 Suppl 1:S144-50. doi: 10.2105/AJPH.2004.059204. PubMed PMID: 16030331.

14. Gordis L. Chapter 14. From Association to Causation: Deriving Inferences from Epidemiologic Studies. Epidemiology: Saunders Elsevier; 2009. p. 227-46.

15. Khangura S, Konnyu K, Cushman R, Grimshaw J, Moher D. Evidence summaries: the evolution of a rapid review approach. Syst Rev. 2012;1:10. doi: 10.1186/2046-4053-1-10. PubMed PMID: 22587960; PubMed Central PMCID: PMC3351736.

16. Dye C, Bartolomeos K, Moorthy V, Kieny MP. Data sharing in public health emergencies: a call to researchers. Bull World Health Organ. 2016;94(3):158. doi: 10.2471/BLT.16.170860. PubMed PMID: 26966322; PubMed Central PMCID: PMC4773943.

17. Zika Experimental Science Team. ZIKV-003 and ZIKV-005: Infection with French Polynesian and Asian lineage Zika virus during the first pregnancy trimester 2016 [10.05.2016]. Available from: https://zika.labkey.com/project/OConnor/ZIKV-003/begin.view?

18. Low N, Krauer F, Riesen M. Causality framework for Zika virus and neurological disorders: systematic review protocol 2016. CRD42016036693]. Available from: http://www.crd.york.ac.uk/PROSPERO/display_record.asp?ID=CRD42016036693.

19. National Institute for Health and Clinical Excellence. Developing NICE guidelines: the manual appendix H. London: National Institute for Health and Clinical Excellence, 2015. Available from: https://www.nice.org.uk/process/pmg20/resources/developing-nice-guidelines-the-manual-appendix-h-2549711485. [Last accessed 26.10.2016].

20. Thayer KA, Schunemann HJ. Using GRADE to respond to health questions with different levels of urgency. Environ Int. 2016;92-93:585-9. doi: 10.1016/j.envint.2016.03.027. PubMed PMID: 27126781.

21. Tirado SM, Yoon KJ. Antibody-dependent enhancement of virus infection and disease. Viral Immunol. 2003;16(1):69-86. doi: 10.1089/088282403763635465. PubMed PMID: 12725690.

22. Dejnirattisai W, Supasa P, Wongwiwat W, Rouvinski A, Barba-Spaeth G, Duangchinda T, et al. Dengue virus sero-cross-reactivity drives antibody-dependent enhancement of infection with zika virus. Nat Immunol. 2016;17(9):1102-8. Epub 2016/06/25. doi: 10.1038/ni.3515. PubMed PMID: 27339099.

23. Rodriguez-Barraquer I, Salje H, Lessler J, Cummings DAT. Predicting intensities of Zika infection and microcephaly using transmission intensities of other arboviruses. bioRxiv. 2016. doi: 10.1101/041095.

24. Karimi O, Goorhuis A, Schinkel J, Codrington J, Vreden SG, Vermaat JS, et al. Thrombocytopenia and subcutaneous bleedings in a patient with Zika virus infection. Lancet. 2016;387(10022):939-40. Epub 2016/02/26. doi: 10.1016/s0140-6736(16)00502-x. PubMed PMID: 26906627.

25. Tappe D, Nachtigall S, Kapaun A, Schnitzler P, Gunther S, Schmidt-Chanasit J. Acute Zika virus infection after travel to Malaysian Borneo, September 2014. Emerging infectious diseases. 2015;21(5):911-3. Epub 2015/04/22. doi: 10.3201/eid2105.141960. PubMed PMID: 25898277; PubMed Central PMCID: PMC4412240.

26. Bearcroft WG. Zika virus infection experimentally induced in a human volunteer. Trans R Soc Trop Med Hyg. 1956;50(5):442-8. Epub 1956/09/01. PubMed PMID: 13380987.

27. Henderson BE, Cheshire PP, Kirya GB, Luie M. Immunologie studies with yellow fever and selected african group b arboviruses in rhesus and vervet monkeys. AmerjTrop. 1970;Med.Hyg. 19(1):110-8. PubMed PMID: 0008235794.

28. Smithburn KC. Antigenic relationships among certain arthropod-borne viruses as revealed by neutralization tests. J Immunol. 1954;72(5):376-88. PubMed PMID: 0007367626.

29. Monath TP, Schlesinger JJ, Brandriss MW, Cropp CB, Prange WC. Yellow fever monoclonal antibodies: type-specific and cross-reactive determinants identified by immunofluorescence. The American journal of tropical medicine and hygiene. 1984;33(4):695-8. Epub 1984/07/01. PubMed PMID: 6206738.

30. Fagbami AH, Halstead SB, Marchette NJ, Larsen K. Cross-infection enhancement among African flaviviruses by immune mouse ascitic fluids. Cytobios. 1987;49(196):49-55. Epub 1987/01/01. PubMed PMID: 3028713.

31. Macnamara FN, Horn DW, Porterfield JS. Yellow fever and other arthropod-borne viruses. a consideration of two serological surveys made in south western nigeria. Transactions of the Royal Society of Tropical Medicine and Hygiene. 1959;53(2):425-9. PubMed PMID: 0007896402.

32. De Goes Cavalcanti LP, Tauil PL, Alencar CH, Oliveira W, Teixeira MM, Heukelbach J. Zika virus infection, associated microcephaly, and low yellow fever vaccination coverage in Brazil: is there any causal link? J Infect Dev Ctries. 2016;10(6):563-6. Epub 2016/07/02. doi: 10.3855/jidc.8575. PubMed PMID: 27367003.

33. Driggers RW, Ho CY, Korhonen EM, Kuivanen S, Jaaskelainen AJ, Smura T, et al. Zika Virus Infection with Prolonged Maternal Viremia and Fetal Brain Abnormalities. N Engl J Med. 2016;374(22):2142-51. Epub 2016/03/31. doi: 10.1056/NEJMoa1601824. PubMed PMID: 27028667.

34. Mlakar J, Korva M, Tul N, Popovic M, Poljsak-Prijatelj M, Mraz J, et al. Zika Virus Associated with Microcephaly. N Engl J Med. 2016;374(10):951-8. Epub 2016/02/11. doi: 10.1056/NEJMoa1600651. PubMed PMID: 26862926.

35. Evans D, Nijhout F, Parens R, Morales AJ, Bar-Yam Y. A Possible Link Between Pyriproxyfen and Microcephaly. 2016. Available from: arXiv:1604.03834. Cited 13 April 2016.
