# Supplementary material for: Zika Virus Infection as a Cause of Congenital Brain Abnormalities and Guillain–Barré Syndrome: Systematic Review
Source: PLoS Med. 2017 Jan 3;14(1):e1002203. doi: 10.1371/journal.pmed.1002203 (PMC5207634; doi:10.1371/journal.pmed.1002203)
Supplement: S2 Text — From PAHO Epidemiological Update 28 April 2016 (Fig 7, p7). (PDF) [file pmed.1002203.s003.pdf]

S2 Text

In Pernambuco state, Brazil, data about Zika-like illness have been collected since January 2015. The Secretaria de Pernambuco produced epidemic curves of reported cases of dengue, chikungunya, Zika and microcephaly by epidemiological week. The figure below shows a large epidemic of dengue in 2015 at the time of the first reported cases of Zika-like illness. The peak in reported cases of microcephaly followed approximately 30 weeks later in 2016. The figure appeared in the PAHO Epidemiological Update 28 April 2016 (Figure 7, p7) reference [21] in the main text.

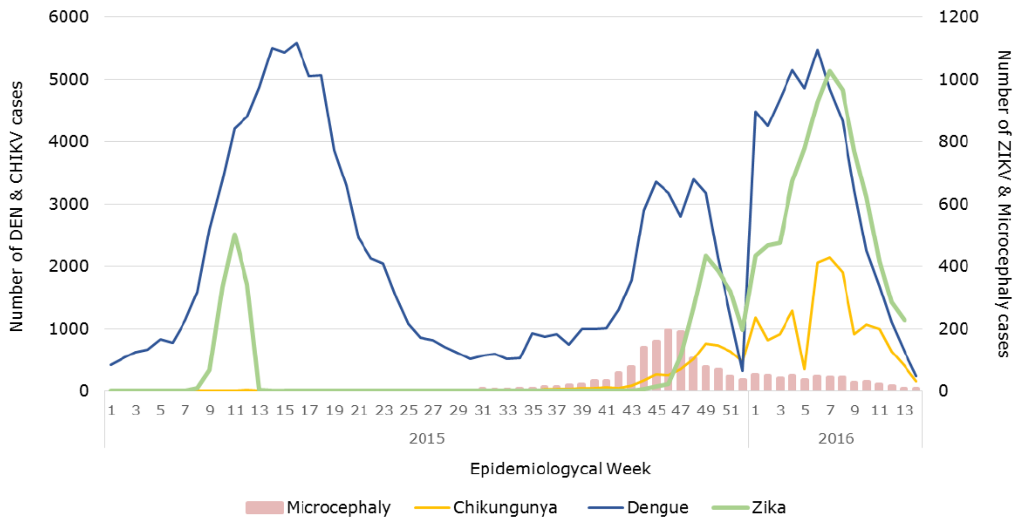

Source: Data published by the Pernambuco State Secretary of Health, Brazil.
