# Supplementary material for: Zika Virus Infection as a Cause of Congenital Brain Abnormalities and Guillain–Barré Syndrome: Systematic Review
Source: PLoS Med. 2017 Jan 3;14(1):e1002203. doi: 10.1371/journal.pmed.1002203 (PMC5207634; doi:10.1371/journal.pmed.1002203)
Supplement: S1 Table — (PDF) [file pmed.1002203.s004.pdf]

S1 Table

S1 Table. Bradford Hill's "viewpoints" of causation, modifications by Gordis and adaptation to the dimensions of the causality framework, in order

Order	Bradford Hill viewpoints[a] [11]	Gordis guidelines I[b] [14]	Gordis guidelines II[c] [14]	Zika causality framework dimensions	Explanation of modifications to the Bradford Hill and Gordis lists
1	Strength (listed 2nd in [12])	Temporal relationship	Temporal relationship (major)	Temporality	Same as Gordis I and II
2	Consistency of observed association (listed 1st in [12])	Strength of the association	Biological plausibility (major)	Biologic plausibility	Same as Gordis I and II
3	Specificity (same rank as in [12])	Dose-response relationship	Consistency (major)	Strength of association	Strength of association moved up because of the importance of comparing exposed and non-exposed populations. Consistency moved to the end because its assessment summarises evidence from studies that have addressed the other dimensions.
4	Temporality (same rank as in [12])	Replication of the findings (maps onto consistency in Bradford Hill's list)	Alternative explanations (major)	Exclusion of alternate explanations	Same as Gordis I and II
5	Biological gradient	Biologic plausibility	Dose-response relationship (other)	Cessation	See above
6	Plausibility	Consideration of alternate explanations (not in Bradford Hill's list)	Strength of the association (other)	Dose-response relationship	Dose-response relationship moved down because of a priori assumption that data would be scarece
7	Coherence (listed 5th in [12])	Cessation of exposure (maps onto experiment in Bradford Hill's list)	Cessation effects (other)	Animal experiments	This dimension allows "experiment" to mean either laboratory experiments or clinical trials [13]
8	Experiment	Consistency with other knowledge (maps onto coherence in Bradford Hill's list)		Analogy	Taken from Bradford Hill's list
9	Analogy	Specificity		Specificity	Taken from Bradford Hill's list
10				Consistency of the association	

a. Bradford Hill's list and relationship to the criteria used by the advisory group to the US Surgeon General's report on smoking and health [12];

b. Gordis guidelines I are the adaptation of items used by Bradford Hill [11] and the US Surgeon General's report on smoking and health [12];

c. Gordis guidelines II are the items in the US Public Health Service modified list to evaluate evidence of a causal relationship (cited in [14]). The items are split into major criteria and other considerations.

S1 Table

References

Reference numbers are the same as those in S1 Text

11. Hill AB. The Environment and Disease: Association or Causation? Proc R Soc Med. 1965;58:295-300. PubMed PMID: 14283879; PubMed Central PMCID: PMC1898525.

12. US Department of Health E, and Welfare. Public Health Service,. Smoking and Health. Report of the Advisory Committee to the Surgeon General of the Public Health Service. Public Health Service Publication No. 1103. Washington DC: 1964. Available from: https://profiles.nlm.nih.gov/NN/B/B/M/Q/_/nnbbmq.pdf. [Last accessed 16.06.2016].

13. Rothman KJ, Greenland S. Causation and causal inference in epidemiology. Am J Public Health. 2005;95 Suppl 1:S144-50. doi: 10.2105/AJPH.2004.059204. PubMed PMID: 16030331.

14. Gordis L. Chapter 14. From Association to Causation: Deriving Inferences from Epidemiologic Studies. Epidemiology: Saunders Elsevier; 2009. p. 227-46.
