# Supplementary material for: Zika Virus Infection as a Cause of Congenital Brain Abnormalities and Guillain–Barré Syndrome: Systematic Review
Source: PLoS Med. 2017 Jan 3;14(1):e1002203. doi: 10.1371/journal.pmed.1002203 (PMC5207634; doi:10.1371/journal.pmed.1002203)
Supplement: S2 Table — (PDF) [file pmed.1002203.s005.pdf]

S2 Table

S1 Table. Zika causality framework, version 9

1. Temporality

	Congenital abnormalities	Guillain-Barré syndrome
**Individual level data**	**1.1a.** Does Zika virus (ZIKV) infection precede the development of congenital abnormalities in individuals?<br>**1.2.** Is the timing of Zika infection during gestation and the observed pattern of congenital abnormalities compatible with the expected stage of embryological development?	**1.1a.** Does ZIKV infection precede the development of Guillain-Barré syndrome in individuals?<br>**1.2.** Is the interval between exposure to ZIKV and occurrence of symptoms typical for para- or post-infectious Guillain-Barré syndrome?
**Study types**	Cohort study, case-control study, case series, case report	
**Population level data**	**1.1b.** Is there a consistent time-dependent relationship between the occurrence of ZIKV cases and cases with the outcome of interest at population-level?	
**Study types**	Ecological time-trend study	

2. Biological plausibility

	Congenital abnormalities	Guillain-Barré syndrome
**Individual level data** [a.]	**2.1.** Which cell receptor(s) bind the ligand of ZIKV in humans?<br>**2.2.** Which tissues express such receptor(s) and at which gestational age are they expressed?<br>**2.3.** Can ZIKV particles be found in the placenta, umbilical cord blood and/or amniotic fluid of previously or currently infected mothers and if yes, with what probability?<br>**2.4.** Are the ZIKV particles in the placenta/amniotic fluid/umbilical cord infectious/capable of replication?<br>**2.5.** Can ZIKV particles be found in brain or other tissues of cases with congenital abnormalities?<br>**2.6.** Are the ZIKV particles found in the brain infectious/capable of replication?<br>**2.7.** Are there experimental studies that describe plausible biological mechanisms by which ZIKV infection could lead to congenital abnormalities?	**2.1.** Do ZIKV epitopes mimic host antigens (molecular mimicry)?<br>**2.2.** Does ZIKV infection lead to an increase in detectable autoreactive immune cells or autoreactive antibodies?<br>**2.3.** Are there other biologically plausible mechanisms by which ZIKV infection could lead to GBS?
**Study types**	Basic research (protein expression, cell culture), case report, case-control study, cohort study, systematic reviev	

3. Strength of the association

	Congenital abnormalities	Guillain-Barré syndrome
**Individual level data**	**3.1a.** How strong is the association between ZIKV infection and the outcome of interest at the individual level?	
**Study types**	Cohort study, case-control study, cross-sectional	
**Population level data**	**3.1b.** How strong is the association between ZIKV infection and the outcome of interest at the population level?	
**Study types**	Ecological study	

S2 Table

4. Exclusion of alternative explanations		
	**Congenital abnormalities**	**Guillain-Barré syndrome**
**Individual level data**	Have other explanations/confounders been excluded, such as<br>**4.1.** TORCHS or other congenital infections<br>**4.2.** Maternal exposure to toxic chemicals (heavy metals, pesticides, drugs, alcohol, others)<br>**4.3.** Maternal or foetal malnutrition<br>**4.4.** Hypoxic-ischaemic lesions<br>**4.5.** Genetic conditions<br>**4.6.** Radiation	Have other explanations/confounders been excluded, such as<br>**4.1.** Other infections<br>**4.2.** Vaccines<br>**4.3.** Underlying systemic disease<br>**4.4.** concomitant medication, drugs or other chemicals
**Study types**	Case-control study, cohort study, case series, case report, environmental sampling	

TORCHS, toxoplasmosis, rubella, cytomegalovirus, herpes simplex, syphilis

5. Cessation/reversibility/preventability		
	**Congenital abnormalities**	**Guillain-Barré syndrome**
**Individual level data**	**5.1a.** Does the intentional prevention/removal/elimination of ZIKV infection in individuals, e.g. by insect repellents, lead to a reduction in cases with the outcome of interest?	
**Study types**	Randomised controlled trials of insect repellents or other interventions	
**Population level data**	**5.1b.** Does the intentional removal/elimination/prevention of ZIKV at population-level, e.g. by vector control, lead to a reduction in cases with the outcome?<br>**5.2b.** Does a natural removal/elimination/prevention of ZIKV at population-level, e.g. increase in immune individuals or decrease in vector abundance lead to a reduction in cases with the outcome?	
**Study types**	Experimental vector control interventions, ecological time-trend study, seroprevalence study	

6. Dose-response relationship (biological gradient)		
	**Congenital abnormalities**	**Guillain-Barré syndrome**
**Individual level data**	**6.1.** Are the risk and the clinical severity of congenital abnormalities associated with the viral load in maternal serum, urine, the placenta and/or amniotic fluid?<br>**6.2.** Are the risk and the clinical severity of congenital abnormalities associated with the clinical severity (including being asymptomatic) of ZIKV infection in the mother?	**6.1.** Are the risk and the clinical severity of Guillain-Barré syndrome associated with viral titres or viral load in the urine?
**Study types**	Cohort study, case series, case reports	

7. Animal experiments		
	**Congenital abnormalities**	**Guillain-Barré syndrome**
**Individual level data**	**7.1.** Does inoculation of pregnant female animals with ZIKV cause congenital abnormalities in the offspring?<br>**7.2.** Does intracerebral inoculation of animals with ZIKV lead to viral replication in the central nervous system?<br>**7.3.** Does any other route of inoculation of animals with ZIKV lead to viral replication in the central nervous system?<br>**7.4**. Do other experiments with animals or animal-derived cells support the association of ZIKV infection and congenital abnormalities?	**7.1.** Does inoculation of animals with ZIKV lead to an autoimmune reaction resulting in peripheral neuropathy?<br>**7.2.** Do other animal experiments support the association of ZIKV infection and Guillain-Barré syndrome?

S2 Table

Study types	Basic research: animal experiments

8. Analogy		
	**Congenital abnormalities**	**Guillain-Barré syndrome**
**Individual level data**	**8.1.** Do other flaviviruses or arboviruses cause the outcome of interest and by which mechanism(s)?<br>**8.2.** Do other pathogens cause the outcome of interest and by which mechanism(s)?<br>**8.3.** Which pathogen or host factors facilitate the development of the outcome of interest?	
**Study types**	Systematic review	

9. Specificity		
	**Congenital abnormalities**	**Guillain-Barré syndrome**
**Individual level data**	**9.1.** Are there pathological findings in cases with the outcome that are specific for ZIKV infection?	
**Study types**	cohort study, case-control study, case series, case report, review	

10. Consistency / replicability		
	**Congenital abnormalities**	**Guillain-Barré syndrome**
**Individual level data**	Is the association between ZIKV infection and cases with the outcome consistently found across different<br>**10.1.** Geographical regions<br>**10.2.** Populations/subpopulations<br>**10.3.** ZIKV lineages/strains<br>**10.4.** Study designs	
**Study types**	Systematic reviews, other appropriate epidemiological study types	

Co-factors		
	**Congenital abnormalities**	**Guillain-Barré syndrome**
**Individual level data**	Are there co-factors contributing to the development the outcome after ZIKV infection such as<br>**1.** Concurrent or previous dengue infection (antibody dependent enhancement)<br>**2.** Other concurrent or previous infections<br>**3.** Underlying systemic diseases<br>**4.** Polymorphism/deletion of specific host immune system genes or general genetic predisposition<br>**5.** Specific pathogen factors<br>**6.** Concomitant medication, recreational drugs or other chemicals<br>**7.** Other	
**Study types**	Cohort study, case-control study, case series, case report	
