# Supplementary material for: Zika Virus Infection as a Cause of Congenital Brain Abnormalities and Guillain–Barré Syndrome: Systematic Review
Source: PLoS Med. 2017 Jan 3;14(1):e1002203. doi: 10.1371/journal.pmed.1002203 (PMC5207634; doi:10.1371/journal.pmed.1002203)
Supplement: S3 Table — (PDF) [file pmed.1002203.s006.pdf]

S3 Table

S1 Table. Characteristics of included and excluded items, total 1091 unique items

	Excluded items		Included items		
	Number of items	%	Number of items	%	p-value
**Source**					<0.001
ArXiv	8	0.8	0	0	
BiorXiv	36	3.7	8	7.5	
CDC	2	0.2	0	0	
ECDC	26	2.6	1	0.9	
EID	6	0.6	2	1.9	
Embase	124	12.6	3	2.8	
Eurosurveillance	3	0.3	1	0.9	
F1000	16	1.6	0	0	
Institute de veille sanitaire	22	2.2	1	0.9	
Lancet	12	1.2	0	0	
LILACS	21	2.1	0	0	
Other	0	0	1	0.9	
PAHO	2	0.2	19	17.9	
PeerJ Preprints	4	0.4	2	1.9	
PLoS	3	0.3	0	0	
Pubmed	670	68	65	61.3	
WHO	15	1.5	2	1.9	
WHO Zika open	15	1.5	1	0.9	
Total	*985*	*100*	*106*	*100*	
**Type of information**					<0.001
Scientific (journal) article	837	85	82	77.4	
Surveillance report or data	75	7.6	23	21.7	
News item or lay press	70	7.1	0	0	
Biological database	0	0	1	0.9	
Other	3	0.3	0	0	
Total	*985*	*100*	*106*	*100*	
**Peer-reviewed**					0.015
No	120	14.3	16	19.5	
Yes	508	60.7	57	69.5	
Unknown/unclear	209	25	9	11	
Total (scientific journal articles)	*837*	*100*	*82*	*100*	
**Type of scientific article**					<0.001
Original research	372	44.4	81	98.8	
Review	116	13.9	1	1.2	
Comment/Editorial/Perspective/Letter	340	40.6	0	0	
Other	9	1.1	0	0	
Total (scientific journal articles)	*837*	*100*	*82*	*100*	
**Type of research**					0.005
Clinical/epidemiological research	376	66.8	73	68.9	
Basic and applied biomedical research	123	21.8	32	30.2	
Environmental and vector research	63	11.2	1	0.9	
Other	1	0.2	0	0	
Total (original research, review, surveillance report or biological database)	*563*	*100*	*106*	*100*	
**Type of study**					<0.001
Case report	52	9.3	18	17	
Case series	26	4.6	27	25.5	
Case-control study	0	0	1	0.9	
Cohort study	3	0.5	1	0.9	
Cross-sectional study	60	10.7	1	0.9	
Diagnostic study	7	1.2	0	0	
Ecological study/outbreak report	84	14.9	23	21.7	

S3 Table

Guidelines	13	2.3	0	0	
Modelling study	27	4.8	2	1.9	
Qualitative study	9	1.6	0	0	
Epidemiological/clinical review	91	16.2	0	0	
Other clinical/epidemiological	4	0.7	0	0	
Animal experiment	17	3	18	17	
In vitro experiment	16	2.8	10	9.4	
Sequence analysis and phylogenetics	47	8.4	4	3.8	
Basic research review	13	2.3	0	0	
Biochemical/protein structure studies	7	1.2	0	0	
Other basic research	23	4.1	0	0	
Vector competence studies	8	1.4	0	0	
Ecological/animal cross-sectional	27	4.8	1	0.9	
Ecological/vector modelling	4	0.7	0	0	
Ecological/Vector review	8	1.4	0	0	
Vector control studies	8	1.4	0	0	
Other ecological	8	1.4	0	0	
Total (original research, review, surveillance report or biological database)	*562*	*100*	*106*	*100*	
**Decade of publication**					0.221
1950-1959	18	1.8	5	4.7	
1960-1969	23	2.3	1	0.9	
1970-1979	24	2.4	2	1.9	
1980-1989	17	1.7	0	0	
1990-1999	12	1.2	0	0	
2000-2009	16	1.6	1	0.9	
2010-present	875	88.8	97	91.5	
Total	*985*	*100*	*106*	*100*	
