# Supplementary material for: Zika Virus Infection as a Cause of Congenital Brain Abnormalities and Guillain–Barré Syndrome: Systematic Review
Source: PLoS Med. 2017 Jan 3;14(1):e1002203. doi: 10.1371/journal.pmed.1002203 (PMC5207634; doi:10.1371/journal.pmed.1002203)
Supplement: S4 Table — (PDF) [file pmed.1002203.s007.pdf]

S4 Table. Causality framework evidence for Zika virus infection and congenital brain abnormalities, including microcephaly

Contents

S4 Table. Causality framework evidence for Zika virus infection and congenital brain abnormalities, including microcephaly 1
1. Temporality 2
2. Biological plausibility 3
3. Strength of association 9
4. Exclusion of alternative explanations 10
5. Cessation 12
6. Dose-response relationship 13
7. Animal experiments 14
8. Analogy 21
9. Specificity 22
10. Consistency 23
References 25

S4 Table

Question no.	Causality dimension and question	Study type, number [reference] of primary items included	Linked items [reference]	Country of study (clinical studies only)	Support causality dimension	Do not support causality dimension	Summary of evidence
1. Temporality		Summary: Total, 35 items in 21 groups reviewed. Reviewer assessments found sufficient evidence for all 3 questions of an appropriate temporal relationship between Zika virus (ZIKV) infection and the occurrence of congenital abnormalities, including microcephaly. The period of exposure to ZIKV was most likely to be in the first or early second trimester of pregnancy.					
1.1a	Does ZIKV infection precede the development of congenital abnormalities in individuals?	Case report, 7 [34, 39, 52, 67, 80, 85, 90]; Case series, 9 [32, 35-37, 40, 45, 51, 68, 89]; Cross-sectional: 1 [88]; Cohort, 1 [42]	[43, 65, 73, 74, 76] linked to [68]; [41, 75] linked to [51]; [30] linked to [37]; [31, 33] linked to [88]	Brazil, Colombia, French Polynesia, Martinique, Slovenia,[a] United States of America (USA)[a]	18	0	Exposure to ZIKV reported or assessed before the outcome:<br>• 180 of 265 mothers in 22 studies reported ZIKV-related symptoms during pregnancy [32, 34-37, 39, 40, 42, 45, 51, 52, 68, 80, 85, 88-90];<br>• 19 of 32 mothers with laboratory confirmed ZIKV infection had ZIKV antibodies or reverse transcriptase PCR (RT-PCR) positive result before the detection of foetal malformations (14 in Brazil, 2 in Colombia, 1 in Martinique, 2 returning travellers) [40, 42, 45, 52, 67].
1.1b.	Is there a consistent time-dependent relationship between the occurrence of ZIKV cases and cases with congenital abnormalities at population-level?	Ecological study/ outbreak report, 2 [57, 78]	[46, 49, 60, 86] linked to [78]	Brazil	2	0	Temporal or spatiotemporal relationship between Zika case reports and congenital abnormalities in French Polynesia and Brazil. The time lag between Zika virus epidemic peak and microcephaly epidemic peak was approximately:<br>• 34 weeks in Pernambuco State, Brazil (see S3 Figure) [57];<br>• 30 weeks in Bahia State, Brazil [78].
1.2	Is the timing of ZIKV infection during gestation and the observed pattern of congenital anomalies compatible with the expected stage of embryological development?	Case report, 6 [34, 39, 52, 80, 85, 90]; Case series, 8 [32, 35-37, 40, 51, 68, 89]; Cohort study, 1; Cohort, 1 [42]; Cross-sectional: 1 [88];	[43, 65, 73, 74, 76] linked to [68]; [41, 75] linked to [51]; [30] linked to [37]; [31, 33] linked to [88]; [46, 49, 60, 86] linked to [78]	Brazil, Slovenia,[a] USA,[a] French Polynesia	18	0	Several studies in which the foetus or neonate had congenital abnormalities suggest that the most likely period of exposure to ZIKV was during the first or early second trimester:<br>• 117 women (105 in Brazil, 6 in French Polynesia, 6 returning travellers) in 15 studies provided data about the presumed time point of exposure, of which 69.2% (81) were in the first trimester, 23.9% (28) in the second trimester and 6.9% (8) in the third trimester [32, 34-37, 39, 40, 42, 51, 52, 68, 80, 85, 88-90];

		Ecological study/ outbreak report, 1 [78];<br>Modelling study, 1 [47]					• Several ecological studies assessed the most likely time point of exposure as the first [60, 78, 86] or the early second trimester [60] using SINASC and RESP data from Bahia State, Brazil. Based on data from Bahia State, the risk of microcephaly in the first trimester due to Zika virus infection was calculated to range from 0.88% (95% CI 0.8-0-97%) to 13.2% (95% CI 12.0-14.4%) [86];<br>• A statistical model for an ecological study using state-level data assessed the most likely time point of exposure in Brazil between gestational week 14 (95% CI +/- 0.08 weeks) and gestational week 17 (95% CI +/- 0.11 weeks) [49];<br>• A statistical model for a retrospective study in French Polynesia assessed the most likely time point of exposure as the first trimester. The risk of microcephaly following maternal ZIKV infection in the first trimester was calculated as 1% [47];
2. Biological plausibility		Summary: Total, 28 items in 25 groups reviewed. Reviewer assessments found sufficient evidence for 6 of 7 questions that address biologically plausible mechanisms by which ZIKV could cause congenital abnormalities.					
2.1	Which cell receptor(s) bind the ligand of ZIKV in humans?	*In vitro* experiment, 1 [29]	None	N/A	1	0	Findings from one study suggest that the phosphatidylserine-sensing receptor tyrosine kinase AXL is essential for viral entry into cells. Study aimed to identify entry receptors for a ZIKV strain from the French Polynesian outbreak. HEK293T (human embryonic) cells expressing the c-lectin DC-SIGN, AXL or Tyro3 had strongly enhanced viral replication, while pharmacological inhibition or silencing of AXL inhibited viral replication. Similarly, A549 (human adenocarcinoma alveolar epithelial cells) cells expressing TIM-1 and AXL, but not DC-SIGN showed strong inhibition of viral replication on inhibition or silencing of AXL. Human skin fibroblasts also showed a complete inhibition of viral replication upon silencing of AXL [29].

S4 Table

2.2	Which tissues express such receptor(s) and at which gestational age are they expressed?	*In vitro* experiment, 1 [64]	None	N/A	1	0	Whole transcriptome sequencing of human developing cerebral cortex tissue showed high levels of AXL expression mainly in radial glia cells, astrocytes, endothelial cells and microglia. The spatial expression pattern of AXL was then determined using cerebral organoids derived from human induced pluripotent stem cells (hiPSCs). Immunohistochemistry (IHC) showed strong expression in the borders of the ventricle, in radial glia-like cells and surrounding the brain capillaries of organoids at the mid-neurogenesis stage. Expression in neuronal populations was less pronounced. The examination of AXL expression at gestational week 13.5 showed persistent expression in radial glia and around capillaries. Expression of AXL in human retinal samples of gestational weeks 10 and 12 was found mainly in cells with a stem cell signature by mRNA seq. Immunostaining confirmed AXL expression on the outer margin of the retina [64].
2.3	Can ZIKV particles be found in the placenta, umbilical cord blood and/or amniotic fluid of previously or currently infected mothers and if yes, with what probability?	Case report, 4 [38, 52, 67, 85];<br>Case series, 6 [36, 37, 40, 51, 55-57, 79]	[41, 75] linked to [51];<br>[30] linked to [37];	Brazil, French Polynesia, Martinique, Panama, USA[a]	10	0	ZIKV was found in placenta, amniotic fluid and umbilical cord blood of mother-infant pairs in Brazil, Martinique, French Polynesia, Panama and in returning travellers:<br>• Amniocentesis confirmed presence of ZIKV by RT-PCR in 9 women (3 in Brazil, 4 in French Polynesia, 1 in Martinique, 1 returning travellers) [37, 38, 51, 52, 67];<br>• ZIKV detected by RT-PCR or IHC in placental tissue or products of conception following miscarriages in 7 women (4 in Brazil, 3 returning travellers). IHC of the placenta of one neonatal death showed immunopositivity mainly in Hofbauer cells and some histiocytes in intervillous spaces [36, 40, 52, 79, 85];<br>• ZIKV was detected by RT-PCR in the umbilical cord tissue of 2 babies (1 returning traveller, 1 in Panama) [52, 55].
2.4	Are the ZIKV particles in the placenta/amniotic fluid/umbilical cord	Case report, 1 [52];<br>*In vitro* experiment, 1 [72]	None	USA[a]	0	2	• ZIKV isolated in placental tissue, following termination of pregnancy in a traveller returning from the Americas, did not replicate in SK-N-SH or Vero E6

	infectious/capable of replication?						cells suggesting that the virus particles were not infectious [52];<br>• *In vitro* experiment with primary human trophoblasts (PHT) from full-term placentas, infected with the Asian or African ZIKV lineage, showed no evidence for viral replication suggesting that ZIKV cannot replicate efficiently in PHT. The replication appeared to be inhibited by Type III Interferon produced by syncytiotrophoblasts. The authors concluded that ZIKV might not be transmitted vertically through trophoblasts but through other placental cells such as macrophages. The effect of ZIKV on PHT from first or second trimester placentas was not assessed [72].
2.5	Can ZIKV particles be found in brain or other tissues of cases with congenital abnormalities?	Case report, 4 [34, 38, 52, 61]; Case series, 2 [36, 79]	None	Brazil, Slovenia[a], USA[a]	6	0	ZIKV detected by RT-PCR and/or IHC in tissues from 8 foetuses or neonates:<br>• In brain tissue after stillbirth (1), neonatal death (5) or pregnancy termination (2) (6 in Brazil, 2 travellers returning from the Americas). Histopathological examination of the brain after neonatal death (3) showed the presence of ZIKV mainly in glial cells [34, 36, 38, 52, 79];<br>• In other foetal tissues (muscle, lung, liver and spleen) after termination of pregnancy (1 case report) [52], but not in other foetal tissues from the other 7 cases [34, 36, 38, 79];<br>• In undefined tissue and blood of a neonate who died shortly after birth in Brazil [61].
2.6	Are the ZIKV particles found in the brain infectious/capable of replication?	Case report, 1 [52]; *in vitro* experiment, 6 [44, 63, 69, 77, 81, 91]	None	USA[a]	7	0	Evidence from 1 clinical case report and 6 *in vitro* studies suggest that ZIKV is able to replicate in human brain cells:<br>• ZIKV isolated in the foetal brain following termination of pregnancy in a traveller returning from the Americas showed replication in both SK-N-SH and Vero E6 cells suggesting that the virus particles isolated in the brain were infectious and capable of replication [52];

S4 Table

							• The African ZIKV strain MR766 was shown to replicate in forebrain-specific human neural progenitor cells (hNPCs), neurons derived from hiPSCs and human cells derived from glioblastoma and astrocytoma. Infectivity was confirmed by inoculation of Vero cells with the supernatant of hNPCs [44];<br>• The African ZIKV strain MR766 was also able to infect and replicate in neural stem cells (NSC), neurospheres and brain organoids derived from hiPSCs. Infected neurospheres showed morphological abnormalities and cell detachments, infected organoids had a reduced growth compared to controls [63];<br>• Inoculation of SH-SY5Y (human neuroblastoma) cells with both the African ZIKV strain MR766 and the Brazilian ZIKV strain led to viral replication confirmed by quantitative (q) PCR [69];<br>• Neurospheres grown from hiPCS induced neural stem cells were significantly smaller than controls when infected with the Brazilian strain ZIKV at day 3 post inoculation. At day 6, the number of infected neurospheres was reduced by 50% and at day 12, the infected neurospheres had completely disappeared indicating cell death due to viral replication [91];<br>• Human embryonic stem cell (hESC) derived cerebral organoids exposed to ZIKV MR766 showed significant decreased growth compared to mock treated controls, and RT-PCR confirmed increased viral copy number reflecting effective replication. Immunostaining indicated the presence of ZIKV mainly in neural progenitor cells (NPCs) [81];<br>• Forebrain-specific organoids grown from hiPSCs were infected with ZIKV MR766 at early organoid stage. Compared to controls, infected organoids showed decreased size and reduced ventricular zone thickness suggestive of cell death and suppression of neural progenitor cell (NPC) proliferation. The infected cells

							were predominantly NPCs in early stage organoids, and NPCs and astrocytes in later stage organoids [77].
2.7	Are there experimental studies that describe plausible biological mechanisms by which ZIKV infection could lead to congenital abnormalities?	*In vitro* experiment, 6 [29, 44, 63, 81, 91, 93]; sequence analysis and phylogenetics, 3 [54, 58, 92]	None	N/A	9	0	Three *in silico* studies and six *in vitro* experiments provide some insight into potential biological mechanisms:<br>• An *in silico* approach found sequence complementarity of the ZIKV genome to human micro RNA (miRNA), which targets cell cycle genes such as cyclin-dependent kinase 6. Mutations of these genes are associated with neuronal or developmental disorders. Moreover, viral miRNA was predicted to target human genes such as PEX26, FGF2, SBF1, HOOK3 or PLEKHG4. These genes are also associated with neuronal or developmental disorders including microcephaly. These findings suggest a potential role of viral miRNA-mediated interference with human genes and human miRNA-mediated interference with viral genome resulting in a dysregulation of the brain development [58];<br>• An *in silico* prediction of human and viral B-cell epitopes found human proteins involved in neurophysiological function such as pro-neuropeptide Y or NAV2 to mimic viral epitopes suggesting a potential role of autoimmunity in the pathogenesis of congenital abnormalities [54];<br>• *In silico* sequence comparison of the ZIKV genome and human nucleotides associated with microcephaly resulted in a high overlap of the non-structural protein NS4b of ZIKV with the human gene astrotactin 2 (astn2), which is expressed during developmental stages in migrating, cerebellar granule neurons [92];<br>• Primary human fibroblasts infected with French Polynesian ZIKV showed numerous double-membrane intracytoplasmic vacuoles that are characteristic of autophagosomes. The addition of an

							autophagy inhibitor decreased viral replication. These findings suggest that autophagy induced by ZIKV might contribute to the ZIKV pathogenesis [29];<br>• ZIKV infection reduced human embryonic cortical neural progenitor cell viability by 29.9% and lead to a higher activation of caspase-3 compared to mock-infected controls. Transcriptome analyses showed a downregulation of genes involved in the cell-cycle and an upregulation of genes for protein transport and catabolic/apoptotic processes suggesting that ZIKV may dysregulate the cell cycle and the transcription in neural progenitor cells [44];<br>• NSCs and cerebral organoids derived from hiPSCs showed the formation of apoptotic nuclei and an increased expression of caspase 3/7 after infection with ZIKV MR766. [63]. Upon infection with the Brazilian strain neurospheres also showed reduced expression of Nestin and SOX2 suggesting a reduction in progenitor cells. Infected cells were also predominantly in a sub-G1 mitotic phase. RNA-seq analysis showed 199 downregulated and 259 upregulated proteins in ZIKV infected cells compared to controls. Downregulated proteins included proteins of the neurogenic programme and responsible for extracellular structure organisation, organelle location, synapse organisation, regulation of neuron development and cell morphogenesis. Upregulated proteins involved proteins for the cell cycle arest as well as viral replication and included in translation, cell cycle, intracellular transport, ribosome assembly [91]<br>• hESC derived, immature cerebral organoids exposed to MR766 showed upregulation of the toll-like receptor 3 (TLR3) and treatment of organoids with a TLR3 inhibitor appeared to reverse the ZIKV-mediated shrinkage and apoptosis. These findings suggest that

							activation of TLR3 may play a pivotal role in the ZIKV pathogenesis of the developing brain and that ZIKV infection may activate the TLR3 pathway leading to dysregulation of neurogenesis [81];The infection of Human lung epithelial cells (A549) with the French Polynesian strain (PF-25013-18) and an additional autophagy inhibitor or activator did not show any significant changes in ZIKV replication suggesting that autophagy may not play a major role in ZIKV replication. PARP, a marker for apoptosis, was significantly increased in ZIKV infected cells compared to mock-infected controls. Moreover, infected cells showed an increased transcription of the SOD2 gene indicating induction of apoptosis through the accumulation of mitochondrial ROS [93];
3. Strength of association		Summary: Total, 7 items in 3 groups reviewed. Reviewer assessments found sufficient evidence of a strong association between ZIKV infection and congenital abnormalities for 2 of 2 questions. At the individual level, the effect size was extremely high, although imprecise, in 1 study and is likely to be high in the other study when follow-up is complete. At the population level, there is strong evidence of an association.					
3.1	How strong is the association between ZIKV infection and congenital abnormalities at the individual level?	Cohort study, 1 [42]; Modelling study, 1 [47]	None	Brazil, French Polynesia	2	0	2 studies in different countries found evidence of a strong positive association between maternal exposure to ZIKV and congenital abnormalities:<br>• 1 cohort study in the state of Rio de Janeiro (Brazil) enrolled 88 women pregnant women with rash and fever. Of 72 with confirmed ZIKV infection, 12 of 42 had one or more ultrasound scans with confirmed foetal brain abnormalities. In comparison, of 16 women with rash and fever due to other causes all were reported to have had normal ultrasound scans. Follow-up is incomplete so an effect size was not presented [42];<br>• 1 study collated data retrospectively about cases of microcephaly and Zika-like illness in surveillance reports during the 2013-14 outbreak in French Polynesia. The authors used statistical models to assess the likelihood of ZIKV exposure at different

							stages of pregnancy. The best fitting model yielded a risk ratio (RR) of 53.4 (95% CI 6.5-1061.2) for microcephaly in women who acquired ZIKV in the first trimester. [47].
3.2	How strong is the association between ZIKV infection and congenital abnormalities at the population level?	Ecological study/outbreak report, 2 [46, 49]	[60, 78, 86] linked to [46, 49]	Brazil	1[b]	0	2 items reporting on ecological analyses of the same sources of regional data from Brazil provide evidence of an association at population level:<br>• 1 item examined the correlation between the number of reported cases of Zika-like illness and the number of cases of suspected microcephaly per 1000 pregnancies by state. There was a moderate positive correlation (Spearman's rank rho 0.52, p=0.005) [49];<br>• 1 item compared the prevalence of microcephaly cases reported (Jan 2015 to Jan 2016) in 15 states with ZIKV transmission (2.8 per 10,000 live births, 95% CI 1.86-4.05) and 4 states without transmission (0.6 per 10,000 live births, 95% CI 0.22-1.31). The calculated prevalence ratio is 4.7 (95% CI 1.9-13.3) [46].
4. Exclusion of alternative explanations		Summary: Total, 28 items of 18 groups reviewed. Reviewer assessments found sufficient evidence at the individual level that alternative explanations have been excluded for 3 of 7 questions; no other single explanation could have accounted for clusters of congenital abnormalities. The evidence about other exposures could not be assessed because of an absence of relevant studies.					
4.1	Have other explanations/confounders of the association between ZIKV and congenital abnormalities been excluded, such as TORCHS (Toxoplasmosis; rubella; cytomegalovirus, CMV; herpes simplex virus, HSV; syphilis) or other congenital infections?	Case report, 7 [34, 38, 39, 52, 80, 85, 90];<br>Case series, 9 [32, 35-37, 45, 51, 68, 79, 89]; Cross-sectional study: 1 [88];<br>Cohort study, 1 [42]	[41, 75] linked to [51]<br>[43, 65, 73, 74, 76] linked to [68]<br>[30] linked to [37]<br>[31, 33] linked to [88]	Brazil, Colombia, French Polynesia, Slovenia,[a] USA[a]	18	0	18 groups of studies reported testing for at least one TORCHS pathogen or other infection in the mother or the baby:<br>• 18 excluded CMV: 265 individuals, 100% negative or mothers immune [32, 34-39, 42, 45, 51, 52, 68, 79, 80, 85, 88-90];<br>• 16 excluded rubella: 261 individuals, 100% negative or mothers immune [32, 34-39, 42, 45, 51, 52, 68, 79, 80, 88, 90];<br>• 17 excluded toxoplasmosis: 251 individuals, 100% negative or mothers immune [32, 34-39, 45, 51, 52, 68, 79, 80, 85, 88-90];

							• 13 excluded HSV: 245 individuals, 99.5% negative or mothers immune, 1 infant IgM positive [32, 34-37, 45, 51, 52, 68, 79, 85, 88, 90];<br>• 12 excluded acute dengue virus (DENV, IgM and/or RT-PCR): 190 individuals, 99% negative, 2 were IgM positive[34, 36, 37, 39, 42, 45, 51, 52, 68, 80, 88, 90]. 10 studies tested for DENV IgG: five cases from 4 case reports and 88% of the cohort study were found positive [34, 42, 51, 52, 90];<br>• 10 excluded HIV: 104 individuals, 100% negative [35-38, 45, 51, 80, 85, 88-90];<br>• 6 excluded chikungunya virus (CHIKV): 8 individuals, 100% negative or immune [34, 37, 39, 45, 52, 90];<br>• 3 excluded human parvovirus B19: 4 individuals, 100% negative [34, 37, 52].
4.2	Have other explanations/confounders of the association between ZIKV and congenital abnormalities been excluded, such as maternal exposure to toxic chemicals (heavy metals, pesticides, drugs, alcohol, others)?	Case series, 4 [35, 37, 51, 79]	[41, 75] linked to [51]<br>[30] linked to [37]	Brazil	4	0	• 3 studies reported exclusion of maternal alcohol consumption or drug/medication during pregnancy [35, 37, 51, 79];<br>• No study reported the exclusion of environmental toxin exposure.
4.3	Have other explanations/confounders of the association between ZIKV and congenital abnormalities been excluded, such as maternal or foetal malnutrition?	None	None	N/A	0	0	No study reported the exclusion of foetal or maternal malnutrition.

4.4	Have other explanations/confounders of the association between ZIKV and congenital abnormalities been excluded, such as hypoxic-ischaemic lesions?	None	None	N/A	0	0	No study reported the exclusion of ischaemic-hypoxic lesions during pregnancy.
4.5	Have other explanations/confounders of the association between ZIKV and congenital abnormalities been excluded, such as genetic conditions?	Case report, 2 [34, 90];<br>Case series, 1 [51]	[41, 75] linked to [51]	Brazil, French Polynesia, Slovenia[a]	3	0	3 studies reported testing for the exclusion of a genetic cause of congenital anomalies by karyotyping [34, 51, 90].
4.6	Have other explanations/confounders of the association between ZIKV and congenital abnormalities been excluded, such as radiation?	None	None	N/A	0	0	No study reported the exclusion of maternal exposure to radiation during pregnancy.
5. Cessation		Summary: Total, 6 items in 2 groups reviewed. Reviewer assessments found sufficient evidence for 1 of 3 questions. In two states of Brazil and in French Polynesia cases of congenital abnormalities decreased after ZIKV transmission ceased. Evidence for the other questions could not be assessed because no relevant studies were identified.					
5.1	Does the intentional prevention/removal/ elimination of ZIKV infection in individuals, e.g. by insect repellents, lead to a reduction in cases with congenital abnormalities?	None	None	N/A	0	0	No relevant studies identified. 1 cohort study compared the use of insect repellent in women with ZIKV (19/47, 40%) and women without ZIKV (3/10, 30%) [42]. The authors did not report on the numbers of women with abnormal ultrasound scans according to the use of insect repellent.

5.2	Does the intentional removal/elimination/prevention of ZIKV at population-level, e.g. by vector control, lead to a reduction in cases with congenital abnormalities?	None	None	N/A	0	0	No relevant studies identified
5.3	Does a natural removal/elimination/prevention of ZIKV at population-level, e.g. increase in immune individuals or decrease in vector abundance lead to a reduction in cases with congenital abnormalities?	Ecological study/outbreak report, 2 [57, 78]	[46, 49, 60, 86] linked to [78]	Brazil	2	0	At population level, a decrease in microcephaly cases was observed following a decrease in Zika cases in Pernambuco state [57] and Bahia state [78], Brazil.
6. Dose-response relationship		Summary: Total, 0 items. This dimension could not be assessed because of an absence of relevant studies					
6.1	Are the risk and the clinical severity of congenital abnormalities associated with viral load in maternal serum, urine, the placenta and/or amniotic fluid?	None	None	N/A	0	0	No relevant studies identified.
6.2	Are the risk and the clinical severity of congenital abnormalities associated with the clinical severity (including being	None	None	N/A	0	0	No relevant studies identified.

	asymptomatic) of ZIKV infection in the mother?						
7. Animal experiments		Summary: Total, 20 items reviewed. Reviewers assessments found evidence from animal experimental studies for all 4 questions that supports a causal link between ZIKV and congenital abnormalities. Inoculation with ZIKV of pregnant rhesus macaques and mice can result in foetal abnormalities, viraemia and brain abnormalities. Experiments to induce viral replication after inoculation of ZIKV intracerebrally and at other sites in a variety of animal models have produced mixed results.					
7.1	Does inoculation of pregnant female animals with ZIKV cause congenital abnormalities in the offspring?	Animal experiment, 3 [70, 82, 84]	None	N/A	3	0	• Two pregnant rhesus macaques (ZIKV-003 and ZIKV-005) were challenged subcutaneously in the first trimester with the French Polynesian and the Asian ZIKV strain, respectively. No viral RNA was detected by qRT-PCR on day 42 post infection in a sample of amniotic fluid of ZIKV-003. Initial ultrasounds of ZIKV-003 showed normal head circumference of the foetus, while later measurements at gestational day 76-81 were still in the normal range but approximately two standard deviations below the mean. Additionally, placental calcifications were observed. Compared with male animals, both pregnant females showed prolonged viral load oscillating between 500 and 5000 copies of RNA/mL until days 40-50 post inoculation in the serum, but not in the urine. These findings might suggest a foetal infection and continuous foetal shedding of virus back into the maternal circulation. These experiments are still ongoing as of August 10, 2016 [70];<br>• Female Ifnar1 -/- (interferon alpha/beta receptor KO) mice were crossed with wildtype males (C57BL/6) to generate Ifnar1 +/- heterozygous offsprings. The dams were inoculated subcutaneously on embryonic days 6.5 and 7.5 with the French Polynesian strain of ZIKV and sacrificed 7 days later. The majority of the foetuses had died and were resorbed in utero. The remaining foetuses showed significant intrauterine growth restriction. No microcephaly was observed. High levels of viral RNA were detected by RT-PCR in the placentas as well as in the heads of the foetuses.

							Moreover, histological analysis showed activation of caspase3 in the mid- and hindbrains. Viral RNA was detected by FISH in placental trophoblasts, and virions were detected by electron microscopy in the ER of mononuclear trophoblasts. Histological analyses showed destruction of placental microvasculature and apoptosis. These findings suggest that the mouse placenta is susceptible to ZIKV infection, which may lead to placental insufficiency and malfunction, and subsequent foetal growth restriction [84].<br>• Injection of ZIKV SZ01 intraperitoneally into the pregnant C57 mice showed the presence of ZIKV in the cells of the ventricular zone of the embryos. The main cell infected were radial glial cells as shown by the colocalisation of ZIKV and BLBP proteins. ZIKV infected brains also exhibited a reduced mitotic activity suggestive of inhibition of proliferation of cortical neural progenitors. Compared to mock infected controls, ZIKV infection was associated with reduced mRNA levels of genes involved in brain development and cell cycle regulation such as MCPH1, CASC5, CKD6 and others. Global transcriptome analysis confirmed that Zika virus infection led to the upregulation of genes involved in the immune response and cell death and to a downregulation of genes involved in cell proliferation and negative-regulation of apoptosis. Morphologically, the perimeters of the cortex of ZIKV infected mice were significantly shorter than that of controls, but there were no differences in cortical thickness [82].
7.2	Does intracerebral inoculation of animals with ZIKV lead to viral replication in the CNS?	Animal experiment, 10 [21-25, 27, 28, 48, 82, 83]	None	N/A	10[c]	0[c]	*In vivo* animal experiments provide inconclusive evidence of neurotropism of ZIKV through direct cerebral inoculation:<br>• Adult Swiss mice inoculated with the original ZIKV strain isolated in Uganda (MR766) showed signs of

							sickness or died in *in vivo* serial passage experiments. An increase in passage numbers was associated with an increased number of paralysed mice, increased mortality and decreased incubation time suggesting viral adaptation to the host [21, 22]. Contrarily, cotton rats, guinea pigs, rabbits, Grivet monkeys, red-tailed monkey and Rhesus macaques inoculated intracerebrally with infected mouse brain suspensions of the ZIKV MR766 strain showed no signs of illness [22];<br>• Syrian hamsters showed symptoms of nervous system involvement after intracerebral inoculation with ZIKV derived from Swiss mouse brain suspensions from serial passage experiments (MR766 strain) [24];<br>• The histopathology of Swiss mice inoculated with the serum of a human volunteer who was experimentally infected with a Nigerian strain of ZIKV, showed signs of viral encephalitis [23];<br>• The histopathology of infant Swiss mice inoculated with ZIKV isolated in *Aedes* mosquitoes from Entebbe (Uganda), showed degeneration of nerve cells mainly in the hippocampal region, “brain softening” and porencephaly after serial passaging as well as signs of myocarditis and skeletal myositis [25];<br>• The histopathology of infant and adult Swiss mice inoculated with the lyophilised ZIKV MR766 strain showed patchy destruction of astroglia cells, astrocyte hypertrophy both in infant mice (pyriform cells of Ammon's horn only) and 5 week old mice (whole cortex). Both glial and neuronal cells showed "viral factories" within the endoplasmic reticulum suggesting viral replication in both neurons and astroglial cells [27].<br>• Suckling infant Porton strain mice were more susceptible to infection and required a lower lethal

							dose (LD50) than adult mice after intracerebral inoculation with ZIKV (MR766) strain [28];<br>• Direct injection of ZIKV SZ01 (isolated from a Chinese traveller infected in Samoa) into the lateral ventricles of ICR mouse embryos on day 13.5 in utero showed effective viral replication in foetal brains mostly in the ventricular (VZ) and subventricular zone (SVZ) where NPCs are located. Compared to mock-infected controls, the brains of ZIKV infected foetuses were smaller and the ventricles were enlarged. ZIKV infected brains also showed an increased activation of caspase3 suggestive of cell death. IHC also revealed to co-localisation of ZIKV and markers of progenitor cells (Sox2, Pax6, Tbr2) suggesting that ZIKV replicates mostly in NPCs. Staining for phosphorylated H3 showed that compared to controls, ZIKV infected brains had less NPCs in the mitotic phase M, suggesting that ZIKV inhibits the cell cycle and cell differentiation. Global transcriptome analysis (RNA-seq) showed an upregulation of genes involved in the immune response, including cytokines, and in apoptosis, and a downregulation of genes involved in cell proliferation, differentiation, and cell migration [83];<br>• ZIKV SZ01 was directly injected into the lateral ventricles of in C57 mice in utero. Histological examinations some days later showed the presence of ZIKV in the cells of the ventricular zone of the dorsal telencephalon and in the striatum of the embryos [82];<br>• Two day-old BALB/c suckling mice were inoculated intracerebrally with acute phase serum of a chinese traveller returning from Samoa (ZIKV isolate SZ01). Seven days post inoculation the majority of the inoculated animals showed neurological symptoms including inactivity, hind-leg paralysis and

							hypersomnia. ZIKV infection was confirmed in the brains of the mice by RT-PCR [48].
7.3	Does any other route of inoculation of animals with ZIKV lead to viral replication in the CNS?	Animal experiment, 8 [24, 26, 50, 53, 59, 62, 71, 87]	None	N/A	8[c]	0[c]	*In vivo* animal experiments provide inconclusive evidence of neurotropism of ZIKV through direct non-cerebral inoculation:<br>• Syrian hamsters did not show signs of illness when inoculated intranasally, intralingually, intraorally, intradermally, intrarectally, intracardially and intramuscularly with infected mouse brain suspension derived from serial passage experiments with ZIKV (MR766 strain) [24];<br>• 23 monkeys of the genus colobus, cynocephalus and cercopithecus were captured in Ethiopia in 1962-1964, and the brains were harvested and examined. Histopathological lesions were found in 18 of 23 individuals, of which 12 had serological evidence for acute or past Zika virus infection (7 monoinfection, 2 in combination with YF and 3 in combination with group A viruses). The histopathological lesions included satellitosis, gliosis, involution of pyramidal cells and demyelination. All lesions indicated degenerative processes rather than acute reactive suggestive of past infections [26];<br>• After subcutaneous inoculation with the African ZIKV strain 1751, adult A129 (interferon alpha/beta receptor KO) mice showed clinical signs of infection and inflammatory and degenerative histopathology of the brains as well as condensed nuclei of hippocampal neurons and high viral loads in the brain and other organs, while wild type 129Sv/Ev mice showed no clinical or histopathological infection [53];<br>• 129/sv AG129 mice (interferon alpha/beta/gamma receptor KO) were inoculated peritoneally with ZIKV (MR766). Inoculated mice showed signs of paralysis in the lower limbs. Viral RNA was detected in brain, spleen, liver and kidney as well as in testicles by RT-

							PCR. Histopathological analyses showed viral antigens in neurons of the brain and the spinal cord as well as in hepatocytes. The brain lesions were consistent with acute neutrophilic encephalitis [62];<br>• Various immune-deficient mouse models as well as two wildtype (WT) models were inoculated subcutaneously or intravenously with several historic and contemporary ZIKV strains. Ifnar1 KO and Ifnar3/5/7 tripple KO mice showed signs of illness including neurological symptoms such as hindlimb weakness and paralysis, and died within 10 days after inoculation with ZIKV H/PF/2013. WT mice showed no signs of illness, or mortality. Immune-deficient mice inoculated with MR766 showed decreased mortality compared to the French Polynesian strains. Inoculation with the Dakar ZIKV strains showed no mortality in adult immune-deficient or WT mice, however suckling infant WT mice died within days. Viral loads for H/PF/2013 were higher in immune-deficient Ifnar1 KO mice in spleen, liver, kidney, serum, testes, brain and spinal cord than in WT mice as detected by qRT-PCR. Viral replication was confirmed in plaque assays in testes, brain and spinal cord of Ifnar1 ko, but not WT mice [71];<br>• Low-passage ZIKV from the Asian strain (FSS13025) was injected subcutaneously, intradermally or intraperitoneally in immune-deficient (A129, AG129) and in immunocompetent mice (CD1, C57B1/6J). Infected A129 and AG129 mice showed clinical signs of illness. Young mice (3 weeks) showed severe illness including tremors, and died several days post inoculation. Older animals (11 weeks) showed less severe illness and none were moribund. ZIKV was detected by Vero cell culture and IHC staining in all organs, but the highest viral loads were observed in spleen, testes and brains. Immunocompetent control

							mice showed no signs of illness and no viraemia was detected, but viral loads in organs were not established in these models [50];<br>• Six rhesus macaques were inoculated subcutaneously with a French Polynesian strain of ZIKV (3 animals) or with the African lineage (MR766) (3 animals). Viraemia was observed in all animals infected with the Asian lineage and in 2 of 3 infected with the African lineage. Compared to the African lineage, animals infected with the Asian lineage showed higher peak viraemia with the same inoculation dose. In the group exposed to the Asian lineage, ZIKV was isolated from Urine and saliva after plasma clearance. ZIKV was also detected by qRT-PCR in CSF of the Asian group suggesting viral replication in the CNS. However, infectivity of virus isolated in the CSF was not assessed. All animals showed an elevated serum creatine kinase (CK) during viraemia [59];<br>• Eight week old and three to four week old AG129 mice lacking the interferon alpha, beta and gamma receptors were inoculated subcutaneously (foot pad) or intraperitoneally with the French Polynesian ZIKV strain (H/PF/2013, log10 decreasing doses from 10E5 PFU to 1 PFU). All mice (young and adult) inoculated with 10E5 PFU ZIKV (foot pad or intraperitoneally) were moribund and euthanized on day 7 post inoculation. Mice showed signs of lethargy, but none showed signs of paralysis during illness. Viral loads were high in all tissues (liver, spleen, brain, kidney, intestine, heart, skeletal muscle and lung), but highest in the brains of young mice as detected by qRT-PCR. Histopathological examinations showed no tissue damage in heart, liver, spleen, intestines, kidney and lung). One animal had signs of inflammation and degeneration in a hindlimb muscle sample. The brains showed major pathological changes including

							neutrophil infiltration of the hippocampus and adjacent to the choroid plexus, the meninges and cortical tissue, as well as necrosis and neutrophilic infiltration of primordial germ cell regions [87]; Intraperitoneal inoculation of adult A129 (interferon receptor ko) mice with the ZIKV MR766 strain led to paralysis in the lower limbs. Histopathological analysis showed neutrophilic encephalitis and viral antigens in the neurons of brain and spinal cords [62].
7.4	Do other experiments with animals or animal-derived cells support the association of ZIKV infection and congenital abnormalities?	*In vitro* experiment, 1 [69]	None	N/A	1	0	The inoculation of primary E14 mouse cortex derived NSC with ZIKV showed active replication in both traditional cell culture and neurospheres. The pathological changes included decreased neurospheres sizes and decreased neurites formation as well as decreased neuronal branching [69].
8. Analogy		Summary: Selected studies reviewed. There are analogies with the well-described group of TORCH infections. Microcephaly has been described following the flavivirus West Nile virus (WNV) infection in pregnancy but not DENV. Evidence was not reviewed systematically.					
8.1	Do other flaviviruses or arboviruses cause congenital abnormalities and by which mechanism(s)?	Studies not found through systematic searches	N/A	N/A	N/A	N/A	• WNV: A descriptive population-wide study in the US identified 72 women who acquired laboratory confirmed WNV during pregnancy. Four women had miscarriages, 2 had elective abortions and 68 delivered live infants. 4.8% of the infants were of low birthweight and 7 had major birth defects including 2 (3%) with microcephaly (2nd trimester exposure). One infant with microcephaly and radiologically confirmed brain abnormalities died 3 days postpartum, the other had no structural abnormalities and was reported to have normal development [97].<br>• DENV: A systematic review about maternal dengue infection and pregnancy outcomes found 30 studies reporting vertical transmission of DENV from different geographical regions. The vertical transmission rate in one comparative study was reported as 1.6%. The adverse pregnancy outcomes included low birth weight and foetal death but not microcephaly or other congenital abnormalities [98].

S4 Table

8.2	Do other pathogens cause congenital abnormalities and by which mechanism(s)?	Studies not found through systematic searches	N/A	N/A	N/A	N/A	The TORCH infections, when acquired in pregnancy, cause congenital syndromes.<br>• Congenital rubella syndrome was first described in a case series in 1941, where the majority of women were exposed to rubella in the first or second month of pregnancy. The clinical spectrum of sequelae in the foetus or infant includes ophthalmological abnormalities such as cataract or microphthalmia, and cardiac defects [95].<br>• Cytomegalovirus (CMV) causes birth defects when transmitted vertically with an estimated foetal infection rate of 1.4%. The virus is believed to cross the placenta via transcytosis by binding to maternal IgG antibodies and foetal Fc receptors expressed on placental cells [96].
8.3	Which pathogen or host factors facilitate the development of congenital abnormalities?	Studies not found through systematic searches	N/A	N/A	N/A	N/A	Other congenital viral syndromes depend on the stage of pregnancy at infection, with infection in the first trimester associated with more severe abnormalities [95, 96].
9. Specificity		Summary: Total, 0 items reviewed. We did not find any studies that identified congenital abnormalities that were found following Zika virus infection in pregnancy but not in other congenital infections. The studies included described a wide range of abnormalities on clinical and neuroimaging examinations. Many of the abnormalities described are also found in other congenital infections, but with a different pattern.					
9.1	Are there pathological findings in cases with congenital abnormalities that are specific for ZIKV infection?	None	None	N/A	0	0	No relevant studies identified

S4 Table

10. Consistency		Summary: For 3 of 4 questions, the evidence assessed was consistent. By geographical region, maternal exposure to ZIKV has been associated with the occurrence of congenital abnormalities in all three regions where ZIKV has circulated since 2007. By study design, the association between ZIKV infection and congenital abnormalities has been found in studies at both individual and population level and with both retrospective and prospective designs. By population group, ZIKV infection has been linked to congenital abnormalities in both women resident in affected countries and in women from non-affected countries whose only possible exposure to ZIKV was having travelled in early pregnancy to an affected country. The evidence according to ZIKV lineage is inconsistent because an association between ZIKV and congenital abnormalities has only been reported from countries with ZIKV of the Asian lineage since 2013.					
10.1	Is an association between ZIKV infection and cases with congenital abnormalities consistently found across different geographical regions?	Ecological study/ outbreak report, 1 [13]	None	N/A	N/A	N/A	Findings across different regions partly consistent:<br>• Reports of congenital abnormalities in association with ZIKV infection have been observed in all three regions with ZIKV transmission since 2007: Pacific (French Polynesia); the Americas (Brazil, Colombia, Martinique, Panama, and in two returning travellers from countries with reported ZIKV transmission); and Africa (Cabo Verde) [13, 66];<br>• Within regions, not all countries with ZIKV transmission have reported cases of congenital abnormalities linked to maternal ZIKV infection;<br>• A review of cross-sectional studies from different countries in Africa and Asia from 1945-2014 showed ZIKV seroprevalence of 0.6-64% in African countries and 2.3-75% in Asian countries [99] suggesting that ZIKV might have been endemic in several countries for many years. No reports identified that indicated increased numbers of cases of congenital abnormalities above baseline levels in these countries. Possible explanations include small case numbers or population size, poor surveillance, high levels of immunity to ZIKV, or a lack of an association.
10.2	Is the association between ZIKV infection and cases with congenital abnormalities consistently found across different	Case report, 3 [34, 52, 85]; case series, 1 [40]	None	N/A	N/A	N/A	• Women who travelled in early pregnancy to, or who became pregnant in a ZIKV affected country have had babies with congenital abnormalities, pregnancy loss, or termination of an affected pregnancy. The fact that we observe congenital abnormalities in these travellers despite a different lifetime exposure to

	populations/ subpopulations?						other teratogenic agents shows that the association can be made across populations [34, 40, 52, 85] .<br>• No relevant studies found to have examined the association in different subpopulations within a country, e.g. by ethnic group
10.3	Is the association between ZIKV infection cases and cases with congenital abnormalities consistently found across different ZIKV lineages/strains?	Animal experiment: 1 [71],<br>*In vitro* experiment: 2 [69, 77]	None	N/A	N/A	N/A	The data on differential pathogenicity by ZIKV strain or lineage is inconsistent to date:<br>• Several African countries, where the African ZIKV lineage is presumably endemic, have not reported congenital abnormalities as a complication of ZIKV infection.<br>• An *in vitro* experiment showed that both African and Asian lineage viruses show neurotropism, but with effects that differed according to cell type. In undifferentiated cells (E14 Swiss mouse derived NSC) the Brazilian (Asian lineage) strain showed more replication than the Ugandan (African, MR766) strain. In differentiated cells (SH-SY5Y human neuroblastoma cells and E14 Swiss mouse derived neurospheres), the Ugandan strain MR766 had higher replication than the Brazilian strain [69];<br>• Infection of early-stage forebrain-specific organoids grown from hiPSCs with both the African lineage (MR766) and the Asian lineage (FSS13025 from Cambodia) showed the enrichment of ZIKV and thus viral replication in NPCs [77];<br>• Subcutaneous inoculation of various immunocompetent and immune-deficient mouse models with the Asian strain (H/PF/2013) and the African strain (MR766) showed similar survival/mortality pattern for both strains. Increased mortality was observed after intravenous inoculation of Ifnar1 ko mice with the Asian strain compared to Ifnar ko mice infected with the African strain. This observation was not made for Ifnar3/5/7 triple ko mice [71].

S4 Table

10.4	Is the association between Zika cases and cases with congenital abnormalities consistently found across different study designs?	Case report, 7 [34, 39, 52, 67, 80, 85, 90]; Case series, 9 [32, 35-37, 40, 45, 51, 68, 89]; Cross-sectional: 1 [88]; Cohort, 1 [42] Ecological study/ outbreak report, 2 [57, 78]	[43, 65, 73, 74, 76] linked to [68]; [41, 75] linked to [51]; [30] linked to [37]; [31, 33] linked to [88] [46, 49, 60, 86] linked to [78]	N/A	N/A	N/A	Evidence of an association between maternal ZIKV infection and congenital abnormalities has been shown in epidemiological studies with different designs: • 22 groups of case series, case reports and a cross-sectional study provide evidence in favour of at least one dimension of causality; • 1 cohort study provides evidence for three dimensions of causality, including a potentially strong measure of effect; • 3 groups of ecological/modelling studies with data from different states in Brazil and French Polynesia provide evidence in favour of causality for at least two dimensions of causality.

Abbreviations: CHIKV, chikungunya virus; CMV, cytomegalovirus; CSF, cerebrospinal fluid; DENV, dengue virus; FISH, fluorescence in situ hybridization; HSV, herpes simplex virus; HEK, human embryonic kidney cells; hESC, human embryonic stem cells; hiPSC, human induced pluripotent stem cells; hnPC, human neural progenitor cell; IHC, immunohistochemistry; KO, knockout; mRNA, messenger RNA; miRNA, micro RNA; NSC, neural stem cell; PHT, primary human trophoblasts; qPCR, quantitative polymerase chain reaction; RESP, Registro de Eventos em Saúde Pública; RT-PCR, reverse transcriptase PCR; ROS, reactive oxygen species; RR, risk ratio; SINASC Sistema de Informações de Nascidos Vivos; ZIKV, WNV, West Nile virus; Zika virus.

References

[1-12 not part of this body of evidence]

13. World Health Organization. Zika situation report. Zika virus, Microcephaly and Guillain-Barré syndrome - 31 March 2016. 2016. Available from: http://www.who.int/emergencies/zika-virus/situation-report/31-march-2016/en/. Last accessed 17.08.2016.

[1-12 not part of this body of evidence]

21. Dick GW, Kitchen SF, Haddow AJ. Zika virus. I. Isolations and serological specificity. Trans R Soc Trop Med Hyg. 1952;46(5):509-20. Epub 1952/09/01. PubMed PMID: 12995440.

S4 Table

22. Dick GW. Zika virus. II. Pathogenicity and physical properties. Trans R Soc Trop Med Hyg. 1952;46(5):521-34. Epub 1952/09/01. PubMed PMID: 12995441.

23. Bearcroft WG. Zika virus infection experimentally induced in a human volunteer. Trans R Soc Trop Med Hyg. 1956;50(5):442-8. Epub 1956/09/01. PubMed PMID: 13380987.

24. Reagan RL, Stewart MT, Delaha EC, Brueckner AL. Response of the Syrian hamster to eleven tropical viruses by various routes of exposure. Tex Rep Biol Med. 1954;12(3):524-7. PubMed PMID: 0007368163.

25. Weinbren MP, Williams MC. Zika virus: further isolations in the Zika area, and some studies on the strains isolated. Trans R Soc Trop Med Hyg. 1958;52(3):263-8. Epub 1958/05/01. PubMed PMID: 13556872.

26. Andral L, Bres P, Serie C. Yellow fever in Ethiopia. III. Serological and virological study of the forest fauna. [French]. Bull World Health Org. 1968;38(6):855-61. PubMed PMID: 0008222620.

27. Bell TM, Field EJ, Narang HK. Zika virus infection of the central nervous system of mice. Arch Gesamte Virusforsch. 1971;35(2):183-93. Epub 1971/01/01. PubMed PMID: 5002906.

28. Way JH, Bowen ET, Platt GS. Comparative studies of some African arboviruses in cell culture and in mice. J Gen Virol. 1976;30(1):123-30. Epub 1976/01/01. doi: 10.1099/0022-1317-30-1-123. PubMed PMID: 1245842.

29. Hamel R, Dejarnac O, Wichit S, Ekchariyawat P, Neyret A, Luplertlop N, et al. Biology of Zika Virus Infection in Human Skin Cells. J Virol. 2015;89(17):8880-96. Epub 2015/06/19. doi: 10.1128/JVI.00354-15. PubMed PMID: 26085147; PubMed Central PMCID: PMC4524089.

30. Oliveira Melo AS, Malinger G, Ximenes R, Szejnfeld PO, Alves Sampaio S, Bispo de Filippis AM. Zika virus intrauterine infection causes fetal brain abnormality and microcephaly: tip of the iceberg? Ultrasound Obstet Gynecol. 2016;47(1):6-7. Epub 2016/01/06. doi: 10.1002/uog.15831. PubMed PMID: 26731034.

31. Ventura CV, Maia M, Bravo-Filho V, Gois AL, Belfort R, Jr. Zika virus in Brazil and macular atrophy in a child with microcephaly. Lancet. 2016;387(10015):228. Epub 2016/01/18. doi: 10.1016/S0140-6736(16)00006-4. PubMed PMID: 26775125.

32. Schuler-Faccini L, Ribeiro EM, Feitosa IM, Horovitz DD, Cavalcanti DP, Pessoa A, et al. Possible Association Between Zika Virus Infection and Microcephaly - Brazil, 2015. MMWR Morb Mortal Wkly Rep. 2016;65(3):59-62. Epub 2016/01/29. doi: 10.15585/mmwr.mm6503e2. PubMed PMID: 26820244.

33. Ventura CV, Maia M, Ventura BV, Linden VV, Araujo EB, Ramos RC, et al. Ophthalmological findings in infants with microcephaly and presumable intra-uterus Zika virus infection. Arq Bras Oftalmol. 2016;79(1):1-3. Epub 2016/02/04. doi: 10.5935/0004-2749.20160002. PubMed PMID: 26840156.

34. Mlakar J, Korva M, Tul N, Popovic M, Poljsak-Prijatelj M, Mraz J, et al. Zika Virus Associated with Microcephaly. N Engl J Med. 2016;374(10):951-8. Epub 2016/02/11. doi: 10.1056/NEJMoa1600651. PubMed PMID: 26862926.

35. de Paula Freitas B, de Oliveira Dias JR, Prazeres J, Sacramento GA, Ko AI, Maia M, et al. Ocular Findings in Infants With Microcephaly Associated With Presumed Zika Virus Congenital Infection in Salvador, Brazil. JAMA Ophthalmol. 2016. Epub 2016/02/13. doi: 10.1001/jamaophthalmol.2016.0267. PubMed PMID: 26865554.

36. Martines RB, Bhatnagar J, Keating MK, Silva-Flannery L, Muehlenbachs A, Gary J, et al. Notes from the Field: Evidence of Zika Virus Infection in Brain and Placental Tissues from Two Congenitally Infected Newborns and Two Fetal Losses--Brazil, 2015. MMWR Morb Mortal Wkly Rep. 2016;65(6):159-60. Epub 2016/02/20. doi: 10.15585/mmwr.mm6506e1. PubMed PMID: 26890059.

37. Calvet G, Aguiar RS, Melo AS, Sampaio SA, de Filippis I, Fabri A, et al. Detection and sequencing of Zika virus from amniotic fluid of fetuses with microcephaly in Brazil: a case study. Lancet Infect Dis. 2016;16(6):653-60. Epub 2016/02/22. doi: 10.1016/S1473-3099(16)00095-5. PubMed PMID: 26897108.

38. Sarno M, Sacramento GA, Khouri R, do Rosario MS, Costa F, Archanjo G, et al. Zika Virus Infection and Stillbirths: A Case of Hydrops Fetalis, Hydranencephaly and Fetal Demise. PLoS Negl Trop Dis. 2016;10(2):e0004517. Epub 2016/02/26. doi: 10.1371/journal.pntd.0004517. PubMed PMID: 26914330; PubMed Central PMCID: PMC4767410.

39. Werner H, Fazecas T, Guedes B, Lopes Dos Santos J, Daltro P, Tonni G, et al. Intrauterine Zika virus infection and microcephaly: correlation of perinatal imaging and three-dimensional virtual physical models. Ultrasound Obstet Gynecol. 2016;47(5):657-60. Epub 2016/03/01. doi: 10.1002/uog.15901. PubMed PMID: 26923098.

40. Meaney-Delman D, Hills SL, Williams C, Galang RR, Iyengar P, Hennenfent AK, et al. Zika Virus Infection Among U.S. Pregnant Travelers - August 2015-February 2016. MMWR Morb Mortal Wkly Rep. 2016;65(8):211-4. Epub 2016/03/05. doi: 10.15585/mmwr.mm6508e1. PubMed PMID: 26938703.

41. Jouannic JM, Friszer S, Leparc-Goffart I, Garel C, Eyrolle-Guignot D. Zika virus infection in French Polynesia. Lancet. 2016;387(10023):1051-2. Epub 2016/03/06. doi: 10.1016/S0140-6736(16)00625-5. PubMed PMID: 26944027.

42. Brasil P, Pereira JP, Jr., Raja Gabaglia C, Damasceno L, Wakimoto M, Ribeiro Nogueira RM, et al. Zika Virus Infection in Pregnant Women in Rio de Janeiro - Preliminary Report. N Engl J Med. 2016. Epub 2016/03/05. doi: 10.1056/NEJMoa1602412. PubMed PMID: 26943629.

43. Miranda-Filho Dde B, Martelli CM, Ximenes RA, Araujo TV, Rocha MA, Ramos RC, et al. Initial Description of the Presumed Congenital Zika Syndrome. Am J Public Health. 2016;106(4):598-600. Epub 2016/03/10. doi: 10.2105/AJPH.2016.303115. PubMed PMID: 26959258.

44. Tang H, Hammack C, Ogden SC, Wen Z, Qian X, Li Y, et al. Zika Virus Infects Human Cortical Neural Progenitors and Attenuates Their Growth. Cell Stem Cell. 2016;18(5):587-90. Epub 2016/03/10. doi: 10.1016/j.stem.2016.02.016. PubMed PMID: 26952870.

45. Villamil-Gomez WE, Mendoza-Guete A, Villalobos E, Gonzalez-Arismendy E, Uribe-Garcia AM, Castellanos JE, et al. Diagnosis, management and follow-up of pregnant women with Zika virus infection: A preliminary report of the ZIKERNCOL cohort study on Sincelejo, Colombia. Travel Med Infect Dis. 2016;14(2):155-8. Epub 2016/03/11. doi: 10.1016/j.tmaid.2016.02.004. PubMed PMID: 26960750.

46. Kleber de Oliveira W, Cortez-Escalante J, De Oliveira WT, do Carmo GM, Henriques CM, Coelho GE, et al. Increase in Reported Prevalence of Microcephaly in Infants Born to Women Living in Areas with Confirmed Zika Virus Transmission During the First Trimester of Pregnancy - Brazil, 2015. MMWR Morb Mortal Wkly Rep. 2016;65(9):242-7. Epub 2016/03/11. doi: 10.15585/mmwr.mm6509e2. PubMed PMID: 26963593.

47. Cauchemez S, Besnard M, Bompard P, Dub T, Guillemette-Artur P, Eyrolle-Guignot D, et al. Association between Zika virus and microcephaly in French Polynesia, 2013-15: a retrospective study. Lancet. 2016;387(10033):2125-32. Epub 2016/03/20. doi: 10.1016/S0140-6736(16)00651-6. PubMed PMID: 26993883; PubMed Central PMCID: PMC4909533.

48. Deng YQ, Zhao H, Li XF, Zhang NN, Liu ZY, Jiang T, et al. Isolation, identification and genomic characterization of the Asian lineage Zika virus imported to China. Sci China Life Sci. 2016;59(4):428-30. Epub 2016/03/20. doi: 10.1007/s11427-016-5043-4. PubMed PMID: 26993654.

49. Faria NR, Azevedo Rdo S, Kraemer MU, Souza R, Cunha MS, Hill SC, et al. Zika virus in the Americas: Early epidemiological and genetic findings. Science. 2016;352(6283):345-9. Epub 2016/03/26. doi: 10.1126/science.aaf5036. PubMed PMID: 27013429; PubMed Central PMCID: PMC4918795.

50. Rossi SL, Tesh RB, Azar SR, Muruato AE, Hanley KA, Auguste AJ, et al. Characterization of a Novel Murine Model to Study Zika Virus. Am J Trop Med Hyg. 2016;94(6):1362-9. Epub 2016/03/30. doi: 10.4269/ajtmh.16-0111. PubMed PMID: 27022155; PubMed Central PMCID: PMC4889758.

51. Besnard M, Eyrolle-Guignot D, Guillemette-Artur P, Lastere S, Bost-Bezeaud F, Marcelis L, et al. Congenital cerebral malformations and dysfunction in fetuses and newborns following the 2013 to 2014 Zika virus epidemic in French Polynesia. Euro Surveill. 2016;21(13). Epub 2016/03/31. doi: 10.2807/1560-7917.ES.2016.21.13.30181. PubMed PMID: 27063794.

52. Driggers RW, Ho CY, Korhonen EM, Kuivanen S, Jaaskelainen AJ, Smura T, et al. Zika Virus Infection with Prolonged Maternal Viremia and Fetal Brain Abnormalities. N Engl J Med. 2016;374(22):2142-51. Epub 2016/03/31. doi: 10.1056/NEJMoa1601824. PubMed PMID: 27028667.

S4 Table

53. Dowall SD, Graham VA, Rayner E, Atkinson B, Hall G, Watson RJ, et al. A Susceptible Mouse Model for Zika Virus Infection. PLoS Negl Trop Dis. 2016;10(5):e0004658. doi: 10.1371/journal.pntd.0004658. PubMed PMID: 27149521; PubMed Central PMCID: PMC4858159.

54. Homan J, Malone RW, Darnell SJ, Bremel RD. Antibody mediated epitope mimicry in the pathogenesis of Zika virus related disease. 2016. Available from: http://biorxiv.org/content/biorxiv/early/2016/03/19/044834.full.pdf. Cited 9 November 2016.

55. Pan American Health Organization. Epidemiological Alert. Zika virus infection 24 March 2016. 2016. Available from: http://www.paho.org/hq/index.php?option=com_docman&task=doc_view&Itemid=270&gid=33937&lang=en. Last accessed 17.08.2016.

56. Pan American Health Organization. Epidemiological Alert. Zika virus infection 08 April 2016. 2016. Available from: http://www.paho.org/hq/index.php?option=com_docman&task=doc_view&Itemid=270&gid=34144&lang=en. Last accessed 17.08.2016.

57. Pan American Health Organization. Epidemiological Update. Zika virus infection - 28 April 2016. 2016. Available from: http://www.paho.org/hq/index.php?option=com_docman&task=doc_view&Itemid=270&gid=34327&lang=en. Last accessed 17.08.2016.

58. Pylro V, Oliveira F, Morais D, Orellana S, Pais F, Medeiros J, et al. Exploring miRNAs as the key to understand symptoms induced by ZIKA virus infection through a collaborative database. 2016. Available from: http://biorxiv.org/content/biorxiv/early/2016/03/06/042382.full.pdf. Cited 9 November 2016.

59. Dudley DM, Aliota MT, Mohr EL, Weiler AM, Lehrer-Brey G, Weisgrau KL, et al. Natural history of Asian lineage Zika virus infection in macaques. bioRxiv. 2016. doi: 10.1101/046334.

60. Reefhuis J, Gilboa SM, Johansson MA, Valencia D, Simeone RM, Hills SL, et al. Projecting Month of Birth for At-Risk Infants after Zika Virus Disease Outbreaks. Emerg Infect Dis. 2016;22(5):828-32. doi: 10.3201/eid2205.160290. PubMed PMID: 27088494; PubMed Central PMCID: PMC4861542.

61. European Centre for Disease Prevention and Control. Rapid risk assessment. Zika virus disease epidemic: potential association with microcephaly and Guillain-Barré syndrome. First update 21 January 2016. 2016. Available from: http://ecdc.europa.eu/en/publications/Publications/rapid-risk-assessment-zika-virus-first-update-jan-2016.pdf. Last accessed 17.08.2016.

62. Zmurko J, Marques RE, Schols D, Verbeken E, Kaptein SJF, Neyts J. The viral polymerase inhibitor 7-deaza-2'-C-methyladenosine is a potent inhibitor of in 1 vitro Zika virus replication and delays disease progression in a robust mouse infection model. PLoS Negl Trop Dis. 2016;10(5). doi: 10.1101/041905.

63. Garcez PP, Loiola EC, Madeiro da Costa R, Higa LM, Trindade P, Delvecchio R, et al. Zika virus impairs growth in human neurospheres and brain organoids. Science. 2016;352(6287):816-8. Epub 2016/04/12. doi: 10.1126/science.aaf6116. PubMed PMID: 27064148.

S4 Table

64. Nowakowski TJ, Pollen AA, Di Lullo E, Sandoval-Espinosa C, Bershteyn M, Kriegstein AR. Expression Analysis Highlights AXL as a Candidate Zika Virus Entry Receptor in Neural Stem Cells. Cell Stem Cell. 2016;18(5):591-6. Epub 2016/04/04. doi: 10.1016/j.stem.2016.03.012. PubMed PMID: 27038591; PubMed Central PMCID: PMC4860115.

65. Hazin AN, Poretti A, Turchi Martelli CM, Huisman TA, Microcephaly Epidemic Research G, Di Cavalcanti Souza Cruz D, et al. Computed Tomographic Findings in Microcephaly Associated with Zika Virus. N Engl J Med. 2016;374(22):2193-5. Epub 2016/04/07. doi: 10.1056/NEJMc1603617. PubMed PMID: 27050112.

66. World Health Organization. Zika situation report. Zika virus, Microcephaly and Guillain-Barré syndrome - 07 April 2016. 2016. Available from: http://www.who.int/emergencies/zika-virus/situation-report/7-april-2016/en/. Last accessed 17.08.2016.

67. Pan American Health Organization. Epidemiological Alert. Zika virus infection 31 March 2016. 2016. Available from: http://www.paho.org/hq/index.php?option=com_docman&task=doc_view&Itemid=270&gid=34041&lang=en. Last accessed 17.08.2016.

68. Microcephaly Epidemic Research Group. Microcephaly in Infants, Pernambuco State, Brazil, 2015. Emerg Infect Dis. 2016;22(6):1090-93. doi: 10.3201/eid2206.160062. PubMed Central PMCID: PMC4880105.

69. Campanati L, Higa LM, Delvecchio R, Pezzuto P, Valadão AL, Monteiro FL, et al. The Impact of African and Brazilian ZIKV isolates on neuroprogenitors. 2016. Available from: http://biorxiv.org/content/biorxiv/early/2016/03/31/046599.full.pdf. Cited 9 November 2016.

70. Zika Experimental Science Team. ZIKV-003 and ZIKV-005: Infection with French Polynesian and Asian lineage Zika virus during the first pregnancy trimester 2016 [10.05.2016]. Available from: https://zika.labkey.com/project/OConnor/ZIKV-003/begin.view?

71. Lazear HM, Govero J, Smith AM, Platt DJ, Fernandez E, Miner JJ, et al. A Mouse Model of Zika Virus Pathogenesis. Cell Host Microbe. 2016;19(5):720-30. Epub 2016/04/14. doi: 10.1016/j.chom.2016.03.010. PubMed PMID: 27066744; PubMed Central PMCID: PMC4866885.

72. Bayer A, Lennemann NJ, Ouyang Y, Bramley JC, Morosky S, Marques ET, Jr., et al. Type III Interferons Produced by Human Placental Trophoblasts Confer Protection against Zika Virus Infection. Cell Host Microbe. 2016;19(5):705-12. Epub 2016/04/14. doi: 10.1016/j.chom.2016.03.008. PubMed PMID: 27066743; PubMed Central PMCID: PMC4866896.

73. de Fatima Vasco Aragao M, van der Linden V, Brainer-Lima AM, Coeli RR, Rocha MA, Sobral da Silva P, et al. Clinical features and neuroimaging (CT and MRI) findings in presumed Zika virus related congenital infection and microcephaly: retrospective case series study. BMJ. 2016;353:i1901. Epub 2016/04/15. doi: 10.1136/bmj.i1901. PubMed PMID: 27075009; PubMed Central PMCID: PMC4830901.

74. Cavalheiro S, Lopez A, Serra S, Da Cunha A, da Costa MD, Moron A, et al. Microcephaly and Zika virus: neonatal neuroradiological aspects. Childs Nerv Syst. 2016;32(6):1057-60. Epub 2016/04/16. doi: 10.1007/s00381-016-3074-6. PubMed PMID: 27080092; PubMed Central PMCID: PMC4882355.

75. Guillemette-Artur P, Besnard M, Eyrolle-Guignot D, Jouannic JM, Garel C. Prenatal brain MRI of fetuses with Zika virus infection. Pediatr Radiol. 2016;46(7):1032-9. Epub 2016/04/20. doi: 10.1007/s00247-016-3619-6. PubMed PMID: 27090801.

76. Cordeiro MT, Pena LJ, Brito CA, Gil LH, Marques ET. Positive IgM for Zika virus in the cerebrospinal fluid of 30 neonates with microcephaly in Brazil. Lancet. 2016;387(10030):1811-2. Epub 2016/04/23. doi: 10.1016/S0140-6736(16)30253-7. PubMed PMID: 27103126.

77. Qian X, Nguyen HN, Song MM, Hadiono C, Ogden SC, Hammack C, et al. Brain-Region-Specific Organoids Using Mini-bioreactors for Modeling ZIKV Exposure. Cell. 2016;165(5):1238-54. Epub 2016/04/28. doi: 10.1016/j.cell.2016.04.032. PubMed PMID: 27118425; PubMed Central PMCID: PMC4900885.

78. Paploski IA, Prates AP, Cardoso CW, Kikuti M, Silva MM, Waller LA, et al. Time Lags between Exanthematous Illness Attributed to Zika Virus, Guillain-Barre Syndrome, and Microcephaly, Salvador, Brazil. Emerg Infect Dis. 2016;22(8):1438-44. Epub 2016/05/05. doi: 10.3201/eid2208.160496. PubMed PMID: 27144515.

79. Noronha L, Zanluca C, Azevedo ML, Luz KG, Santos CN. Zika virus damages the human placental barrier and presents marked fetal neurotropism. Mem Inst Oswaldo Cruz. 2016;111(5):287-93. Epub 2016/05/05. doi: 10.1590/0074-02760160085. PubMed PMID: 27143490; PubMed Central PMCID: PMC4878297.

80. Moron AF, Cavalheiro S, Milani H, Sarmento S, Tanuri C, de Souza FF, et al. Microcephaly associated with maternal Zika virus infection. BJOG. 2016;123(8):1265-9. Epub 2016/05/07. doi: 10.1111/1471-0528.14072. PubMed PMID: 27150580.

81. Dang J, Tiwari SK, Lichinchi G, Qin Y, Patil VS, Eroshkin AM, et al. Zika Virus Depletes Neural Progenitors in Human Cerebral Organoids through Activation of the Innate Immune Receptor TLR3. Cell Stem Cell. 2016;19(2):258-65. Epub 2016/05/11. doi: 10.1016/j.stem.2016.04.014. PubMed PMID: 27162029.

82. Wu KY, Zuo GL, Li XF, Ye Q, Deng YQ, Huang XY, et al. Vertical transmission of Zika virus targeting the radial glial cells affects cortex development of offspring mice. Cell Res. 2016;26(6):645-54. Epub 2016/05/14. doi: 10.1038/cr.2016.58. PubMed PMID: 27174054; PubMed Central PMCID: PMC4897185.

83. Li C, Xu D, Ye Q, Hong S, Jiang Y, Liu X, et al. Zika Virus Disrupts Neural Progenitor Development and Leads to Microcephaly in Mice. Cell Stem Cell. 2016;19(1):120-6. Epub 2016/05/18. doi: 10.1016/j.stem.2016.04.017. PubMed PMID: 27179424.

84. Miner JJ, Cao B, Govero J, Smith AM, Fernandez E, Cabrera OH, et al. Zika Virus Infection during Pregnancy in Mice Causes Placental Damage and Fetal Demise. Cell. 2016;165(5):1081-91. Epub 2016/05/18. doi: 10.1016/j.cell.2016.05.008. PubMed PMID: 27180225; PubMed Central PMCID: PMC4874881.

S4 Table

85. Culjat M, Darling SE, Nerurkar VR, Ching N, Kumar M, Min SK, et al. Clinical and Imaging Findings in an Infant With Zika Embryopathy. Clin Infect Dis. 2016. Epub 2016/05/20. doi: 10.1093/cid/ciw324. PubMed PMID: 27193747.

86. Johansson MA, Mier-y-Teran-Romero L, Reefhuis J, Gilboa SM, Hills SL. Zika and the Risk of Microcephaly. N Engl J Med. 2016;375(1):1-4. Epub 2016/05/26. doi: 10.1056/NEJMp1605367. PubMed PMID: 27222919; PubMed Central PMCID: PMC4945401.

87. Aliota MT, Caine EA, Walker EC, Larkin KE, Camacho E, Osorio JE. Characterization of Lethal Zika Virus Infection in AG129 Mice. PLoS Negl Trop Dis. 2016;10(4):e0004682. Epub 2016/05/24. doi: 10.1371/journal.pntd.0004682. PubMed PMID: 27093158; PubMed Central PMCID: PMC4836712.

88. Ventura CV, Maia M, Travassos SB, Martins TT, Patriota F, Nunes ME, et al. Risk Factors Associated With the Ophthalmoscopic Findings Identified in Infants With Presumed Zika Virus Congenital Infection. JAMA Ophthalmol. 2016;134(8):912-8. Epub 2016/05/27. doi: 10.1001/jamaophthalmol.2016.1784. PubMed PMID: 27228275.

89. Miranda HA, 2nd, Costa MC, Frazao MA, Simao N, Franchischini S, Moshfeghi DM. Expanded Spectrum of Congenital Ocular Findings in Microcephaly with Presumed Zika Infection. Ophthalmology. 2016;123(8):1788-94. Epub 2016/05/30. doi: 10.1016/j.ophtha.2016.05.001. PubMed PMID: 27236271.

90. Hanaoka MM, Kanas AF, Braconi CT, Mendes EA, Santos RA, Ferreira LCdS, et al. A Zika virus-associated microcephaly case with background exposure to STORCH agents. 2016. Available from: http://biorxiv.org/content/early/2016/05/10/052340; files/1514/052340.html. Cited 9 November 2016.

91. Garcez PP, Nascimento JM, Mota de Vasconcelos J, Madeiro da Costa R, Delvecchio R, Trindade P, et al. Combined proteome and transcriptome analyses reveal that Zika virus circulating in Brazil alters cell cycle and neurogenic programmes in human neurospheres. 2016. Available from: https://peerj.com/preprints/2033/. Cited 9 November 2016.

92. Ganguly B, Ganguly E. Disruption of human astn2 function by ZIKV ns4b gene as a molecular basis for Zika viral microcephaly. 2016. Available from: http://biorxiv.org/content/early/2016/05/20/054486; files/1499/054486.html. Cited 9 November 2016.

93. Frumence E, Roche M, Krejbich-Trotot P, El-Kalamouni C, Nativel B, Rondeau P, et al. The South Pacific epidemic strain of Zika virus replicates efficiently in human epithelial A549 cells leading to IFN-beta production and apoptosis induction. Virology. 2016;493:217-26. doi: 10.1016/j.virol.2016.03.006. PubMed PMID: 27060565.

[94-96 not part of this body of evidence]

97. O'Leary DR, Kuhn S, Kniss KL, Hinckley AF, Rasmussen SA, Pape WJ, et al. Birth outcomes following West Nile Virus infection of pregnant women in the United States: 2003-2004. Pediatrics. 2006;117(3):e537-45. doi: 10.1542/peds.2005-2024. PubMed PMID: 16510632.

S4 Table

98. Pouliot SH, Xiong X, Harville E, Paz-Soldan V, Tomashek KM, Breart G, et al. Maternal dengue and pregnancy outcomes: a systematic review. Obstet Gynecol Surv. 2010;65(2):107-18. doi: 10.1097/OGX.0b013e3181cb8fbc. PubMed PMID: 20100360.
