# Supplementary material for: Zika Virus Infection as a Cause of Congenital Brain Abnormalities and Guillain–Barré Syndrome: Systematic Review
Source: PLoS Med. 2017 Jan 3;14(1):e1002203. doi: 10.1371/journal.pmed.1002203 (PMC5207634; doi:10.1371/journal.pmed.1002203)
Supplement: S5 Table — (PDF) [file pmed.1002203.s008.pdf]

## S5 Table

## S5 Table. Assessment of quality of the body of evidence about Zika virus infection and congenital brain abnormalities

| **Domain[a]** | **Assessment** |
|---|---|
| Evidence reviewed: 9 case reports, 22 case series, 1 cohort study, 2 cross-sectional studies (1 in animals); 2 modelling studies, 5 ecological studies, 18 animal experiments, 10 in vitro experiments, 3 sequence analysis study | |
| Risk of bias | Human studies: Uncontrolled studies and ecological studies formed the majority of the evidence for assessing temporality. Within this group four studies showed laboratory evidence of ZIKV infection before detection of congenital abnormalities. We found risks of bias in two comparative studies. The cohort study was at risk of performance bias because of differences in follow up between exposed and unexposed groups. The modelling study was at risk of measurement bias because exposure to Zika virus infection was not measured at the individual level and there was no adjustment for confounding.<br>Animal studies: We did not assess risk of bias formally because almost all studies were from the 1950s and 1970s and lacked sufficient details for assessment. |
| Imprecision | Assessed for in comparative studies only. The modelling study that estimated a risk ratio, confidence intervals were extremely wide. The cohort study did not estimate an effect measure but the numbers of study participants was small, so confidence intervals would be wide. |
| Inconsistency | See S4 Table, dimension 10 (consistency) |
| Publication bias | Could not be assessed formally. Expert panel was not aware of studies that we missed. Our search strategy identified only one case report in which findings addressing one aspect of biological plausibility were not consistent with causality. |
| Indirectness | Animal studies provide indirect evidence because findings cannot be extrapolated to humans: in particular, studies using African lineage ZIKV used virus strains that had undergone multiple passages and animals that were particularly vulnerable to central nervous system lesions. |
| Magnitude of effect | See S4 Table, dimension 5 (strength of association) |
| Opposing plausible residual bias and confounding | None identified |
| Dose effect | See S4 Table, dimension 4 (dose-response relationship) |

[a.] Domains from GRADE working group, assessed as suggested for urgent situations (reference 20 in main text) [1]. Some of these overlap with causality dimensions so the assessment was not repeated.

## References

1. Thayer KA, Schunemann HJ. Using GRADE to respond to health questions with different levels of urgency. Environ Int. 2016;92-93:585-9. doi: 10.1016/j.envint.2016.03.027. PubMed PMID: 27126781.
