# Supplementary material for: Zika Virus Infection as a Cause of Congenital Brain Abnormalities and Guillain–Barré Syndrome: Systematic Review
Source: PLoS Med. 2017 Jan 3;14(1):e1002203. doi: 10.1371/journal.pmed.1002203 (PMC5207634; doi:10.1371/journal.pmed.1002203)
Supplement: S6 Table — (PDF) [file pmed.1002203.s009.pdf]

S6 Table. Causality framework evidence for Zika virus infection and Guillain-Barré syndrome

Contents

S6 Table. Causality framework evidence for Zika virus infection and Guillain-Barré syndrome 1

1. Temporality 2
2. Biological plausibility 4
3. Strength of association 6
4. Exclusion of alternative explanations 6
5. Cessation 7
6. Dose-response relationship 8
7. Animal experiments 8
8. Analogy 9
9. Specificity 11
10. Consistency 11

References 13

S6 Table

Question no.	Causality dimension and question	Study type, number of primary items [reference] of included items	Linked items	Country of study	Support causality dimension	Do not support causality dimension	Summary of evidence
1. Temporality		Summary: Total, 31 studies in 26 groups. Reviewer assessments found sufficient evidence for all 3 questions of an appropriate temporal relationship between Zika virus (ZIKV) infection and Guillain-Barré syndrome (GBS). The time interval between ZIKV symptoms and onset of neurological symptoms was compatible with that of other accepted triggers of GBS.					
1.1a	Does ZIKV infection precede the development of GBS in individuals?	Case report, 4 [110, 111, 117, 120];<br>Case series, 4 [105, 106, 108];<br>Case-control study, 1 [112]	[121] linked to [120]<br>[102, 116] linked to [112]	Brazil,<br>El Salvador, Panama, Puerto Rico, Venezuela, French Polynesia, United States[a], Netherlands[a]	9	0	The exposure was reported before the outcome in studies in Brazil, El Salvador, Panama, Puerto Rico, Venezuela, in French Polynesia, and in returning travellers:<br>• The first case was reported during the 2013-14 ZIKV outbreak in French Polynesia. An influenza-like illness occurred 7 days before the onset of GBS symptoms. ZIKV RT-PCR was negative on day 8. Serology on days 8 and 28 suggested recent ZIKV infection (IgM positive) and past dengue virus (DENV) infection (IgG positive) [102];<br>• 83 of 112 patients (74%) with GBS reported ZIKV-related symptoms before the onset of GBS symptoms, of which 53 cases (all except 42 cases in Brazil and all cases in El Salvador) were laboratory confirmed ZIKV infections [105, 106, 108, 110-112, 120].
1.1b	Is there a consistent time-dependent relationship between the occurrence of ZIKV cases and cases with GBS at population-level?	Ecological study/outbreak report, 16 [55-57, 67, 100, 104, 107, 109, 115]	[103, 122] linked to [104]	Brazil, Colombia, Dominican Republic,<br>El Salvador, French Guiana, Haiti, Honduras, Martinique, Panama, Paraguay, Puerto Rico, Suriname, Venezuela, Micronesia,	9	7	Studies at the population level report differing findings about the temporal relationship between reported cases of Zika-like illness and GBS cases:<br>• In 9 countries (Brazil, Colombia, Dominican Republic, El Salvador, French Polynesia, Honduras, Paraguay, Suriname and Venezuela) an increase of GBS cases was observed during the ZIKV epidemic [56, 57, 67, 104, 107, 109]. In Brazil, the number of cases recorded in 2015 was 19% higher than in the previous year [57];<br>• In Haiti, French Guiana, Federal States of Micronesia (Yap), Panama, Puerto Rico, and Martinique there

				French Polynesia, Pacific Islands			was no increase in GBS cases above the background level [55, 57, 67, 109] or no increase in hospitalisations during the epidemic [100]<br>• 1 study examined reports of acute flaccid paralysis (AFP) collected for polio surveillance in children in several Pacific Islands as a possible signal of GBS cases [115]. An increase above the expected annual number of AFP cases in the same year as a ZIKV outbreak was only seen in the Solomon Islands. In most islands the expected number of cases was <1 per year, however.
1.2	Is the interval between exposure to ZIKV and occurrence of symptoms typical for para- or post-infectious GBS?	Case report, 3 [110, 111, 117]; Case series, 2 [106, 108]; Case-control study, 1 [112]; Ecological study/outbreak report: 1 [78]	[102, 116] linked to [112] [102, 116] linked to[112]	Brazil, El Salvador, Panama, Puerto Rico, Venezuela, French Polynesia	7	0	Data from patients in several countries suggest a time interval between ZIKV symptoms and the onset of GBS symptoms that is consistent with observations for *Campylobacter jejuni* associated GBS (median 9 days, range 1-26 days) [124, 126, 127]:<br>• 10 days for 2 cases in Venezuela [111] and Panama [106, 108];<br>• Median 6 days (IQR 4-10) for 37 patients in a case control study in French Polynesia [112];<br>• 3 days for 1 case in Puerto Rico [110];<br>• 6 days for 1 Dutch traveller returning from Suriname [120];<br>• 12 days for 1 case with laboratory confirmed ZIKV infection in Brazil [117];<br>• 7-15 days for 12 of 22 patients without laboratory confirmed ZIKV infection in a case series in El Salvador [108]<br>• Lagged time-series cross-correlations from Salvador, Brazil showed a strong positive ecological association between epidemic curves of Zika cases and cases with GBS with a time lag of 5-9 weeks. GBS cases were recorded on the date of hospitalisation rather than the date when symptoms began, so the authors suggest that the time lag between Zika and GBS

							might be shorter, as observed in other outbreaks [78].
2. Biological plausibility		Summary: Total, 6 items in 4 groups reviewed. Reviewer assessments found sufficient evidence for 2 of 3 questions about biologically plausible mechanisms by which ZIKV could trigger the immune-mediated pathology of GBS. There is evidence that supports a role for molecular mimicry, a proposed mechanism of autoimmunity in the development of GBS, which has been reported in *Campylobacter jejuni*-associated GBS. Direct neurotropic effects of ZIKV might also occur.					
2.1	Do ZIKV epitopes mimic host antigens (molecular mimicry)?	Case-control study, 1 [112]; Sequence analysis and phylogenetics, 2 [54, 114]	[102, 116] linked to [112]	French Polynesia	3	0	Evidence for molecular mimicry comes from 2 *in silico* approaches and 1 clinical study:<br>• Analysis of peptide sharing between the ZIKV polyprotein and human proteins related to myelin (de-myelination or axonal neuropathy) and human autoantigens known to be involved in GBS identified 99 candidate proteins with epitope sharing, of which many have been shown to be involved in autoimmune disease [114];<br>• The prediction of B-cell epitopes of ZIKV and the comparison to B-cell epitopes in the human proteome identified several candidate proteins for both a Brazilian (Asian lineage) and African ZIKV strain. The proteins with the highest probability were Optineurin, Synaptogyrin-1, von Willebrand factor A, Pro-neuropeptide Y, Neuron Navigator Y (Brazilian strain only). 3D modelling of the ZIKV Brazil polyprotein showed matches for Pro-neuropeptide Y, Synaptogyrin, Neurotrophin 4, neural cell adhesion molecule, and von Willebrand factor A for a ZIKV region adjacent to high affinity MHC II binding site suggesting B and T-cell co-presentation. These findings suggest that antibodies against ZIKV may also target host proteins involved in neurologic and haemostatic processes [54];<br>• IgM/IgG containing serum samples from GBS patients in French Polynesia were evaluated for reactivity to glycolipids using ELISA and combination microarray screening. 13 (31%) of GBS patients showed positive reactivity to glycolipids at admission and 10 (24%)

							had equivocal results using ELISA. 17 showed reactivity (8 positive, 9 equivocal) towards GA1 either as a single glycolipid or complex. In the microarray most sera showed no or low level binding activity to glycolipid complexes except for antibody binding affinities to GA1–sulphatide complex (N=19, 46%). After three months, the proportion of reactive sera was increased compared to admission: GM11 (26%), GA1 (32%), GD1a (29%), GD1b (29%) or any (48%). No serum showed reactivity with GQ1b. Complementary analysis showed no binding competition between GA1 and ZIKV. The authors suggest that molecular mimicry of GA1 might not be involved in GBS development after ZIKV infection, but the reactivity has also been observed for other glycolipids in individual patients, supporting a molecular mimicry mechanism of disease [112].
2.2	Does ZIKV infection lead to an increase in detectable autoreactive immune cells or autoreactive antibodies?	Case report, 1 [111]		Venezuela	1	0	A pregnant woman in Venezuela developed a rash followed by ascending paralysis suggestive of GBS. Anti-ganglioside GQ1b antibodies and ZIKV antibodies (type not known) were detected 10 days later. She delivered a healthy baby several weeks later [111].
2.3	Are there other biologically plausible mechanisms by which ZIKV could lead to GBS?	None	None	N/A	0	0	No relevant studies identified. A number of experimental studies with human neural stem cells and various mouse models have shown some evidence for neurotropism of ZIKV (see evidence table for congenital abnormalities S4a). A direct, cytotoxic effect of ZIKV, resulting in paralysis, might also be possible. The clinical presentations that are compatible with GBS and the findings of auto-antibodies in some of the Zika-related GBS cases suggest an indirect, immune-mediated mechanism. This review does not provide sufficient evidence to address potential direct effects of ZIKV on the nervous system.

S6 Table

3. Strength of association		Summary: Total 7 items in 2 groups reviewed. The reviewers assessed evidence from the ZIKV outbreak in French Polynesia as showing a strong association between ZIKV and GBS at both the individual and population level. Surveillance reports from Brazil also support an association at the population level.					
3.1	How strong is the association between ZIKV infection and GBS at the individual level?	Case-control study, 1 [112]	[102, 116] linked to [112] 1	French Polynesia	1	0	1 case-control study with 42 GBS cases and 98 using age-, sex-, and residence-matched hospital controls. A crude OR of 59.7 (95% CI 10.4-∞) was estimated for GBS cases being ZIKV IgM or IgG positive compared to controls and an OR of 34.1 (95% CI 5.9-∞) for GBS cases being ZIKV plaque reduction neutralisation test (PRNT) positive compared with controls [112].
3.2	How strong is the association between ZIKV infection and GBS at the population level?	Ecological study/outbreak report: 2 [101] [122]	[103, 104] linked to [122]	Brazil, French Polynesia	2	0	The same 42 cases of GBS in French Polynesia were analysed with a population denominator (all items about the French Polynesia outbreak are considered as part of a single group). The incidence of GBS during the epidemic (0.41 per 1000 person years (py, 95% CI 0.29-0.5) 5in French Polynesia was 21 times higher than in the pre-Zika period of 2009 to 2012 vs. 0.02 per 1000 py, 95% CI 0.01 to 0.03). The authors calculated an attributable risk of 0.39 per 1000 py [104]. Same case data as above, population-based analysis.<br>Hospital-based surveillance data from Brazil showed 1708 reports of GBS from January to November in 2015, a 19% increase compared with 2014 [101]. Several states reported increases in 2015 vs. 2014, e.g. Alagoas (516.7%), Bahia (196.1%), Rio Grande do Norte (108.7%), Piauí (108.3%), Espirito Santo (78.6%), and Rio de Janeiro (60.9%); others reported stable or lower numbers.
4. Exclusion of alternative explanations		Summary: Total, 10 items in 7 groups studies reviewed. Reviewer assessments found sufficient evidence at the individual level that other infectious triggers of GBS have been excluded; no other single infection could have accounted for clusters of GBS. The evidence about other exposures could not be assessed because of an absence of relevant studies					
4.1	Have other explanations/confounders of the association between ZIKV infection and GBS been	Case report, 6 [110, 113, 117-120]; Case-control study, 1 [112]	[121] linked to [120] [102, 116]	Brazil, Haiti, Martinique, Netherlands[a], Puerto Rico, French Polynesia	7	0	Some studies in Brazil, French Polynesia, Puerto Rico, Martinique, Haiti and a traveller returning from Suriname to the Netherlands excluded other acute infections:

	excluded, such as other infections?		linked to [112]				• 5 studies excluded acute dengue virus (DENV) infection [110, 112, 113, 117, 118] by IgM serology, DENV antigens and/or DENV RT-PCR in 48 individuals. However, 44 of 46 tested cases were IgG positive for DENV (see 'co-factors');<br>• 5 studies excluded acute chikungunya (CHIKV) infection GBS by IgM serology and/or CHIKV RT-PCR in 6 individuals [110, 113, 117, 118, 120];<br>• Only 2 studies (including 1 case-control) excluded infection with *C. jejuni*, *Mycoplasma pneumoniae*, Epstein-Barr virus (EBV) and herpes simplex virus (HSV) in 44 individuals [112, 113].
4.2	Have other explanations/confounders of the association between ZIKV infection and GBS been excluded, such as vaccines?				0	0	No relevant studies identified
4.3	Have other explanations/confounders of the association between ZIKV infection and GBS been excluded, such as underlying systemic disease?				0	0	No relevant studies identified
4.4	Have other explanations/confounders of the association between ZIKV infection and GBS been excluded, such as concomitant medication, drugs or other chemicals?				0	0	No relevant studies identified
5. Cessation		Summary: Total 8 items in 6 groups reviewed. Reviewer assessments found sufficient evidence for 1 of 3 questions. In one state in Brazil, four other countries in the Americas and in French Polynesia, reports of GBS decreased after ZIKV transmission ceased. Evidence for the other questions could not be assessed because no relevant studies were identified.					
5.1	Does the intentional prevention/removal/elimination of ZIKV infection in individuals,				0	0	No relevant studies identified

	e.g. by insect repellents, lead to a reduction in cases with GBS?						
5.2	Does the intentional removal/elimination/prevention of ZIKV at population-level, e.g. by vector control, lead to a reduction in cases with GBS?				0	0	No relevant studies identified
5.3	Does a natural removal/elimination/prevention of ZIKV at population-level, e.g. increase in immune individuals or decrease in vector abundance lead to a reduction in cases with GBS?	Ecological study/outbreak report, 6 [56, 57, 78, 104, 109]	[103, 122] linked to [104]	Brazil, Colombia, El Salvador, Honduras, Suriname, French Polynesia	6	0	At population level, a decrease in GBS cases was observed following a decrease in reported cases of Zika-like illness in French Polynesia [104], Brazil (Bahia State) [78], Colombia [56], El Salvador [109], Honduras [109] and Suriname [57]
6. Dose-response relationship		Summary: No relevant studies identified					
6.1	Are the risk and the clinical severity of GBS associated with viral titres or viral load in the urine?	None	N/A	N/A	0	0	No relevant studies identified
7. Animal experiments		Summary: No relevant studies of animal models of immune-mediated neuropathology identified. Evidence about neurotropism of ZIKV summarised in S4 Table.					
7.1	Does inoculation of animals with ZIKV lead to an autoimmune reaction resulting in peripheral neuropathy?	None	N/A	N/A	0	0	No relevant studies identified
7.2	Do other animal experiments support the association of ZIKV infection and GBS?	None	N/A	N/A	0	0	A number of experimental studies with various mouse models have shown some evidence for a neurotropism of ZIKV (see evidence table for congenital abnormalities S4 Table).<br>This question was intended to look for evidence about autoimmune mechanisms. Auto-antibodies have been found in Zika-related GBS cases (see dimension 2, biological plausibility).<br>We did not find any studies of animal models of autoimmunity and ZIKV.

S6 Table

8. Analogy		Summary: Selected studies reviewed for 2 of 3 questions. Analogous mosquito-borne neurotropic flavivirus infections have been reported in association with GBS (West Nile virus, WNV; DENV; Japanese encephalitis virus, JEV). WNV and JEV have also been reported to be associated with direct neurotropic effects and poliomyelitis-like acute flaccid paralysis. The time lag between ZIKV symptoms and GBS symptoms is analogous to intervals reported for other infectious triggers of GBS. There is some evidence that, as for *C. jejuni*-associated GBS, molecular mimicry could be involved. Evidence was not reviewed systematically.					
8.1	Do other flaviviruses or arboviruses cause GBS and by which mechanism(s)?	Studies not found through systematic searches	N/A	N/A	N/A	N/A	Evidence about GBS following mosquito-borne flavivirus infections (WNV, DENV, JEV).<br>• WNV: A population-based study identified cases of acute paralysis following an outbreak of WNV in Colorado, USA in 2003 in an area of 724,000 residents. Of 32 cases with acute paralysis and WNV infection confirmed by serum IgM, only 6 (19%) had paralysis alone whereas the others had concomitant encephalitis or meningitis. 27 cases (84%) had asymmetric weakness consistent with poliomyelitis-like syndrome (19 with cranial nerve involvement), 4 (13%) had symmetric ascending weakness with sensory abnormalities consistent with GBS and 1 had thoracic nerve paralysis. 26 patients (81%) reported no underlying morbidity. In the cases with poliomyelitis-like paralysis, the median latency between WNV infection symptoms and onset of paralysis was 3 days (range 0-18 days) and electromyographic studies suggested involvement of the anterior horn cells. The electromyographic findings in the GBS patients were indicative of demyelinating sensorimotor neuropathy [129]. In an *in vitro* experiment the infection of rat Schwann cells with WNV led to an upregulation of MHC class I and II molecules suggesting that Schwann cells may act as antigen presenting cells and bind to immune cells leading to peripheral nerve cell damage [135].<br>• A narrative review identified 247 studies about neurological complications of DENV infection, of

							which 57 studies were related to immune-mediated complications including 13 studies about GBS. The average latency between illness and onset of weakness was one week [128].<br>• GBS [130, 131] as well as poliomyelitis-type acute flaccid paralysis [133, 134] have also been reported as sequelae of JEV. Mice experimentally infected with JEV showed hindlimb paralysis and demyelination as well as production of antibodies against myelin basic protein (MBP) and proliferation of MBP-specific T cells suggesting a pivotal role of autoimmunity in JEV pathogenesis [138].<br>• A review of vaccine adverse events reports found 6 cases of GBS as suspected side effects 7-27 days after live attenuated 17D-204 yellow fever vaccination in combination with other vaccinations [132].
8.2	Do other pathogens cause GBS and by which mechanism(s)?	Studies not found through systematic searches	N/A	N/A	N/A	N/A	• *C. jejuni* is one of the pathogens commonly associated with GBS. It is estimated that *C. jejuni* precedes GBS in 10-30% of all cases, but only one in every 2000-5000 cases of *C. jejuni* infection will develop GBS [124, 126]. The median latency between symptoms of *C. jejuni* infection and onset of GBS symptoms is reported as 9 days (range 1-23 days) [123, 124, 126, 127]. Several laboratory experiments have provided some evidence for molecular mimicry of human nervous system proteins and pathogen proteins. Potential human candidate proteins for sequence homology are gangliosides and heat shock proteins (HSP) (summarised in [136]).<br>• *M. pneumoniae* has also been associated with GBS. Antibodies against galactocerebroside isolated from GBS patients have been found to cross-react with multiple types of *M. pneumoniae* [137].
8.3	Which pathogen or host factors facilitate the development of GBS?	None	N/A	N/A	N/A	N/A	No relevant studies identified

S6 Table

9. Specificity		Summary: 0 items reviewed. No relevant studies identified.					
9.1	Are there pathological findings in cases with GBS that are specific for ZIKV infection?	None	N/A	N/A	0	0	No relevant studies identified
10. Consistency		Summary: For 3 of 4 questions, there was sufficient evidence of consistency. By geographical region, ZIKV transmission has been associated with the occurrence of GBS in 2 of 3 regions where ZIKV has circulated since 2007. By study design, the association between ZIKV infection and GBS has been found in studies at both individual and population level and with both retrospective and prospective designs. By population group, ZIKV infection has been linked to GBS in both residents of an affected country and travellers from non-affected countries whose only possible exposure to ZIKV was having travelled to an affected country. The evidence according to ZIKV lineage is unclear because an association between ZIKV and GBS has only been reported from countries with ZIKV of the Asian lineage since 2013.					
10.1	Is the association between ZIKV infection and cases with GBS consistently found across different geographical regions?		None	N/A	N/A	N/A	Findings across regions are partly consistent:<br>• Reports of GBS in association with ZIKV transmission have been made in two regions with ZIKV transmission since 2007: Pacific (French Polynesia) and the Americas (Brazil, Colombia, Dominican Republic, El Salvador, Honduras, Paraguay, Surinam, Venezuela) and in travellers returning from the Americas. In some of these countries clusters of cases have occurred but in others isolated individual cases without a population-level increase have been reported.<br>• Cross-sectional studies from different countries in Africa and Asia from 1945-2014 showed ZIKV seroprevalence of 0.6-64% in African countries and 2.3-75% in Asian countries [99] suggesting that ZIKV might have been endemic in several countries for many years. No reports identified that indicated increased numbers of cases of GBS above baseline levels in these countries. Possible explanations include small case numbers or population size, poor surveillance, high levels of immunity to ZIKV, or a lack of an association.
10.2	Is the association between ZIKV infection and cases with GBS consistently found across		None	N/A	N/A	N/A	• The fact that we observe GBS both in travellers to as well as residents of ZIKV affected countries shows that the association can be made across populations

	different populations/subpopulations?						with a possibly different lifetime exposure to other infectious or other agents possibly leading to GBS [108, 120]<br>• No relevant studies found to have examined the association in different subpopulations within a country, e.g. by ethnic group.
10.3	Is the association between ZIKV infection and cases with GBS consistently found across different ZIKV lineages/strains?		None	N/A	N/A	N/A	The data on differential pathogenicity by ZIKV strain or lineage is inconsistent to date:<br>• Several countries in Africa, where the African lineage ZIKV is presumably endemic have not reported GBS as a complication of ZIKV infection. Differences in the sensitivity of surveillance systems between countries that have and have not reported cases have not been examined.<br>• An increase in reports of GBS has only been reported from countries in which Asian lineage ZIKV transmission has been found for the first time. These differences may be true or confounded by differential surveillance and/or population sizes. This hypothesis cannot be supported nor rejected to date.
10.4	Is the association between ZIKV infection and cases with GBS consistently found across different study designs?		None	N/A	N/A	N/A	Evidence of an association between ZIKV infection and GBS has been shown in different epidemiological study designs:<br>• 11 case series or case reports provide evidence in favour of causality for at least one dimension of causality;<br>• 1 case-control study provides evidence in 3 dimensions of causality;<br>• Ecological studies in 9 countries found evidence of an increase in GBS at the same time as ZIKV transmission but 7 did not.

Abbreviations: AFP, acute flaccid paralysis; CHIKV, chikungunya virus; CMV, cytomegalovirus; DENV, dengue virus; HSV, herpes simplex virus; hiPSC, human induced pluripotent stem cells; hnPC, human neural progenitor cell; IHC, immunohistochemistry; JEV, Japanese encephalitis virus; miRNA, micro RNA; NSC, neuronal stem cell; PRNT, plaque reduction neutralisation test; qPCR, quantitative PCR; RT-PCR, reverse transcriptase PCR, RR, risk ratio; WNV; West Nile virus; ZIKV, Zika virus.

S6 Table

References

[1-53 not part of this body of evidence]

54. Homan J, Malone RW, Darnell SJ, Bremel RD. Antibody mediated epitope mimicry in the pathogenesis of Zika virus related disease. 2016. Available from: http://biorxiv.org/content/biorxiv/early/2016/03/19/044834.full.pdf. Cited 9 November 2016.

55. Pan American Health Organization. Epidemiological Alert. Zika virus infection 24 March 2016. 2016. Available from: http://www.paho.org/hq/index.php?option=com_docman&task=doc_view&Itemid=270&gid=33937&lang=en. Last accessed 17.08.2016.

56. Pan American Health Organization. Epidemiological Alert. Zika virus infection 08 April 2016. 2016. Available from: http://www.paho.org/hq/index.php?option=com_docman&task=doc_view&Itemid=270&gid=34144&lang=en. Last accessed 17.08.2016.

57. Pan American Health Organization. Epidemiological Update. Zika virus infection - 28 April 2016. 2016. Available from: http://www.paho.org/hq/index.php?option=com_docman&task=doc_view&Itemid=270&gid=34327&lang=en. Last accessed 17.08.2016.

[58-66 not part of this body of evidence]

67. Pan American Health Organization. Epidemiological Alert. Zika virus infection 31 March 2016. 2016. Available from: http://www.paho.org/hq/index.php?option=com_docman&task=doc_view&Itemid=270&gid=34041&lang=en. Last accessed 17.08.2016.

[68-77 not part of this body of evidence]

78. Paploski IA, Prates AP, Cardoso CW, Kikuti M, Silva MM, Waller LA, et al. Time Lags between Exanthematous Illness Attributed to Zika Virus, Guillain-Barre Syndrome, and Microcephaly, Salvador, Brazil. Emerg Infect Dis. 2016;22(8):1438-44. Epub 2016/05/05. doi: 10.3201/eid2208.160496. PubMed PMID: 27144515.

[79-99 not part of this body of evidence]

100. Duffy MR, Chen TH, Hancock WT, Powers AM, Kool JL, Lanciotti RS, et al. Zika virus outbreak on Yap Island, Federated States of Micronesia. N Engl J Med. 2009;360(24):2536-43. Epub 2009/06/12. doi: 10.1056/NEJMoa0805715. PubMed PMID: 19516034.

101. World Health Organization WHO. Zika situation report. Neurological syndrome and congenital anomalies - 5 February 2016. 2016. Available from: http://apps.who.int/iris/bitstream/10665/204348/1/zikasitrep_5Feb2016_eng.pdf?ua=1. Last accessed 26.10.2016.

102. Oehler E, Watrin L, Larre P, Leparc-Goffart I, Lastere S, Valour F, et al. Zika virus infection complicated by Guillain-Barre syndrome--case report, French Polynesia, December 2013. Euro Surveill. 2014;19(9). Epub 2014/03/15. PubMed PMID: 24626205.

103. Institut de veille sanitaire. Virus Zika Polynésie 2013-2014, Ile de Yap, Micronésie 2007 - Janvier 2014. 2014. Available from: http://www.invs.sante.fr/Publications-et-outils/Points-epidemiologiques/Tous-les-numeros/International/Virus-Zika-en-Polynesie-2013-2014-et-ile-de-Yap-Micronesie-2007-Janvier-2014. Last accessed 17.8.16.

104. Ioos S, Mallet HP, Leparc Goffart I, Gauthier V, Cardoso T, Herida M. Current Zika virus epidemiology and recent epidemics. Med Mal Infect. 2014;44(7):302-7. Epub 2014/07/09. doi: 10.1016/j.medmal.2014.04.008. PubMed PMID: 25001879.

105. Pan American Health Organization. Epidemiological Update. Neurological syndrome, congenital anomalies, and Zika virus infection - 17 January 2016. 2016. Available from: http://www.paho.org/hq/index.php?option=com_docman&task=doc_view&Itemid=270&gid=32879&lang=en. Last accessed 17.08.2016.

106. Pan American Health Organization. Epidemiological Alert. Zika virus infection -3 March 2016. 2016. Available from: http://www.paho.org/hq/index.php?option=com_docman&task=doc_view&Itemid=270&gid=33486&lang=en. Last accessed 17.08.2016.

107. Pan American Health Organization. Epidemiological Alert. Zika virus infection -10 March 2016. 2016. Available from: http://www.paho.org/hq/index.php?option=com_docman&task=doc_view&Itemid=270&gid=33659&lang=en. Last accessed 17.08.2016.

108. Pan American Health Organization. Epidemiological Alert. Zika virus infection -17 March 2016. 2016. Available from: http://www.paho.org/hq/index.php?option=com_docman&task=doc_view&Itemid=270&gid=33768&lang=en. Last accessed 16.08.2016.

109. Pan American Health Organization. Epidemiological Update. Zika virus infection - 21 April 2016. 2016. Available from: http://www.paho.org/hq/index.php?option=com_docman&task=doc_view&Itemid=270&gid=34243&lang=en. Last accessed 17.08.2016.

110. Thomas DL, Sharp TM, Torres J, Armstrong PA, Munoz-Jordan J, Ryff KR, et al. Local Transmission of Zika Virus--Puerto Rico, November 23, 2015-January 28, 2016. MMWR Morb Mortal Wkly Rep. 2016;65(6):154-8. Epub 2016/02/20. doi: 10.15585/mmwr.mm6506e2. PubMed PMID: 26890470.

111. Reyna-Villasmil E, Lopez-Sanchez G, Santos-Bolivar J. [Guillain-Barre syndrome due to Zika virus during pregnancy]. Med Clin (Barc). 2016;146(7):331-2. Epub 2016/03/08. doi: 10.1016/j.medcli.2016.02.002. PubMed PMID: 26947168.

112. Cao-Lormeau VM, Blake A, Mons S, Lastere S, Roche C, Vanhomwegen J, et al. Guillain-Barre Syndrome outbreak associated with Zika virus infection in French Polynesia: a case-control study. Lancet. 2016;387(10027):1531-9. Epub 2016/03/08. doi: 10.1016/S0140-6736(16)00562-6. PubMed PMID: 26948433.

113. Roze B, Najioullah F, Ferge JL, Apetse K, Brouste Y, Cesaire R, et al. Zika virus detection in urine from patients with Guillain-Barre syndrome on Martinique, January 2016. Euro Surveill. 2016;21(9). Epub 2016/03/12. doi: 10.2807/1560-7917.ES.2016.21.9.30154. PubMed PMID: 26967758.

114. Lucchese G, Kanduc D. Zika virus and autoimmunity: From microcephaly to Guillain-Barre syndrome, and beyond. Autoimmun Rev. 2016;15(8):801-8. Epub 2016/03/29. doi: 10.1016/j.autrev.2016.03.020. PubMed PMID: 27019049.

115. Craig AT, Butler MT, Pastore R, Paterson BJ, Durrheim DN. Update on Zika virus transmission in the Pacific islands, 2007 to February 2016 and failure of acute flaccid paralysis surveillance to signal Zika emergence in this setting. Bull World Health Organ. 2016. doi: 10.2471/blt.16.171892.

116. Watrin L, Ghawche F, Larre P, Neau JP, Mathis S, Fournier E. Guillain-Barre Syndrome (42 Cases) Occurring During a Zika Virus Outbreak in French Polynesia. Medicine (Baltimore). 2016;95(14):e3257. Epub 2016/04/09. doi: 10.1097/MD.0000000000003257. PubMed PMID: 27057874.

117. Fontes CA, Dos Santos AA, Marchiori E. Magnetic resonance imaging findings in Guillain-Barre syndrome caused by Zika virus infection. Neuroradiology. 2016;58(8):837-8. Epub 2016/04/14. doi: 10.1007/s00234-016-1687-9. PubMed PMID: 27067205.

118. Brasil P, Sequeira PC, Freitas AD, Zogbi HE, Calvet GA, de Souza RV, et al. Guillain-Barre syndrome associated with Zika virus infection. Lancet. 2016;387(10026):1482. Epub 2016/04/27. doi: 10.1016/S0140-6736(16)30058-7. PubMed PMID: 27115821.

119. Kassavetis P, Joseph JM, Francois R, Perloff MD, Berkowitz AL. Zika virus-associated Guillain-Barre syndrome variant in Haiti. Neurology. 2016;87(3):336-7. Epub 2016/05/11. doi: 10.1212/WNL.0000000000002759. PubMed PMID: 27164708.

120. Duijster JW, Goorhuis A, van Genderen PJ, Visser LG, Koopmans MP, Reimerink JH, et al. Zika virus infection in 18 travellers returning from Surinam and the Dominican Republic, The Netherlands, November 2015-March 2016. Infection. 2016. Epub 2016/05/23. doi: 10.1007/s15010-016-0906-y. PubMed PMID: 27209175.

121. van den Berg B, van den Beukel JC, Alsma J, van der Eijk AA, Ruts L, van Doorn PA, et al. [Guillain-Barre syndrome following infection with the Zika virus]. Ned Tijdschr Geneeskd. 2016;160(0):D155. Epub 2016/05/28. PubMed PMID: 27229696.

122. Yung CFT, K. C. Guillain-Barre Syndrome and Zika Virus: Estimating Attributable Risk to Inform Intensive Care Capacity Preparedness. Clin Infect Dis. 2016. Epub 2016/05/27. doi: 10.1093/cid/ciw355. PubMed PMID: 27225243.

123. Jackson BR, Zegarra JA, Lopez-Gatell H, Sejvar J, Arzate F, Waterman S, et al. Binational outbreak of Guillain-Barre syndrome associated with Campylobacter jejuni infection, Mexico and USA, 2011. Epidemiol Infect. 2014;142(5):1089-99. doi: 10.1017/S0950268813001908. PubMed PMID: 23924442.

S6 Table

124. McCarthy N, Giesecke J. Incidence of Guillain-Barre syndrome following infection with Campylobacter jejuni. Am J Epidemiol. 2001;153(6):610-4. PubMed PMID: 11257070.

[125 not part of this body of evidence]

126. Mishu B, Blaser MJ. Role of infection due to Campylobacter jejuni in the initiation of Guillain-Barre syndrome. Clin Infect Dis. 1993;17(1):104-8. PubMed PMID: 8353228.

127. Rees JH, Soudain SE, Gregson NA, Hughes RA. Campylobacter jejuni infection and Guillain-Barre syndrome. N Engl J Med. 1995;333(21):1374-9. doi: 10.1056/NEJM199511233332102. PubMed PMID: 7477117.

128. Carod-Artal FJ, Wichmann O, Farrar J, Gascon J. Neurological complications of dengue virus infection. Lancet Neurol. 2013;12(9):906-19. Epub 2013/08/21. doi: 10.1016/S1474-4422(13)70150-9. PubMed PMID: 23948177.

129. Sejvar JJ, Bode AV, Marfin AA, Campbell GL, Ewing D, Mazowiecki M, et al. West Nile virus-associated flaccid paralysis. Emerg Infect Dis. 2005;11(7):1021-7. doi: 10.3201/eid1107.040991. PubMed PMID: 16022775; PubMed Central PMCID: PMC3371783.

130. Xiang JY, Zhang YH, Tan ZR, Huang J, Zhao YW. Guillain-Barre syndrome associated with Japanese encephalitis virus infection in China. Viral Immunol. 2014;27(8):418-20. doi: 10.1089/vim.2014.0049. PubMed PMID: 25140441.

131. Ravi V, Taly AB, Shankar SK, Shenoy PK, Desai A, Nagaraja D, et al. Association of Japanese encephalitis virus infection with Guillain-Barre syndrome in endemic areas of south India. Acta Neurol Scand. 1994;90(1):67-72. PubMed PMID: 7941960.

132. McMahon AW, Eidex RB, Marfin AA, Russell M, Sejvar JJ, Markoff L, et al. Neurologic disease associated with 17D-204 yellow fever vaccination: a report of 15 cases. Vaccine. 2007;25(10):1727-34. doi: 10.1016/j.vaccine.2006.11.027. PubMed PMID: 17240001.

133. Solomon T, Kneen R, Dung NM, Khanh VC, Thuy TT, Ha DQ, et al. Poliomyelitis-like illness due to Japanese encephalitis virus. Lancet. 1998;351(9109):1094-7. doi: 10.1016/S0140-6736(97)07509-0. PubMed PMID: 9660579.

134. Chung CC, Lee SS, Chen YS, Tsai HC, Wann SR, Kao CH, et al. Acute flaccid paralysis as an unusual presenting symptom of Japanese encephalitis: a case report and review of the literature. Infection. 2007;35(1):30-2. doi: 10.1007/s15010-007-6038-7. PubMed PMID: 17297587.

135. Argall KG, Armati PJ, King NJ, Douglas MW. The effects of West Nile virus on major histocompatibility complex class I and II molecule expression by Lewis rat Schwann cells in vitro. J Neuroimmunol. 1991;35(1-3):273-84. PubMed PMID: 1955569.

S6 Table

136. Loshaj-Shala A, Regazzoni L, Daci A, Orioli M, Brezovska K, Panovska AP, et al. Guillain Barre syndrome (GBS): new insights in the molecular mimicry between C. jejuni and human peripheral nerve (HPN) proteins. J Neuroimmunol. 2015;289:168-76. doi: 10.1016/j.jneuroim.2015.11.005. PubMed PMID: 26616887.

137. Meyer Sauteur PM, Huizinga R, Tio-Gillen AP, Roodbol J, Hoogenboezem T, Jacobs E, et al. Mycoplasma pneumoniae triggering the Guillain-Barre syndrome: A case-control study. Ann Neurol. 2016. doi: 10.1002/ana.24755. PubMed PMID: 27490360.

138. Tseng YF, Wang CC, Liao SK, Chuang CK, Chen WJ. Autoimmunity-related demyelination in infection by Japanese encephalitis virus. J Biomed Sci. 2011;18:20. doi: 10.1186/1423-0127-18-20. PubMed PMID: 21356046; PubMed Central PMCID: PMC3056755.
