# Supplementary material for: Zika Virus Infection as a Cause of Congenital Brain Abnormalities and Guillain–Barré Syndrome: Systematic Review
Source: PLoS Med. 2017 Jan 3;14(1):e1002203. doi: 10.1371/journal.pmed.1002203 (PMC5207634; doi:10.1371/journal.pmed.1002203)
Supplement: S7 Table — (PDF) [file pmed.1002203.s010.pdf]

S7 Table

S7 Table. Assessment of quality of the body of evidence about Zika virus infection and Guillain-Barré syndrome

**Domain**[a]	**Assessment**
Evidence reviewed: 9 case reports, 5 case series, 1 case-control study, 19 ecological studies, 2 sequence analysis study	
Risk of bias	Human studies: Uncontrolled studies and ecological studies formed the majority of the evidence for assessing temporality. Within this group four studies showed laboratory evidence of ZIKV infection before detection of GBS symptoms. We found risks of bias in one case-control study; cases and controls were matched, but there was no additional adjustment for confounding.
Imprecision	The effect estimate in the case-control study has extremely wide confidence intervals.
Inconsistency	See S6 Table, dimension 10 (consistency)
Publication bias	Could not be assessed formally and cannot be excluded. The expert panel was not aware of studies that we missed. Our search strategy identified reports of countries that had experienced ZIKV outbreaks but had not reported an increased number of GBS cases.
Indirectness	No serious indirectness. The markers identified in *in silico* studies cannot be extrapolated directly to humans.
Magnitude of effect	See S6 Table, dimension 5 (strength of association)
Opposing plausible residual bias and confounding	None identified
Dose effect	See S6 Table, dimension 4 (dose-response relationship)

[a] Domains from GRADE working group, assessed as suggested for urgent situations (reference 20 in main text) [1]. Some of these overlap with causality dimensions so assessment not repeated

References

1. Thayer KA, Schunemann HJ. Using GRADE to respond to health questions with different levels of urgency. Environ Int. 2016;92-93:585-9. doi: 10.1016/j.envint.2016.03.027. PubMed PMID: 27126781.
