# Supplementary figures and images for: Zika Virus Infection as a Cause of Congenital Brain Abnormalities and Guillain–Barré Syndrome: Systematic Review
Source: PLoS Med. 2017 Jan 3;14(1):e1002203. doi: 10.1371/journal.pmed.1002203 (PMC5207634; doi:10.1371/journal.pmed.1002203)

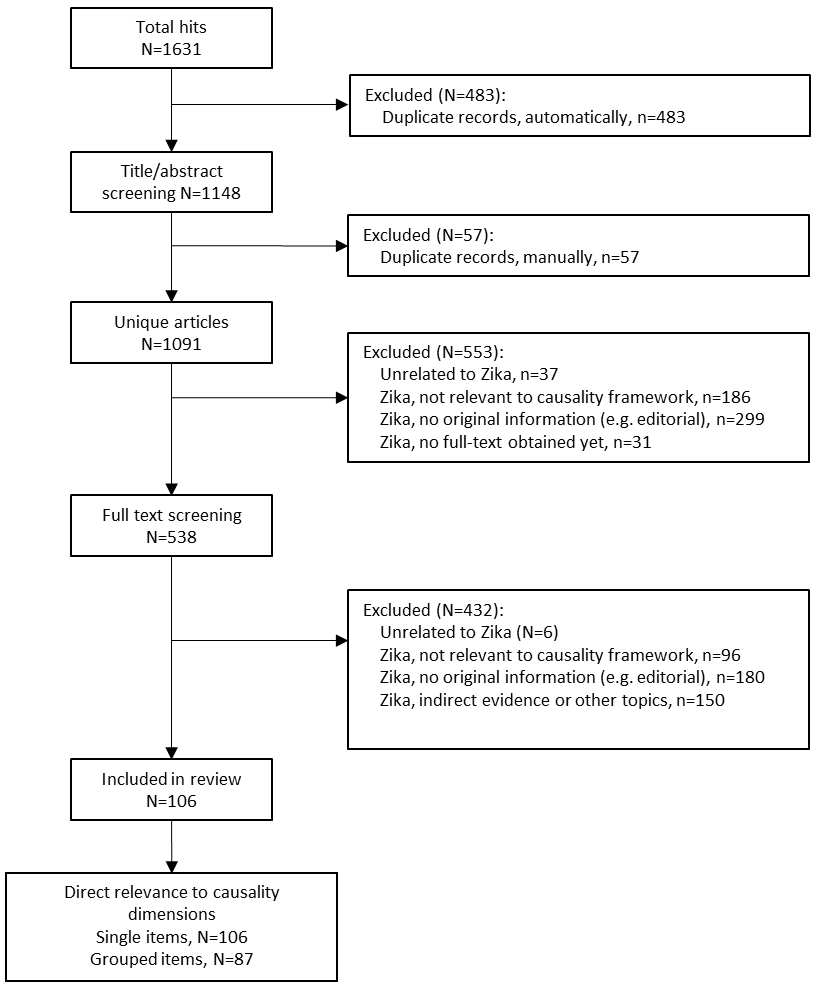

Supplement: S1 Fig — (TIF) [file pmed.1002203.s011.tif]
